# Autophagy regulator ATG5 preserves cerebellar function by safeguarding its glycolytic activity

**DOI:** 10.1038/s42255-024-01196-4

**Published:** 2025-01-15

**Authors:** Janine Tutas, Marianna Tolve, Ebru Özer-Yildiz, Lotte Ickert, Ines Klein, Quinn Silverman, Filip Liebsch, Frederik Dethloff, Patrick Giavalisco, Heike Endepols, Theodoros Georgomanolis, Bernd Neumaier, Alexander Drzezga, Guenter Schwarz, Bernard Thorens, Graziana Gatto, Christian Frezza, Natalia L. Kononenko

**Affiliations:** 1 CECAD Excellence Center, University of Cologne, Cologne, Germany; 2 Center for Physiology and Pathophysiology, Faculty of Medicine and University Hospital Cologne, Cologne, Germany; 3 Department of Neurology, University Hospital of Cologne, Cologne, Germany; 4 Institute of Biochemistry, Department of Chemistry, University of Cologne, Cologne, Germany; 5 Max Planck Institute for Biology of Ageing, Cologne, Germany; 6 Department of Nuclear Medicine, Faculty of Medicine, University Hospital Cologne, Cologne, Germany; 7 Institute of Radiochemistry and Experimental Molecular Imaging, Faculty of Medicine and University Hospital Cologne, University of Cologne, Cologne, Germany; 8 Forschungszentrum Jülich GmbH, Institute of Neuroscience and Medicine, Nuclear Chemistry (INM-5), Jülich, Germany; 9 Forschungszentrum Jülich GmbH, Institute of Neuroscience and Medicine, Molecular Organization of the Brain (INM-2), Jülich, Germany; 10 German Center for Neurodegenerative Diseases (DZNE), Bonn-Cologne, Germany; 11 Center for Molecular Medicine Cologne (CMMC), Faculty of Medicine and University Hospital Cologne, University of Cologne, Cologne, Germany; 12 Center for Integrative Genomics, Faculty of Biology and Medicine, University of Lausanne, Lausanne, Switzerland; 13 Institute for Genetics, Faculty of Mathematics and Natural Sciences, University of Cologne, Cologne, Germany

**Keywords:** Cellular neuroscience, Diseases of the nervous system, Metabolism, Metabolomics

## Abstract

Dysfunctions in autophagy, a cellular mechanism for breaking down components within lysosomes, often lead to neurodegeneration. The specific mechanisms underlying neuronal vulnerability due to autophagy dysfunction remain elusive. Here we show that autophagy contributes to cerebellar Purkinje cell (PC) survival by safeguarding their glycolytic activity. Outside the conventional housekeeping role, autophagy is also involved in the ATG5-mediated regulation of glucose transporter 2 (GLUT2) levels during cerebellar maturation. Autophagy-deficient PCs exhibit GLUT2 accumulation on the plasma membrane, along with increased glucose uptake and alterations in glycolysis. We identify lysophosphatidic acid and serine as glycolytic intermediates that trigger PC death and demonstrate that the deletion of GLUT2 in ATG5-deficient mice mitigates PC neurodegeneration and rescues their ataxic gait. Taken together, this work reveals a mechanism for regulating GLUT2 levels in neurons and provides insights into the neuroprotective role of autophagy by controlling glucose homeostasis in the brain.

## Abstract (web summary)

Tutas et al. show an unconventional role for autophagy in the regulation of glycolytic flux in cerebellar Purkinje cells by maintaining the levels of the glucose transporter GLUT2.

## Main

Autophagy is a conserved lysosomal pathway that recycles damaged or unnecessary cellular components^1^. The most common form of autophagy is macroautophagy (hereafter autophagy). In this process, parts of the cytoplasm and cargo destined for degradation are enclosed in double-membrane vesicles called autophagosomes, which are then delivered to lysosomes for degradation. Two ubiquitin-like conjugating systems, ATG12–ATG5–ATG16L1 and LC3-I/LC3-II, are essential for the elongation and closure of the autophagosome membrane^2^, and the absence of their components, such as ATG5, results in autophagy inhibition^3^. Recent research has increasingly focused on autophagy’s role in the brain due to its association with neurodegeneration^4–7^. However, the precise mechanisms by which impaired autophagy triggers neurodegeneration remain unanswered.

Autophagy operates continuously at low levels, clearing toxic proteins and damaged organelles as part of cellular quality control^8^. It also plays a supportive role in cellular metabolism^9^, especially during nutrient deprivation, where it ensures the availability of survival-critical biomolecules^10,11^. For instance, newborn mice require autophagy to maintain serum amino acid (AA) levels during starvation^12,13^, while in adults, it helps to sustain serum glucose levels during fasting^14^. Autophagy can also regulate metabolism independently of starvation, particularly in tumour cells^15,16^. Autophagy-deficient cancer cells exhibit increased glycolytic metabolism even under nutrient-rich conditions^17^, utilizing aerobic glycolysis to convert more glucose to lactate^18^. This metabolic switch is essential for cancer cell growth, and may be achieved in part through the role of autophagy in the recycling of key proteins of the glycolytic pathway^19–21^. Methylglyoxal (MG), a by-product of glycolysis, is a reactive dicarbonyl that can glycate proteins and lipids, leading to carbonyl stress—a hallmark of ageing^22^. Elevated levels of carbonyl proteins have been detected in individuals with age-associated neurodegenerative diseases, including Parkinson’s disease (PD) and Alzheimer’s disease (AD)^23,24^. Intriguingly, a metabolic switch to aerobic glycolysis has been recently shown to underlie the neurodegeneration in sporadic AD patient-derived neurons^25^. Additionally, autophagy is compromised during ageing^26^, but whether autophagy can serve a neuroprotective role by safeguarding glycolytic metabolism in the brain is yet to be determined.

The most recognized function of autophagy in neuronal metabolism is the degradation of damaged and/or aged mitochondria, a process known as mitophagy^27^. However, our understanding of the housekeeping-independent role of autophagy in brain metabolism is still limited when compared to non-neuronal cells. Recently, we found that the crucial autophagy modifier ATG5 operates in cortical synapses to maintain functional cAMP–PKA signalling, but is not essential for the survival of excitatory cortical^28^ and/or inhibitory cortical and striatal neurons^29^, in line with findings from several other studies^30,31^. This stands in stark contrast to the progressive loss of PCs in the cerebellum observed in mice after ATG5 deletion^32^. The mechanism behind the selective vulnerability of PCs under conditions of defective autophagy remains currently elusive.

Here, we find that the core autophagy protein ATG5 regulates PC survival beyond its conventional role in clearing protein aggregates and mitochondria. Instead, ATG5 acts neuroprotective by controlling the glycolytic activity. Utilizing a mouse model with targeted ATG5 deficiency in inhibitory neurons and using a comprehensive approach involving positron emission tomography (PET) imaging, quantitative proteomics and metabolomics, and in-depth kinematic analysis, we demonstrate that autophagy in PCs downregulates GLUT2 levels during brain maturation. Autophagy-deficient PCs accumulate GLUT2 on their plasma membrane, leading to increased glucose uptake and higher glycolytic flux. This results in elevated non-mitochondrial ATP, increased lactate production and heightened levels of MG-modified proteins. We identify several glycolytic products, including serine and lysophosphatidic acid (LPA), that are elevated in autophagy-deficient cerebellum and toxic to PCs. Remarkably, the deletion of GLUT2 in ATG5-deficient mice mitigates PC neurodegeneration and ameliorates the ataxic gait. Our results demonstrate that the neuroprotective functions of autophagy in the brain include its role in preventing excessive glycolytic metabolism.

## Results

### Differential vulnerability of inhibitory neurons to ATG5 loss

**Fig. 1:**
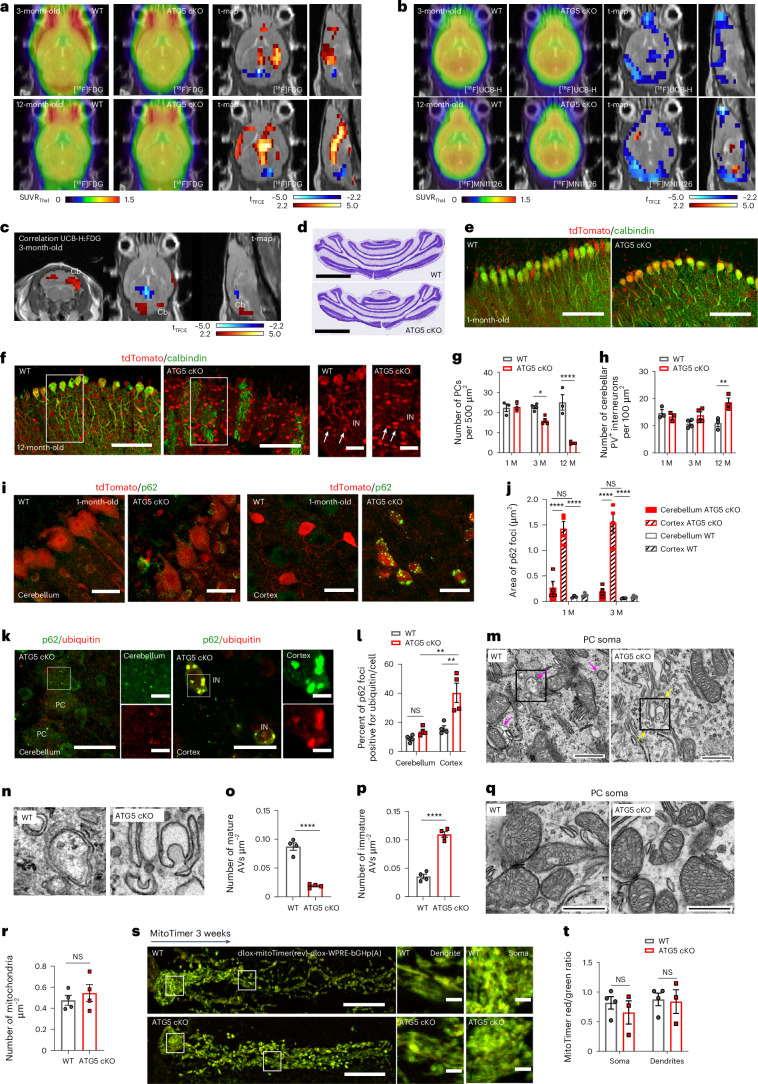
Autophagy loss increases PC vulnerability, independent of its role in protein and mitochondria quality control. **a**,**b**, [^18^F]FDG-PET (**a**) and [^18^F]UCB-H and [^18^F]MNI1126-PET (**b**) imaging in WT and ATG5 cKO mice (WT 3 months (3 M): *N* = 7; 12 months (12 M): *N* = 6; cKO: *N* = 6 mice per group). T-map with voxel-wise comparison (two-tailed *t*-test, corrected for multiple testing) is shown to the right. Significant changes (*P* < 0.05) in cKO mice are indicated in red (higher) and blue (lower). **c**, Pearson correlation between [^18^F]UCB-H- and [^18^F]FDG-PET signals for ATG5 cKO mice (two-tailed *t*-test corrected for multiple testing). **d**, Nissl-stained WT and ATG5 cKO cerebellum at 3 M. Scale bars, 2 mm. **e**–**g**, PC density in WT and ATG5 cKO cerebellum, immunostained for calbindin in 1 M (**e**) and 12 M (**f**) mice. **g**, Quantification of PC density. INs, interneurons. Scale bars, 100 µm; magnified image scale bars, 25 µm. 1 M and 12 M: *N* = 3 mice; 3 M: *N* = 4. WT^3M^ versus cKO^3M^: *P* = 0.015; WT^12M^ versus cKO^12M^: *P* < 0.0001. **h**, Interneuron density in WT and ATG5 cKO cerebellum. 1 M and 12 M: *N* = 3 mice; 3 M: *N* = 4. WT^12M^ versus cKO^12M^: *P* = 0.002. **i**, Cerebellum and cortex from WT and ATG5 cKO mice, immunostained for p62. Scale bars, 25 µm. **j**, p62 foci area in PCs and cortical interneurons of WT and ATG5 cKO animals. *N* = 4 mice for cKO both ages and 3 M WT; *N* = 3 mice for 1 M WT. *P* < 0.0001. **k**, Cerebellum (left) and cortex (right) from WT and ATG5 cKO mice at 3 M, immunostained for p62 and ubiquitin. Scale bars, 25 µm; magnified image scale bars, 5 µm. **l**, Percentage of ubiquitin-positive p62 puncta in the cortex and cerebellum of WT and ATG5 cKO mice at 3 M. *N* = 4 mice per genotype. cKO^Cerebellum^ versus cKO^Cortex^ *P* = 0.001; WT^Cortex^ versus cKO^Cortex^ *P* = 0.002. **m**–**p**, EM analysis of mature and immature autophagosomes (AVs) in WT and ATG5 cKO PCs. **m**, Magenta arrowheads mark mature AVs or a mitophagy event. Yellow arrowheads indicate immature AVs. Scale bars, 1 µm. *N* = 4 mice per genotype. **n**, Magnified images of mature and immature AVs shown in **m**. **o**,**p**, Quantitative analysis of mature (**o**) and immature (**p**) AVs; mature AVs: *P* < 0.0001, immature AVs, *P* < 0.0001. Statistical significance calculated by unpaired two-tailed *t*-test. **q**,**r**, EM images (**q**) and quantitative analysis (**r**) of mitochondrial density in WT and ATG5 cKO PCs at 3 M. Scale bars, 0.5 µm. *N* = 4 mice per genotype. Statistical significance calculated by unpaired two-tailed *t*-test. **s**, Analysis of mitochondrial turnover in mitoTimer-expressing WT and ATG5 cKO PCs at 3 M. Scale bars, 20 µm; magnified image scale bars, 1 µm. **t**, Quantitative analysis of data in **s**. *N* = 4 WT/3 cKO. 1 M, 3 M and 12 M indicate 1, 3 and 12 months of age, respectively. Squares in **f**, **m** and **s** indicate regions magnified. All graphs show the mean ± s.e.m. Statistical significance in **g**, **h**, **j**, **l** and **t** was determined by two-way analysis of variance (ANOVA) followed by Holm–Sidak multiple-comparisons test. NS, not significant; *P ≤ 0.05; **P ≤ 0.01; ***P ≤ 0.001; ****P ≤ 0.0001. Source data

**Extended Data Fig. 1:**
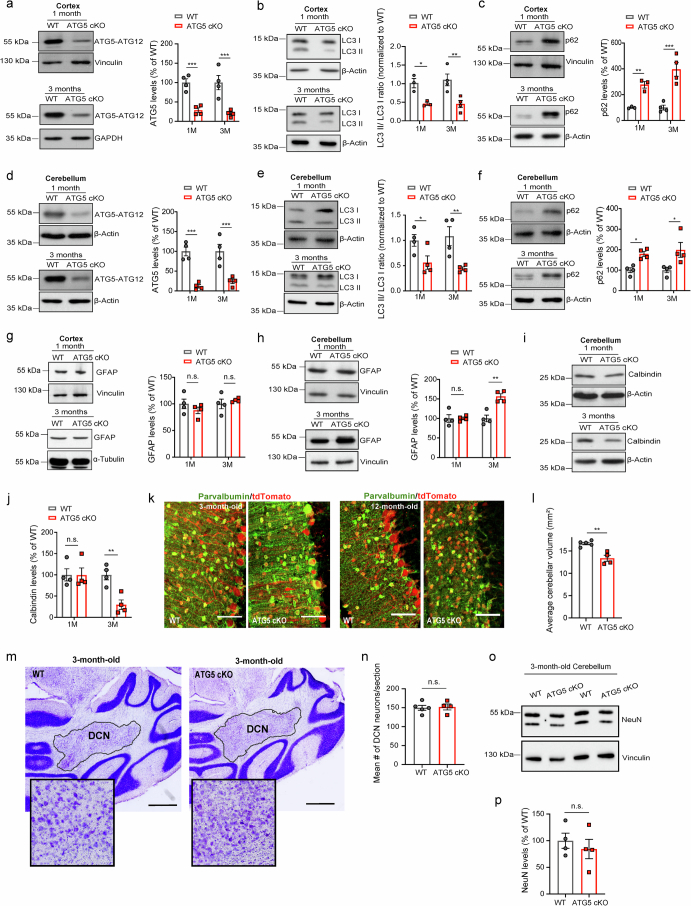
Impaired autophagy in ATG5 cKO cerebellum without DCN or interneuron loss. **a**, ATG5 ~ ATG12 protein levels in WT and ATG5 cKO cortical lysates. N = 4 mice for each genotype and condition. 1 M: p = 0.0006, 3 M: p = 0.0005. **b**, LC3 II/LC3 I ratio in WT and ATG5 cKO cortical lysates. N = 3 mice for each genotype for 1 M and N = 4 for 3 M. 1 M: p = 0.013, 3 M: p = 0.004. **c**, p62 protein levels in WT and ATG5 cKO cortical lysates. N = 3 mice for each genotype for 1 M and N = 4 for each genotype for 3 M. 1 M: p = 0.008, 3 M: p = 0.0002. **d**, ATG5 ~ ATG12 protein levels in WT and ATG5 cKO cerebellar lysates. N = 4 for each genotype and condition. 1 M: p = 0.0002, 3 M: p = 0.0004. **e**, LC3 II/LC3 I ratio in WT and ATG5 cKO cerebellar lysates. N = 4 mice for each genotype and condition. 1 M: p = 0.033, 3 M: p = 0.008. **f**, p62 protein levels in WT and ATG5 cKO cerebellar lysates. N = 4 mice for each genotype and condition. 1 M: p = 0.019, 3 M: p = 0.011. **g**, GFAP levels in WT and ATG5 cKO cortical lysates. N = 4 mice for each genotype and condition. **h**, GFAP levels in WT and ATG5 cKO cerebellar lysates. N = 4 mice for each genotype and condition. 3 M: p = 0.008. **i,j**, Calbindin protein levels in WT and ATG5 cKO cerebellar lysates. N = 4 mice for each genotype and condition. 3 M: p = 0.007. **k**, WT and ATG5 cKO cerebellum, immunostained for parvalbumin. Scale bar, 200 µm. **l**, Average volume analysis of WT and ATG5 cKO cerebellum at 3 M based on Nissl staining. N = 5 for WT and N = 4 for ATG5 cKO mice. Two-tailed unpaired t-Test: p = 0.002. **m,n**, Cell density in the deep cerebellar nuclei (DCN) in WT and ATG5 cKO cerebellum. N = 5 for WT and N = 4 for ATG5 cKO mice. Two-tailed unpaired t-Test. **o,p** NeuN levels in WT and ATG5 cKO cerebellar lysates. N = 4 mice per genotype. Two-tailed unpaired t-Test. 1 M and 3 M indicate 1 and 3 months, respectively. All graphs show mean ± SEM. Statistical significance in a-j was determined by Two-way ANOVA followed by Holm-Šidák multiple comparisons. n.s.—non-significant; * indicates P ≤ 0.05; ** indicates P ≤ 0.01; *** indicates P ≤ 0.001; **** indicates P ≤ 0.0001. Source data

To understand how autophagy contributes to PC degeneration, we capitalized on the previously published mouse line lacking the crucial autophagy component ATG5 in inhibitory neurons (*Atg5*^fl/fl^:*Slc32a1-*Cre^tg^ knockout (KO) mice, further defined as ATG5 conditional knockout (cKO) mice)^29^ (Extended Data Fig. 1a). In these mice, the inhibition of autophagy is reflected in significantly increased p62 levels and downregulated autophagosomal LC3 levels in the cortex and cerebellum as early as 1 month of age (Extended Data Fig. 1b–f). We previously observed no loss of forebrain GABAergic neurons in 3-month-old ATG5 cKO mice^29^. To conduct an unbiased analysis of ATG5 cKO mouse brains in vivo, we performed longitudinal PET imaging, using the [^18^F]-fluorodeoxyglucose (FDG) radiotracer, an indirect reporter of neurodegeneration. In agreement with our previous work^29^, we found only minor changes in the cortical and/or striatal FDG-PET signal in cKO mice compared to control mice (Fig. 1a). In contrast, a pronounced reduction in FDG uptake was detected in the ATG5 cKO cerebellum, suggesting a decrease in neuronal activity in this region. These changes already occurred at the age of 3 months and persisted until the age of 12 months. The decrease in FDG-PET signal may reflect not only changes in neurodegeneration but also alterations in glucose uptake in the cerebellum. To more precisely assess neurodegenerative changes in ATG5 cKO mice, we measured synapse density using the PET radiotracers [^18^F]-UCB-H and/or [^18^F]-MNI1126 that bind to synaptic vesicle glycoprotein 2A (SV2A; Fig. 1b). The SV2A-PET signal was significantly reduced in the cerebellum and cortex of 3-month-old and 12-month-old ATG5 cKO animals compared to their control littermates (that is, *Atg5*^wt/wt^: *Slc32a1-*Cre^tg^ mice, further defined as wild-type (WT) mice). Notably, the decrease in SV2A-PET signal in the cerebellum positively correlated with reduced [^18^F]-FDG-PET (Fig. 1c), suggesting that the decreased FDG-PET signal is at least partly due to the loss of synapses in this region. In agreement with this finding, we detected no alterations in glial fibrillary acidic protein levels (a marker of astrogliosis) in the ATG5 cKO cortex (Extended Data Fig. 1g), whereas glial fibrillary acidic protein was significantly increased in the cerebellum of 3-month-old autophagy-deficient mice (Extended Data Fig. 1h).

**Extended Data Fig. 2:**
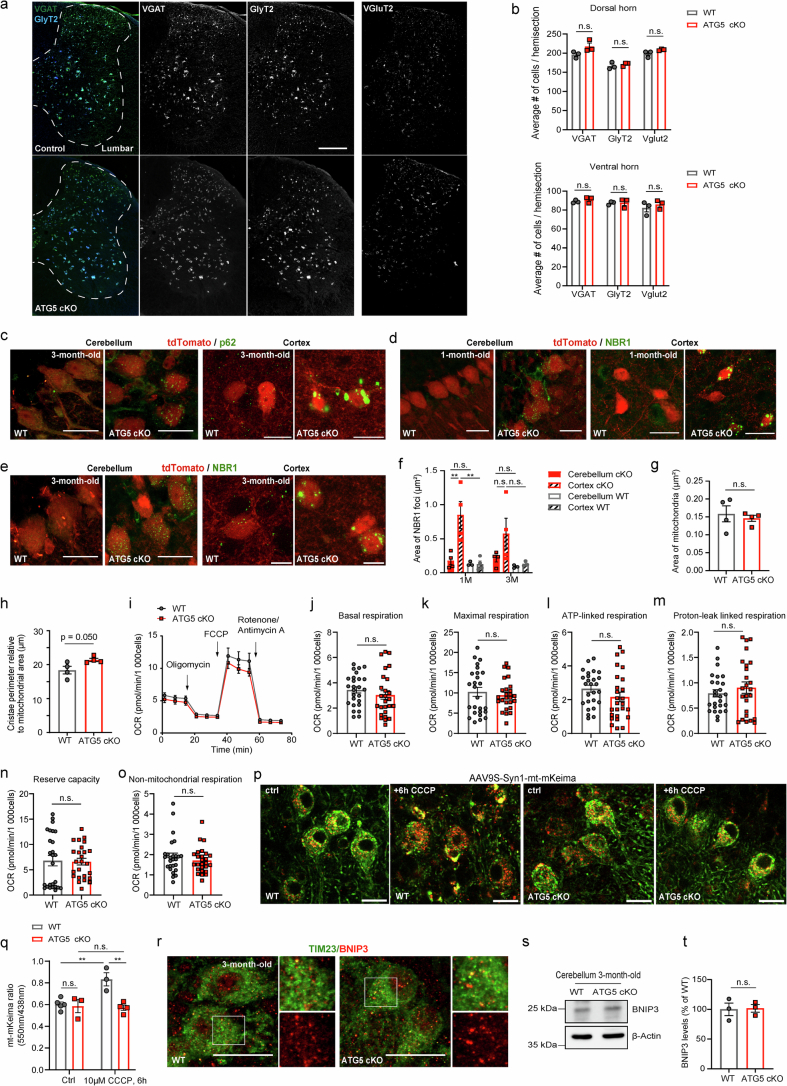
Basal mitochondrial function is intact in ATG5 cKO cerebellum. **a,b**, RNAscope *in situ* hybridization examples (a) and cell density analysis in the cervical and lumbar spinal cord segments (b) of WT and ATG5 cKO animals at 3 M for probes against VGAT, GlyT2 or VGluT2 mRNAs. Scale bar, 200 µm. N = 3 mice per genotype. Multiple t test with two-stage linear step-up procedure of Benjamini, Krieger and Yekutieli, with Q = 1%. **c**, Cerebellum and cortex of WT and ATG5 cKO mice at 3 M, immunostained for p62. Scale bar, 25 µm. See quantification in Fig. 1j (N = 4 for cKOs both ages and 3 M WT mice, N = 3 for 1 M WT mice). **d**-**f**, Cerebellum and cortex of WT and ATG5 cKO mice at 1 M (d) and 3 M (e), immunostained for NBR1. Scale bar, 25 µm. Fluorescence analysis of the area of NBR1 foci in PCs and cortical interneurons (f). N = 4 mice per genotype for ATG5 cKO^1&3M^ and WT^1M^, and N = 3 for WT^3M^. Two-way ANOVA followed by Holm-Šidák multiple comparisons test (1 M cKO^cerebellum^ vs cKO^cortex^: p = 0.003; 1M cKO^cortex^ vs WT^cortex^: p = 0.002). **g**, Mitochondrial area in WT and ATG5 cKO PCs at 3 M. N = 4 mice for each genotype. Two-tailed unpaired t-Test. **h**, Mitochondrial cristae perimeter normalized to mitochondrial area in WT and ATG5 cKO PCs at 3 M. N = 4 mice for each genotype. Two-tailed unpaired t-Test. **i-o**, OCR rates during Seahorse XF Cell Mito Stress test in WT and ATG5 cKO primary cerebellar cells with injections of 1.5 µM Oligomycin A, 2 µM FCCP and 0.5 µM Rotenone/Antimycin A. N = 5 cultures per genotype with 26 wells with ± 20 000 cells. Two-tailed unpaired t-Test. **p,q**, mt-mKeima ratio in WT and ATG5 cKO PCs in OTCs at baseline or after 6 h incubation with 10 µM CCCP. Scale bar, 20 µm. N = 5 WT/N = 3 ATG5 cKO mice for control and N = 3 WT/ N = 4 ATG5 cKO for CCCP. Two-way ANOVA followed by Holm-Šidák multiple comparisons test (WT^control^ vs WT^CCCP^: p = 0.007, WT^CCCP^ vs cKO ^CCCP^ p = 0.005). **r**, Example confocal images of WT and ATG5 cKO cerebellum, immunostained for TIM23 and BNIP3 from N = 1 experiment. Scale bar, 25 µm. **s,t**, BNIP3 protein levels in WT and ATG5 cKO cerebellar lysates. N = 3 per genotype. Two-tailed unpaired t-Test. 1 M and 3 M indicate 1 and 3 months of age, respectively. All graphs show mean ± SEM. n.s.—non-significant; * indicates P ≤ 0.05; ** indicates P ≤ 0.01; *** indicates P ≤ 0.001; **** indicates P ≤ 0.0001. Source data

Cerebellar atrophy and loss of cerebellar PCs were reported in several mouse models with autophagy deficiency^32–34^. Thus, we analysed the number of PCs in WT and ATG5 cKO mice, additionally carrying the tdTomato allele (*Ai9*) as a reporter (that is, *Atg5*^wt/wt^: *Slc32a1-*Cre^tg^: *Ai9* and *Atg5*^fl/fl^: *Slc32a1-*Cre^tg^: *Ai9* mice). We found that the cerebellum was smaller (Fig. 1d and Extended Data Fig. 1l) and contained significantly less calbindin-positive PCs in 3-month-old ATG5 cKO mice compared to controls (Fig. 1e–g and Extended Data Fig. 1i,j; see also Fig. 7j). The loss of PCs was not due to developmental defects, as their numbers were comparable in the cerebellum of 1-month-old WT and ATG5 cKO mice (Fig. 1e,g), and was progressive, with only a few PCs detected in the cerebellum lacking ATG5 at 12 months of age (Fig. 1f,g). This cell loss was selective for PCs, as inhibitory GABAergic interneurons remained present in the cerebellum of ATG5 cKO mice (Fig. 1f and Extended Data Fig. 1k), and their number was even significantly increased in 12-month-old animals (Fig. 1h). Additionally, the density of neurons in the deep cerebellar nuclei (Extended Data Fig. 1m,n) and levels of NeuN (which is expressed in most cerebellar neurons except PCs)^35^ (Extended Data Fig. 1o,p) were unchanged in ATG5 cKO mice. Because the *Slc32a1*-Cre driver line also targets glycinergic neurons in the spinal cord, we examined their density in the spinal cord and found no changes in the number of glycinergic, GABAergic or glutamatergic interneurons (Extended Data Fig. 2a,b). These findings strongly suggest that different types of neurons across brain regions exhibit distinct levels of vulnerability to autophagy loss.

### Inverse correlation of protein aggregates and neurodegeneration

To better understand the selective vulnerability of cerebellar PCs to autophagy dysfunction, we first assessed autophagy defects by imaging p62 aggregates using immunohistochemistry (IHC). We found that, although p62 levels were significantly increased at 1 and 3 months in both the ATG5 cKO cortex and cerebellum (Extended Data Fig. 1c,f), p62 was significantly less aggregated in characteristic foci in autophagy-deficient PCs (Fig. 1i,j and Extended Data Fig. 2c). A similar pattern was observed with another autophagy receptor NBR1 (Extended Data Fig. 2d–f). In contrast, p62 and NBR1 puncta formed aggregates in ATG5 cKO cortical neurons (Fig. 1i,j and Extended Data Fig. 2c–f). By 3 months of age, approximately 40% of these p62-positive foci in cortical ATG5 cKO neurons were also positive for ubiquitin, whereas fewer than 15% of autophagy-deficient PCs exhibited ubiquitin-positive p62 puncta (Fig. 1k,l).

Several studies have identified ATG5/ATG7-independent autophagy^36,37^. To evaluate if the absence of p62/NBR1 foci in ATG5 cKO PCs is due to the cells still being able to form autophagosomes, we analysed autophagosome numbers in both WT and ATG5 cKO PCs by electron microscopy (EM). Mature autophagosomes were abundant in WT PCs, but their number was significantly reduced in PCs lacking ATG5 (Fig. 1m–o). Conversely, unprocessed immature autophagosomes were significantly increased in ATG5 cKO PCs (Fig. 1p), indicating that the deletion of ATG5 significantly impairs autophagosome formation in PCs.

We then asked if impaired mitochondrial clearance in autophagy-deficient PCs could explain their vulnerability. Mitochondria in ATG5 cKO PCs exhibited similar morphology to WT mitochondria (Fig. 1q,r and Extended Data Fig. 2g,h), and Seahorse-based mitochondrial respiration in cultured cerebellar ATG5 cKO neurons was unaltered (Extended Data Fig. 2i–o). However, because cultured cerebellar neurons are mostly enriched in granule cells, their normal respiration might mask the mitochondrial dysfunctions of the PCs. Therefore, we specifically analysed mitochondrial turnover in autophagy-deficient PCs. Basal mitochondrial turnover measured by the mitoTimer reporter in PCs in vivo was unaffected (Fig. 1s,t). Unaltered ratios of the mitochondria-localized mKeima-Red (mt-mKeima) in ATG5 cKO PCs ex vivo (Extended Data Fig. 2p,q) along with unchanged levels of the mitophagy receptor BNIP3 (Extended Data Fig. 2r,t) further supported the absence of basal mitophagy defects in these cells. However, ATG5 was required for removing damaged mitochondria in PCs, as mitophagy in these cells was impaired after carbonyl cyanide *m*-chlorophenylhydrazone (CCCP) treatment (Extended Data Fig. 2p,q). These results suggest that while basal mitophagy in PCs may function independently of ATG5, the removal of damaged mitochondria relies on ATG5-mediated autophagy. Furthermore, the increased neurodegeneration susceptibility in autophagy-deficient PCs does not appear to result from protein aggregates or basal mitophagy issues.

### Multimodal omics reveal glycolysis changes in ATG5 cKO cerebellum

**Fig. 2:**
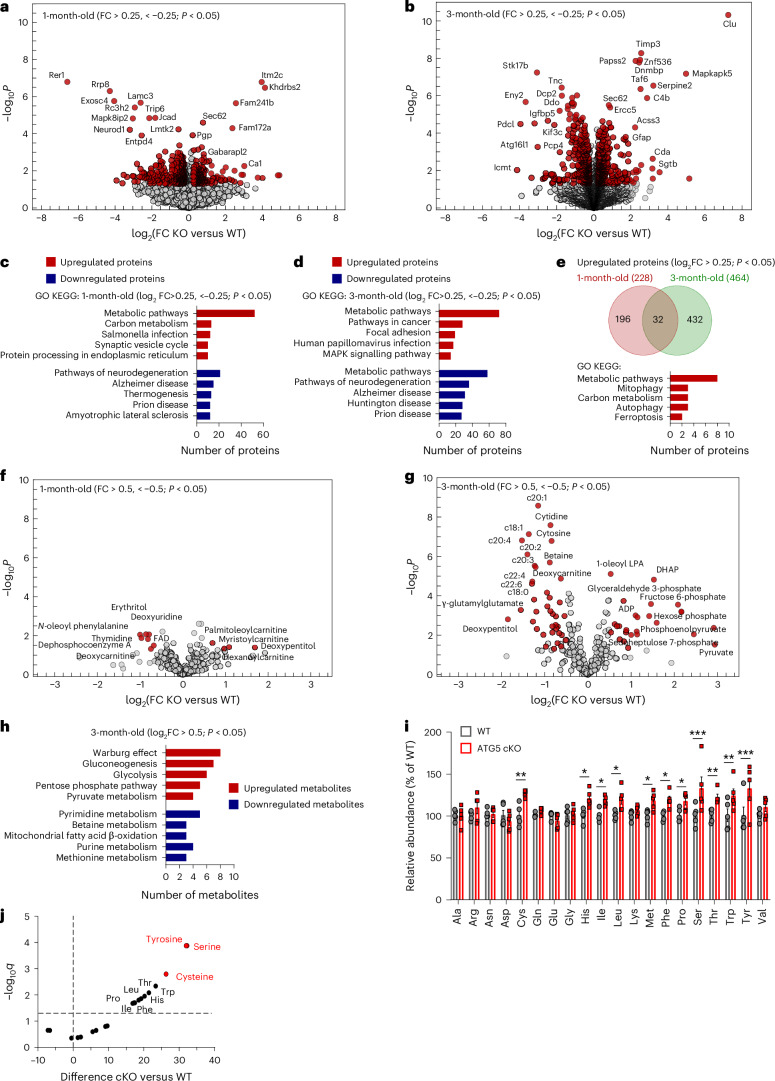
Proteomic and metabolic analyses identify metabolic rewiring in the ATG5 cKO cerebellum. **a**,**b**, Volcano plot of differentially expressed proteins in WT and ATG5 cKO cerebellum at 1 M (**a**) and 3 M (**b**). Red coloured circles mark significantly deregulated proteins at *P* < 0.05 and log_2_(fold change (FC)) of <−0.25, >0.25 (determined by two-tailed *t*-test). See also Supplementary Data 1. *N* = 5 mice per genotype. **c**,**d**, ShinyGO v0.741-based GO analysis of KEGG terms in the cerebellar proteome (*P* < 0.05 and log_2_FC of <−0.25, >0.25) at 1 M (**c**) and 3 M (**d**). **e**, Venn diagram of commonly upregulated proteins (*P* < 0.05 and log_2_FC of <−0.25, > 0.25) in the 1 M and 3 M ATG5 cKO cerebellum. **f**,**g**, Volcano plots of differentially abundant metabolites in WT and ATG5 cKO cerebellum at 1 M (**f**) and 3 M (**g**). Red coloured circles mark significantly deregulated metabolites at *P* < 0.05 and log_2_FC of <−0.5, >0.5 (determined by two-tailed *t*-test). See also Supplementary Data 2. *N* = 5 mice per genotype. **h**, MetaboAnalyst-based pathway analysis of significantly upregulated and downregulated metabolites in 3 M ATG5 cKO cerebellum (*P* < 0.05 and log_2_FC of <−0.5, >0.5). **i**, Relative AA profile in 1 M WT and ATG5 cKO cerebellum (*N* = 5 mice per genotype). Multiple unpaired two-sided *t*-tests with linear Benjamini, Krieger and Yekutieli correction. **j**, Volcano plot of the differentially abundant AAs in 1 M ATG5 cKO cerebellum shown in **i**. Red coloured circles mark the most upregulated AAs. See also Supplementary Data 3. All graphs show the mean ± s.e.m. *P ≤ 0.05; **P ≤ 0.01; ***P ≤ 0.001; ****P ≤ 0.0001. Source data

**Extended Data Fig. 3:**
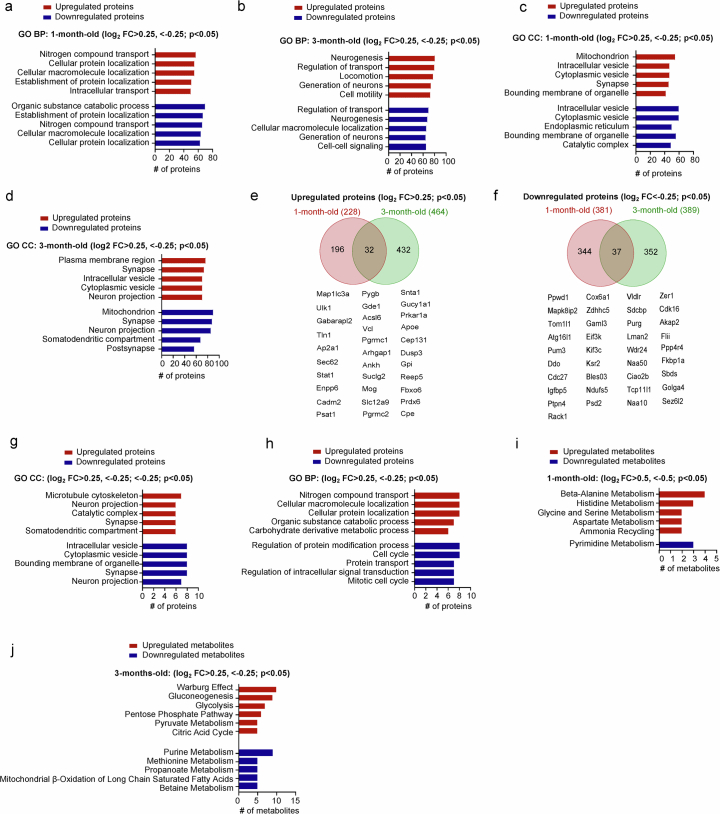
Deregulated pathways in ATG5 cKO cerebellum. **a**,**b**, ShinyGO v0.741-based GO analysis of Biological Processes (BP)-enriched terms in the cerebellar proteome (p < 0.05 and log_2_ fold change of <-0.25 > 0.25) at 1 M (a) and 3 M(b). **c,d**, ShinyGO v0.741-based GO analysis of Cellular Component (CC)-enriched terms in the cerebellar proteome (p < 0.05 and log_2_ fold change of <-0.25 > 0.25) at 1 M(c) and 3 M (d). **e,f**, Venn diagram of commonly upregulated (e) and downregulated (f) proteins (p < 0.05 and log_2_ fold change of <-0.25 > 0.25) in the 1- and 3-months-old ATG5 cKO cerebellum. **g,h**, ShinyGO v0.741-based GO analysis of Cellular Component (CC)- (g) and Biological Processes (BP) (h) enriched terms in the commonly upregulated and downregulated cerebellar proteome of ATG5 cKO (p < 0.05 and log_2_ fold change of <-0.25 > 0.25) at 1 M and 3 M. **i**, MetaboAnalyst-based pathway analysis of significantly upregulated and downregulated metabolites in ATG5 cKO cerebellum (p < 0.05 and log_2_ fold change of <-0.5 > 0.5) a 1 M. **j**, MetaboAnalyst-based pathway analysis of significantly upregulated and downregulated metabolites in ATG5 cKO cerebellum (p < 0.05 and log_2_ fold change of <-0.25 > 0.25) at 3 M. 1 M and 3 M indicate 1 and 3 months of age, respectively. Source data

To reveal the cellular mechanisms behind neurodegeneration in the ATG5 cKO cerebellum, we analysed the cerebellar proteome in WT and ATG5 cKO animals at 1 and 3 months of age (before and after neurodegeneration, respectively). We identified 609 and 853 significantly dysregulated proteins (with a log_2_ fold change <−0.25 and >0.25) in the cerebellum of ATG5 cKO mice at the ages of 1 and 3 months, respectively (Fig. 2a,b and Supplementary Data 1). Kyoto Encyclopedia of Genes and Genomes (KEGG) analysis of the upregulated proteins highlighted metabolic and carbon metabolism pathways, while the downregulated proteins clustered around neurodegenerative disease pathways (Fig. 2c,d). Gene Ontology (GO) analyses revealed alterations in protein transport and localization to membrane compartments, synapses and mitochondria (Extended Data Fig. 3a–d). Only 32 proteins were commonly upregulated in the ATG5 cKO cerebellum at 1 and 3 months, and were clustered in pathways of metabolism, autophagy and ferroptosis (Fig. 2e and Extended Data Fig. 3e), whereas the commonly downregulated proteins were linked to cellular protein localization and synapses (Extended Data Fig. 3f–h).

The proteome dataset described above suggests that the conditional loss of ATG5 in the cerebellum results in metabolic dysregulation at 1 month of age. To investigate metabolome changes in more detail, we conducted a semi-targeted metabolomic analysis. At 1 month of age, only a few metabolites were altered in the ATG5 cKO cerebellum compared to WT (Fig. 2f and Extended Data Fig. 3i). However, by 3 months of age, the ATG5 cKO cerebellum exhibited more pronounced metabolic alterations (Fig. 2g and Supplementary Data 2). Upregulated metabolites clustered in metabolic pathways associated with glucose metabolism, the pentose phosphate pathway (PPP) and pyruvate metabolism, while downregulated metabolites were associated with pyrimidine metabolism and fatty acid β-oxidation (Fig. 2h and Extended Data Fig. 3j).

The supply of AAs is crucial for cell survival^38^. Surprisingly, we found no decrease in AA content in the cerebellum of ATG5 cKO mice (Fig. 2i and Supplementary Data 3), suggesting that cerebellar AA production via autophagy likely functions primarily under nutrient-deprived conditions. Conversely, we observed a significant increase in AAs in ATG5 cKO cerebellar lysates, and, interestingly, some of these AAs can be synthesized by the intermediates of the glycolysis pathway, for instance, serine and cysteine^39^ (Fig. 2i,j). This implies that the loss of cerebellar autophagy leads to a rewiring of glycolytic metabolism.

### Cerebellar ATG5 loss boosts glucose flux and glycolysis

**Fig. 3:**
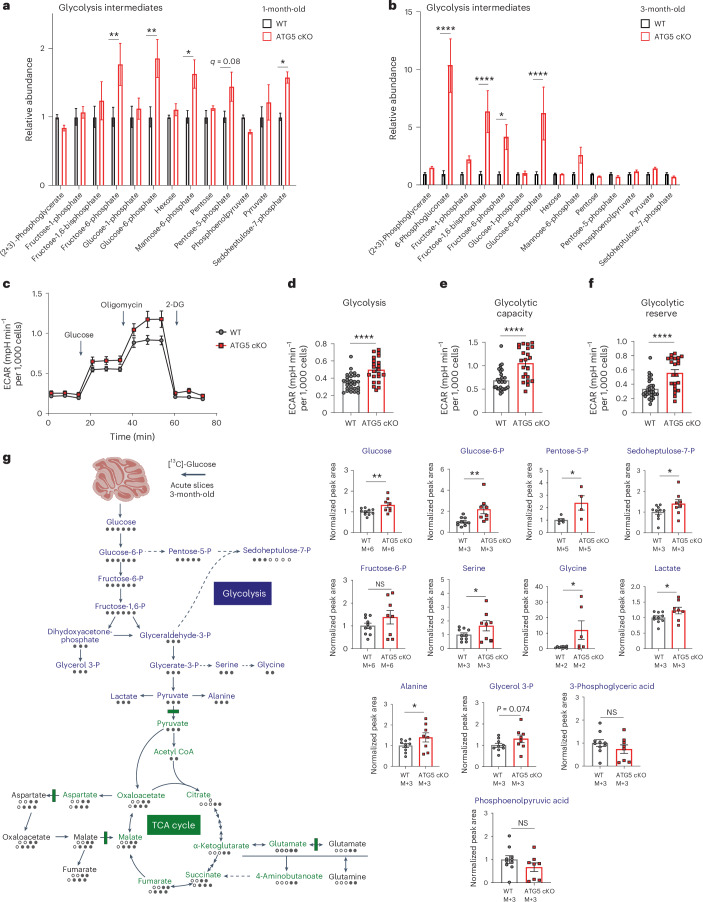
Conditional ATG5 deletion results in upregulated glycolysis and increases glucose flux in the cerebellum. **a,b**, Glycolytic and PPP intermediates in 1 M (**a**) and 3 M (**b**) WT and ATG5 cKO cerebellum (*N* = 5 mice per genotype). WT^1M^ versus cKO^1M^ glucose-6-phosphate *q* = 0.001, fructose-6-phosphate *q* = 0.002, mannose-6-phosphate *q* = 0.015, sedoheptulose-7-phosphate *q* = 0.022; WT^3M^ versus cKO^3M^ glucose-6-phosphate *q* = 0.00001, fructose-6-phosphate *q* = 0.011, fructose-1,6-biphosphate *q* = 0.00001, 6-phosphogluconate *q* < 0.000001. Multiple unpaired two-sided *t*-tests with linear Benjamini, Krieger and Yekutieli correction. **c**–**f**, ECAR rates in primary WT and ATG5 cKO cerebellar cells (**c**). Analysis of glycolysis rates (**d**) (WT versus cKO: *P* < 0.0001), glycolytic capacity (**e**) (WT versus cKO: *P* < 0.0001) and glycolytic reserve (**f**) (WT versus cKO: *P* < 0.0001); 29 wells from *N* = 5 WT mice and 22 wells from *N* = 6 cKO mice; each well contained ± 20,000 cells. Two-tailed unpaired *t*-test. **g**, Left: isotopomer representation of [^13^C]glucose incorporation into glycolysis and the TCA cycle. Black, labelled carbons; white, unlabelled carbons. Right: [^13^C]glucose-based analysis of glucose metabolic flux in 3 M WT and ATG5 cKO cerebellar slices (*N* = 10 WT, *N* = 8 cKO, values in cKO were normalized to WT). M+X, quantified isotopologue of the traced compounds; M, monotopic mass; X selected isotopologue. One-tailed unpaired *t*-test (WT^glucose^ versus cKO^glucose^: *P* = 0.007, WT^Glucose-6-P^ versus cKO^Glucose-6-P^: *P* = 0.004, WT^Pentose-5-P^ versus cKO^Pentose-5-P^: *P* = 0.017, WT^Seduheptulose-7-P^ versus cKO^Seduheptulose-7-P^: *P* = 0.037, WT^Serine^ versus cKO^Serine^: *P* = 0.044; WT^Glycine^ versus cKO^Glycine^: *P* = 0.019, WT^Lactate^ versus cKO^Lactate^: *P* = 0.03, WT^Alanine^ versus cKO^Alanine^: *P* = 0.045, WT^Glyerol-3-P^ versus cKO^Glycerol-3-P^: *P* = 0.074. Cerebellum icon created with BioRender.com. All graphs show the mean ± s.e.m. *P ≤ 0.05; **P ≤ 0.01; ***P ≤ 0.001; ****P ≤ 0.0001. Source data

**Extended Data Fig. 4:**
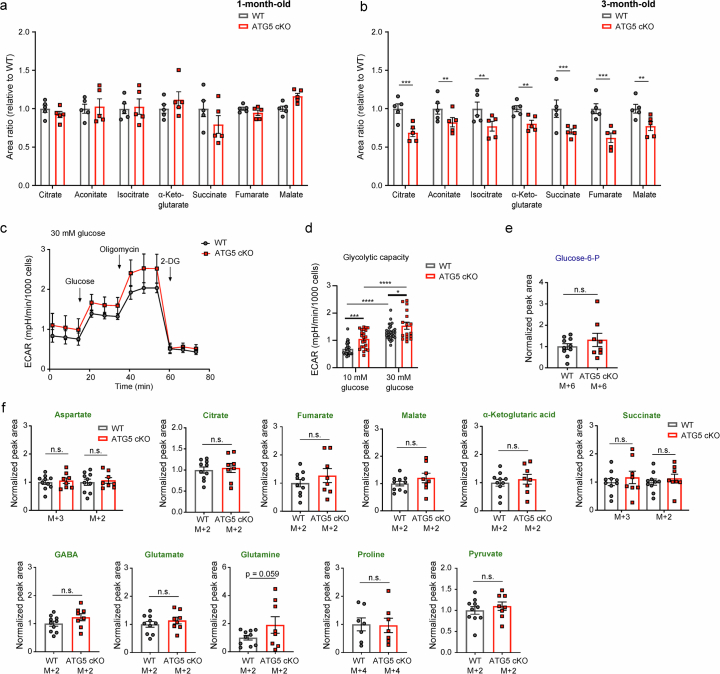
The decrease in TCA cycle intermediates is secondary to altered glycolysis in the ATG5 cKO cerebellum. **a,b**, Relative profile of TCA cycle intermediates in 1 M (a) and 3 M (b) WT and ATG5 cKO cerebellum. N = 5 per genotype. Multiple unpaired two-sided t-Tests with linear Benjamini, Krieger and Yekutieli correction. **c,d**, ECAR rates during Seahorse XF glycolysis stress test in WT and ATG5 cKO primary cerebellar cells cultured either in 10 or 30 mM glucose. WT^10 mM^: 29 wells from N = 6, ATG5 cKO^10 mM^ 22 wells from N = 5, WT^30 mM^: 29 wells from N = 6, ATG5 cKO^30 mM^ 17 wells from N = 4 with ± 20 000 cells per well. Two-way ANOVA followed by Holm-Šidák multiple comparisons (WT^10 mM^ vs WT^30 mM^: p < 0.0001; ATG5 cKO^10 mM^ vs WT^10mM^: p = 0.0005, WT^30 mM^ vs ATG5 cKO^30 mM^: p = 0.024, ATG5 cKO^30 mM^ vs ATG5 cKO^10mM^: p < 0.0001). **e**, Enrichment of ^13^C-glucose in the glucose-6-P M + 6 fraction in acute cerebellar WT and ATG5 cKO slices at 3 M (N = 10 for WT, N = 8 for ATG5 cKO mice). cKO values are normalized to WT. One-tailed unpaired t-Test. **f**, Analysis of the metabolic flux of ^13^C-glucose into TCA cycle intermediates in acute cerebellar WT and ATG5 cKO slices at 3 M. N = 10 mice for WT, N = 8 for ATG5 cKO. cKO values are normalized to WT. One-tailed unpaired t-Test. 1 M and 3 M indicate 1 and 3 months of age, respectively. All graphs show mean ± SEM. n.s.—non-significant; * indicates P ≤ 0.05; ** indicates P ≤ 0.01; *** indicates P ≤ 0.001; **** indicates P ≤ 0.0001. Source data

To characterize the glycolytic changes in the cerebellum of ATG5 cKO mice, we conducted targeted liquid chromatography–tandem mass spectrometry (LC–MS/MS) profiling of glucose metabolism intermediates. By 1 month, the ATG5 cKO cerebellum already exhibited a significant increase in glycolytic intermediates, along with intermediates from the PPP and the glycoconjugate synthesis pathways, including sedoheptulose-7-phosphate and mannose-6-phosphate (Fig. 3a and Supplementary Data 4). The increase in fructose-6-phosphate and glucose-6-phosphate intensified further in 3-month-old cerebellum (Fig. 3b). In agreement with unchanged mitochondrial respiration (Extended Data Fig. 2i–o), we detected no alterations in the tricarboxylic acid (TCA) cycle in the 1-month-old ATG5 cKO cerebellum (Extended Data Fig. 4a). However, a small but significant decrease in TCA intermediates was noted in 3-month-old ATG5 cKO cerebellum (Extended Data Fig. 4b and Supplementary Data 4), suggesting that elevated glycolysis at 1 month might suppress mitochondrial respiration later, paralleling similar patterns observed in proliferating cells and brain astrocytes^40,41^.

Next, we assessed the glycolytic capacity of WT and ATG5-deficient cerebellar neurons cultured in 10 mM glucose, using the Seahorse Metabolic Analyzer to measure extracellular acidification rate (ECAR). ATG5 cKO neurons showed a marked increase in glycolytic function compared to WT (Fig. 3c–f). Glycolytic capacity was further upregulated in ATG5 cKO neurons cultured in 30 mM glucose (Extended Data Fig. 4c,d), suggesting that higher glucose availability facilitates glycolytic metabolism in autophagy-deficient neurons. Finally, we also evaluated the impact of ATG5 deficiency on glucose flux in acute cerebellar slices by the ^13^C-labelling technique using [^13^C]glucose as a tracer. We found a significant increase in total ^13^C incorporation in the majority of glycolytic and PPP intermediates (including glucose, lactate, pentose-5-phosphate and sedoheptulose-7-phosphate) as well as in serine, glycine and alanine in cKO slices (Fig. 3g and Extended Data Fig. 4e). This indicates an overall increase in glycolytic flux in the autophagy-deficient cerebellum. Notably, glucose flux into the TCA cycle remained similar between WT and ATG5 cKO cerebellar slices (Extended Data Fig. 4f).

**Fig. 4:**
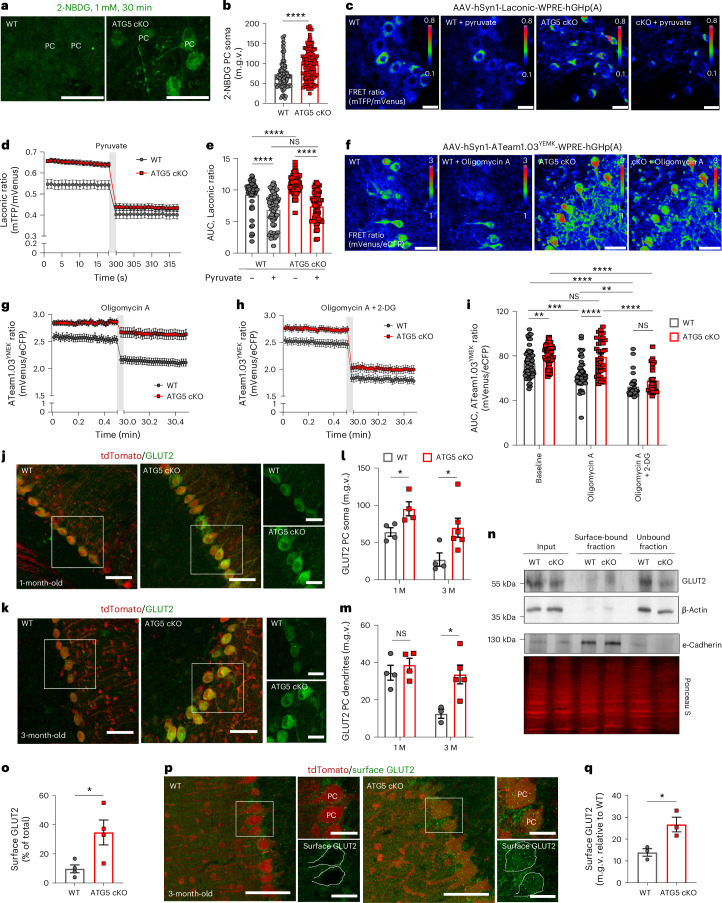
Alterations in glycolysis in ATG5 cKO PCs correlate with their elevated GLUT2 levels. **a**,**b**, Representative confocal images (**a**) of 2-NBDG uptake in WT (108 cells) and ATG5 cKO (139 cells) PCs and its fluorescence-based analysis (**b**). Scale bars, 50 µm. *N* = 4 mice. *P* < 0.0001. **c**–**e**, FRET (**c**), ratiometric analysis (**d**) and area under the curve (AUC) (**e**) of Laconic in PCs from WT and ATG5 cKO OTCs, perfused with 5 µM pyruvate for 5 min. Baseline: WT *N* = 3 (51 cells), cKO *N* = 4 (84 cells); pyruvate: WT *N* = 3 (59 cells), cKO *N* = 4 (70 cells). Scale bars, 20 µm. WT^baseline^ versus cKO ^baseline^: *P* < 0.0001; WT^baseline^ versus WT^pyruvate^: *P* < 0.0001, cKO^baseline^ versus cKO^pyruvate^: *P* < 0.0001. **f**,**g**, FRET (**f**) and ratiometric (**g**) analysis of ATeam1.03^YEMK^ in PCs from WT and ATG5 cKO OTCs, perfused with 1.5 µM oligomycin A for 30 min. WT *N* = 5 (46 cells), cKO *N* = 5 (35 cells). Scale bars, 50 µm. **h**, Ratiometric analysis of ATeam1.03^YEMK^ in WT and ATG5 cKO PCs (30 cells, *N* = 5 per genotype) after perfusion with 1.5 µM oligomycin A and 10 mM 2-DG for 30 min. **i**, AUC of ATeam1.03^YEMK^ in WT and ATG5 cKO PCs. mTFP, monomeric teal fluorescent protein; mVenus, monomeric Venus protein; eCFP, enhanced cyan fluorescent protein. Baseline: WT 76 cells *N* = 10, cKO 70 cells *N* = 10; oligomycin A: WT 46 cells *N* = 5, cKO 35 cells *N* = 5; oligomycin A + 2-DG: WT 30 cells *N* = 5, cKO 35 cells *N* = 5. WT^Baseline^ versus WT^OligomycinA+2-DG^: *P* < 0.0001; WT^Baseline^ versus cKO^Baseline^: *P* < 0.0029; cKO^Baseline^ versus cKO^OligomycinA+2-DG^, *P* < 0.0001; WT^Baseline^ versus WT^OligomycinA^: *P* = 0.004; WT^OligomycinA^ versus cKO^OligomycinA^: *P* < 0.0001; cKO^Baseline^ versus cKO^OligomycinA+2-DG^, *P* < 0.0001. **j**–**m**, GLUT2 levels in WT and ATG5 cKO PCs at 1 M (**j**) and 3 M (**k**). Scale bars, 50 µm; magnified image scale bars, 10 µm. *N* = 4 for 1 M per genotype and 3 M WTs, *N* = 6 for 3 M ATG5 cKO soma and *N* = 5 for dendrites. **l**, Quantitative analysis of GLUT2 levels in PC soma; WT^1M^ versus cKO^1M^: *P* = 0.031, WT^3M^ versus cKO^3M^, *P* = 0.022. **m**, Quantitative analysis of GLUT2 levels in PC dendrites; WT^3M^ versus cKO^3M^: *P* = 0.022. **n**,**o**, Western blot (**n**) and analysis (**o**) of GLUT2 surface biotinylation in 3 M WT and ATG5 cKO cerebellum. *N* = 4 mice per genotype, *P* = 0.032. Western blot of E-cadherin shows pooled WT and ATG5 cKO samples and is not used for quantification in **o** or directly comparable to the GLUT2 and β-actin western blot shown above. **p**, GLUT2 surface levels in WT and ATG5 cKO PCs. White circles outline PCs. Scale bars, 50 µm; magnified image scale bars, 10 µm. **q**, Quantitative analysis of images in **p**. *N* = 3 mice per genotype, *P* = 0.027. m.g.v., mean grey value. Squares in **j**, **k** and **p** indicate regions magnified. A coloured bar in **c** and **f** indicates the relative FRET signal. Statistical significance in **e**, **i** and **m** was determined by two-way ANOVA followed by Holm–Sidak multiple-comparisons test. Statistical significance in **b**, **o** and **q** was determined by two-tailed unpaired *t*-test. All graphs show the mean ± s.e.m. *P ≤ 0.05; **P ≤ 0.01; ***P ≤ 0.001; ****P ≤ 0.0001. Source data

**Extended Data Fig. 5:**
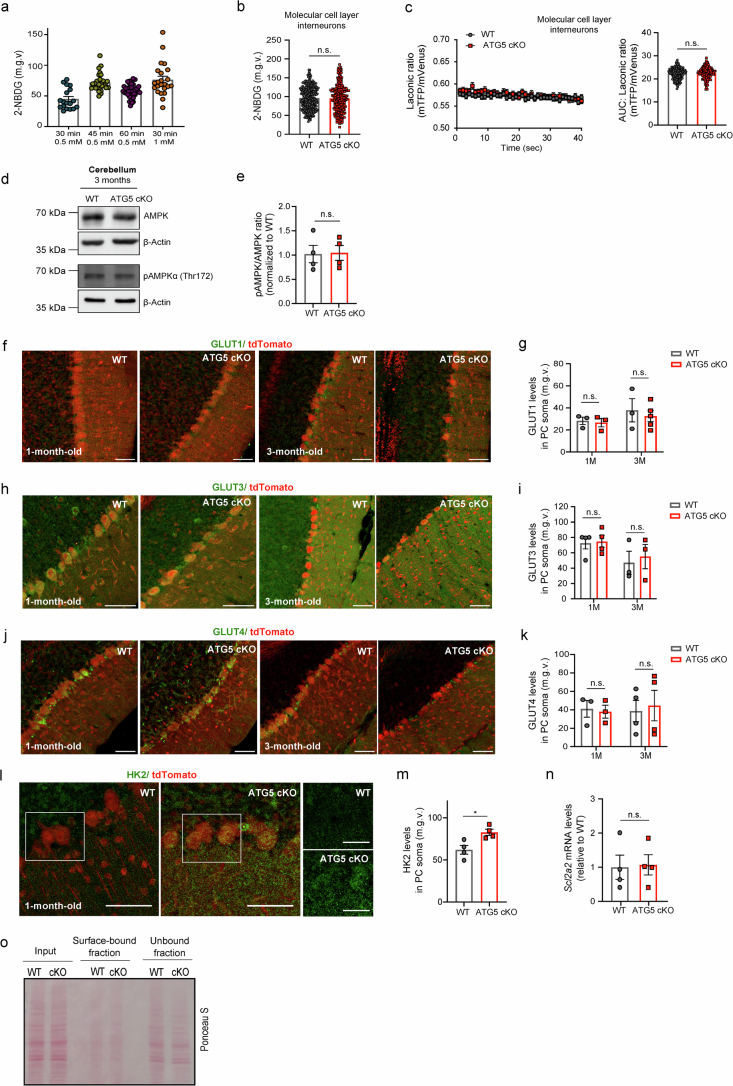
pAMPK levels and GLUT1/3/4 levels in the ATG5 cKO cerebellum. **a**, 2-NBDG uptake in PCs from WT OTCs from 1 independent experiment. **b**, 2-NBDG uptake in molecular cell layer interneurons in WT (290 cells from N = 6) and ATG5 cKO (242 cells from N = 4) OTCs. Two-tailed unpaired t-Test. **c**, Ratiometric analysis of Laconic and AUC in molecular cell layer interneurons in WT (158 cells from N = 5) and ATG5 cKO (134 cells from N = 4) OTCs at DIV21. Two-tailed unpaired t-Test. **d,e**, Protein levels of AMPK and its phosphorylated form (pAMPKα-Thr172) in cerebellar WT and ATG5 cKO lysates at 3 M. N = 4 per genotype. Two-tailed unpaired t-Test. **f-k**, Representative confocal images and analysis of WT and ATG5 cKO cerebellum, immunostained for GLUT1 (N = 3 for 1 M per genotype and 3 M WT and N = 5 for 3 M ATG5 cKO animals), GLUT3 (N = 4 per genotype for 1 M and N = 3 for 3 M) and GLUT4 (N = 3 for 1 M and N = 4 for 3 M animals per genotype). Scale bar, 50 µm. Two-way ANOVA followed by Holm-Šidák multiple comparisons. **l,m**, Fluorescence analysis of hexokinase II (HK2) in PC soma of WT and ATG5 cKO cerebellum at 1 M. Scale bar, 50 µm (Inserts, 10 µm). N = 4 mice per genotype. Two-tailed unpaired t-Test: p = 0.017. **n**, *Slc2a2* mRNA levels in WT and ATG5 cKO cerebellum at 3 M. mRNA levels of each genotype were normalized to *Gapdh*. N = 4 mice per genotype. One-tailed unpaired t-Test. **o**, Ponceau S-stained membrane used for Western Blot shown in Fig. 4n (representative image from N = 4 independent experiments). 1 M and 3 M indicate 1 and 3 months, respectively. m.g.v.-mean gray value. All graphs show mean ± SEM. n.s.—non-significant; * indicates P ≤ 0.05; ** indicates P ≤ 0.01; *** indicates P ≤ 0.001; **** indicates P ≤ 0.0001. Source data

The data above indicate that ATG5 loss increases glucose metabolism in the cerebellum. However, they do not yet clarify if observed glycolysis and/or glucose flux changes in cultured cerebellar neurons (via Seahorse assay) and cerebellar slices (using [^13^C]glucose) are specific to PCs or involve other cell types, like granule neurons and glia. To determine whether autophagy-deficient PCs take up more glucose, we utilized the fluorescent glucose substrate 2-NBDG and measured its uptake in PCs ex vivo using cerebellar organotypic slice culture (OTC; Extended Data Fig. 5a). The results demonstrated a significant facilitation of glucose uptake in ATG5-deficient PCs (Fig. 4a,b), but not in molecular layer interneurons (Extended Data Fig. 5b). Concomitant with this enhanced glucose uptake, autophagy-deficient PCs exhibited elevated lactate production, which was measured with the lactate FRET sensor ‘Laconic’^42^ (Fig. 4c–e). This rise in lactate was not due to pH difference between WT and ATG5 cKO PCs, as application of 5 mM pyruvate—a treatment known to reduce intracellular lactate by trans-accelerating lactate transporters^43^—normalized the Laconic ratio between WT and ATG5 cKO condition (Fig. 4d,e). Importantly, this phenotype was specific to PCs, as ATG5-deficient molecular layer interneurons did not show differences in lactate production compared to controls (Extended Data Fig. 5c).

To determine whether the elevated lactate levels in ATG5 cKO PCs result from increased ATP production via aerobic glycolysis, we measured cytoplasmic ATP in WT and ATG5 cKO PCs using the genetically encoded ATeam1.03^YEMK^ FRET sensor under control conditions and conditions when mitochondrial ATP production was inhibited. Cytoplasmic ATP levels were significantly higher in ATG5 cKO PCs compared to controls (Fig. 4f–h) and remained elevated even after inhibiting mitochondrial respiration with oligomycin A (Fig. 4i). Because the persistent ATP levels in ATG5 cKO PCs (Fig. 4g) suggest a substantial contribution from glycolysis, we next perfused cerebellar OTCs with a ‘cocktail’ of oligomycin A and 2-deoxy-d-glucose (2-DG), which blocks both glycolysis and oxidative phosphorylation, thus preventing any cellular ATP synthesis. As expected, this treatment significantly reduced ATP levels in ATG5 cKO PCs (Fig. 4h,i). Notably, when both glycolysis and oxidative phosphorylation were inhibited, ATP levels were comparable between WT and ATG5 cKO PCs (Fig. 4i). Consistent with the elevated ATP production in autophagy-deficient PCs, we observed no reduction in phosphorylated AMPK levels in cerebellar ATG5 cKO lysates (Extended Data Fig. 5d,e), further supporting the hypothesis of increased ATP generation through glycolysis in ATG5 cKO PCs.

### Loss of ATG5 increases GLUT2 levels in PCs

We next explored how autophagy loss influences cerebellar glucose metabolism. Enhanced glucose uptake and glycolysis have been reported in cells overexpressing various members of the *Slc2* family of glucose transporters (GLUT)^44–47^. Among the 14 mammalian GLUT transporters, GLUT1–GLUT4 are expressed in the brain^48^. Thus, we analysed the protein levels of GLUT1–GLUT4 in PCs in 1-month-old and 3-month-old WT and ATG5 cKO mice by IHC. Levels of GLUT1, GLUT3 and GLUT4 were unaltered in ATG5 cKO PCs (Extended Data Fig. 5f–k). In contrast, GLUT2 levels were significantly increased in PCs lacking ATG5, and this increase was already evident at 1 month of age (Fig. 4j–m). The accumulation of GLUT2 was initially observed at the cell soma and was subsequently evident in the dendrites of 3-month-old ATG5 cKO PCs (Fig. 4m). Consistent with increased glycolysis and elevated GLUT2 levels in the 1-month-old cerebellum, we also found that the levels of hexokinase 2 were also significantly upregulated in 1-month-old PCs in ATG5 cKO mice (Extended Data Fig. 5l,m). The increase in GLUT2 protein levels was not due to a change in mRNA levels (Extended Data Fig. 5n). Because glucose uptake typically involves the translocation of GLUT2 from an intracellular pool to the plasma membrane^49^, we hypothesized that a portion of the upregulated GLUT2 is localized at the plasma membrane in ATG5 cKO mice. Indeed, we detected an increase in surface GLUT2 levels in the ATG5 cKO cerebellum (Fig. 4n,o; see also Extended Data Fig. 5o for protein stain). As cerebellar lysates contain various cell types, including glial and granule cells, which could attenuate the effect of ATG5 cKO on GLUT2 surface levels, we conducted a targeted analysis of GLUT2 surface levels in WT and ATG5 cKO PCs using an N-terminal-specific GLUT2 antibody (Fig. 4p). This analysis confirmed a significant upregulation of surface GLUT2 levels in PCs lacking ATG5 (Fig. 4q). These findings suggest that the increased GLUT2 protein levels may contribute to the excessive glycolysis observed in PCs lacking ATG5.

### GLUT2 is degraded by the autophagy–endolysosomal system in PCs

**Extended Data Fig. 6:**
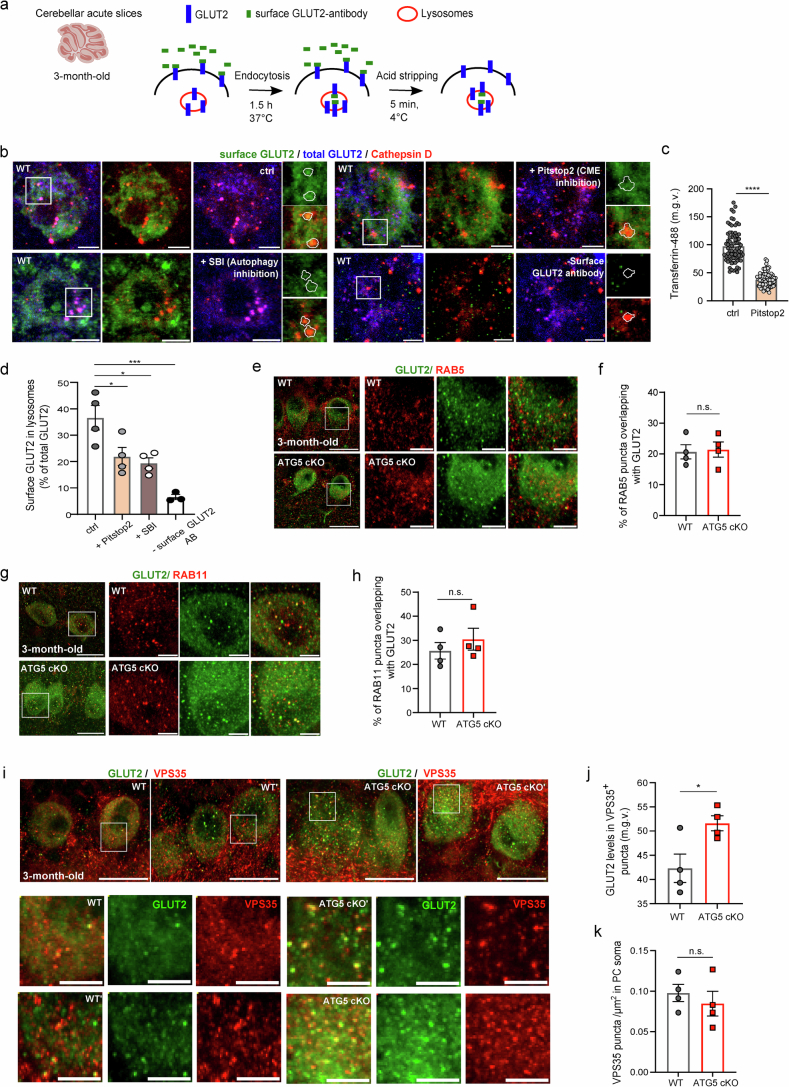
GLUT2 trafficking dynamics in the ATG5 cKO cerebellum. **a**, Schematic representation of surface GLUT2 antibody uptake in acute cerebellar slices at 3 M. Created with BioRender.com. **b**, Representative confocal images of PCs at 3 M, immunostained for surface GLUT2, total GLUT2, and Cathepsin D. Scale bar, 5 µm. N = 4 mice for control, Pitstop2- and SBI-treated slices and N = 3 mice without surface GLUT2. **c**, Transferrin-488 uptake in PCs from WT acute cerebellar slices at 3 M pretreated either with vehicle or Pitstop2 (50 µM, 1 h). Scale bar, 10 µm. 76 PCs for control and 75 PCs for Pitstop2 from N = 4 mice. Two-tailed unpaired t-Test: p < 0.0001. **d**, Surface GLUT2 levels in lysosomes from WT PCs pretreated with vehicle, Pitstop2 (50 µM, 1 h) or SBI-0206965 (500 nM, 1 h). Surface GLUT2 levels were normalized to total GLUT2 levels. N = 4 mice for control, Pitstop2- and SBI-treated slices, and N = 3 mice without surface GLUT2 antibody. One-way ANOVA followed by Holm-Šidák multiple comparisons (control vs Pitstop2: p = 0.031, control vs SBI: p = 0.015, control vs negative control, p = 0.0004). **e,f**, WT and ATG5 cKO cerebellum, immunostained for GLUT2 and RAB5 (e) and the percentage of RAB5 puncta overlapping with GLUT2 in PCs (f) at 3 M. Scale bar, 20 µm (Inserts, 5 µm). N = 4 mice per genotype. Two-tailed unpaired t-Test. **g,h**, WT and ATG5 cKO cerebellum, immunostained for GLUT2 and RAB11 (g) and the percentage of RAB11 puncta overlapping with GLUT2 in PCs (h) at 3 M. Scale bar, 20 µm (Inserts, 5 µm). N = 4 mice per genotype. Two-tailed unpaired t-Test. **i-k**, Fluorescence-based analysis of GLUT2 levels in VPS35+ endosomes and VPS35 puncta density in WT and ATG5 cKO PCs at 3 M. In (i) two examples from WT (WT and WT’) and ATG5 cKO (cKO and cKO’) cerebellum are shown. Scale bar, 20 µm (Inserts, 5 µm). N = 4 mice per genotype. Two-tailed unpaired t-Test: (j) p = 0.031. 1 M and 3 M indicate 1 and 3 months, respectively. Squares in Fig. b,e,g and i indicate regions magnified. All graphs show mean ± SEM. n.s.—non-significant; * indicates P ≤ 0.05; ** indicates P ≤ 0.01; *** indicates P ≤ 0.001; **** indicates P ≤ 0.0001. Source data

Several glycolytic pathway components undergo degradation via autophagy in non-neuronal cells^19–21,50^, while GLUTs are known to be trafficked within the endosomal system^51,52^. To clarify the exact trafficking route of internalized GLUT2 in PCs, we first conducted an ‘antibody-feeding assay’. We applied a GLUT2 luminal domain antibody to PCs ex vivo and monitored its delivery to lysosomes under basal, clathrin-mediated endocytosis (using PitStop2) and autophagy inhibition conditions (using ULK1 kinase inhibitor SBI-0206965; Extended Data Fig. 6a–c). Approximately, 35% of surface GLUT2 was delivered to lysosomes under basal conditions. Interestingly, blocking either pathway impaired lysosomal GLUT2 targeting (Extended Data Fig. 6d), suggesting two parallel intracellular pathways operate in PCs to deliver GLUT2 from plasma membrane to lysosomes. Next, we analysed the levels of GLUT2 in RAB5-marked and RAB11-marked early and recycling endosomes, finding no difference between WT and ATG5 cKO PCs, suggesting that this trafficking route is not impaired in the absence of ATG5 (Extended Data Fig. 6e–h). Given that the retromer complex regulates GLUT1 and GLUT4 trafficking from endosomes to the cell surface, and because autophagy is required for this translocation^21,53^, we speculated that GLUT2 accumulation could be due to increased GLUT2 localization in the retromer compartment. Indeed, we observed elevated levels of GLUT2 in VPS35-positive retromer compartments in ATG5 cKO PCs compared to controls (Extended Data Fig. 6i,j), while the total number of VPS35-positive puncta remained unaffected (Extended Data Fig. 6k). Our data suggest that autophagy deficiency leads to GLUT2 accumulation in retromer-associated compartments, where it may be recycled to the plasma membrane rather than being delivered to lysosomes for degradation.

**Fig. 5:**
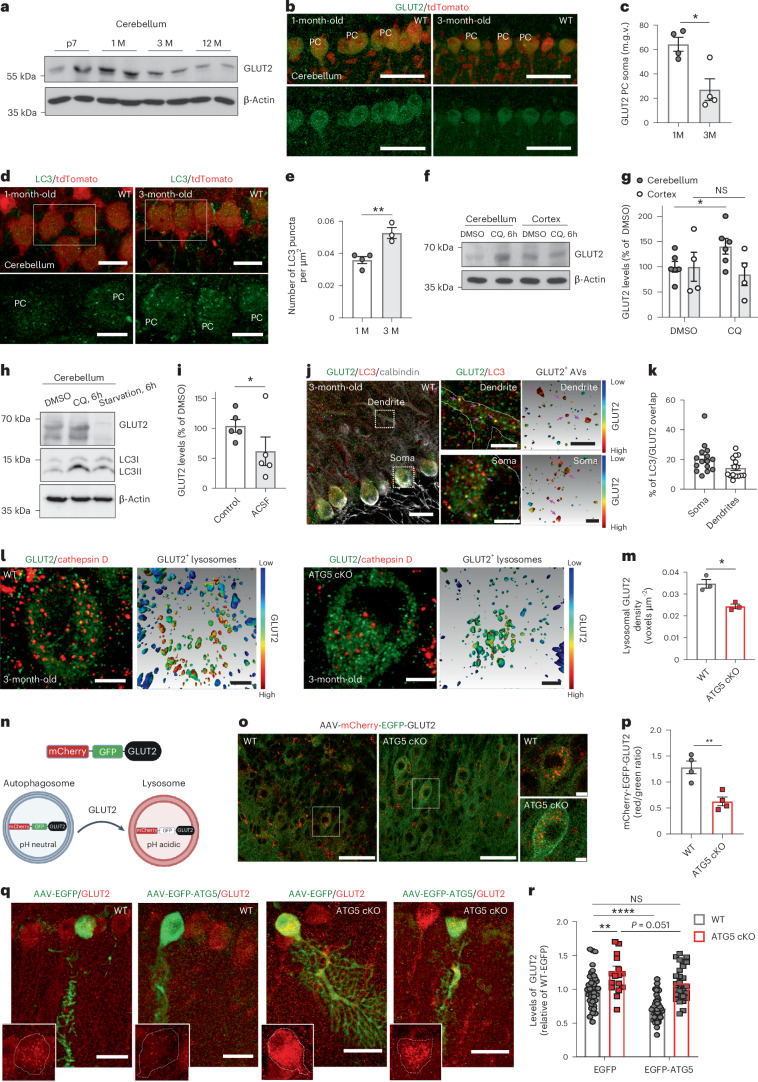
Cerebellar GLUT2 is degraded by ATG5-dependent autophagy. **a**, Immunoblot analysis of GLUT2 levels in WT cerebellum at different postnatal stages. **b**, Fluorescence-based analysis of GLUT2 levels in WT PCs. **c**, Quantitative analysis of fluorescence in **b**. *N* = 4 mice for each condition. 1 M versus 3 M: *P* = 0.012. Scale bars, 50 µm. **d**,**e**, LC3 puncta density in WT PCs. Scale bars, 20 µm; magnified image scale bars, 10 µm. *N* = 4 for 1 M and *N* = 3 for 3 M mice. 1 M versus 3 M *P* = 0.007. **f**,**g**, Immunoblot (**f**) and analysis (**g**) of GLUT2 levels in chloroquine (CQ)-treated (400 µM) cerebellar and cortical acute slices of 3 M WT animals. *N* = 6 for cerebellum and *N* = 4 for cortex. Cerebellum^Vehicle^ versus cerebellum^CQ^: *P* = 0.031. **h**,**i**, Immunoblot (**h**) and analysis (**i**) of GLUT2 protein levels in control, CQ-treated (400 µM) and ACSF-treated (6 h) cerebellar slices of 3 M WT animals. *N* = 5 per condition. Statistical significance calculated by ratio-paired two-tailed *t*-test (vehicle versus ACSF: *P* = 0.043). **j**,**k**, LC3 overlap with GLUT2 in WT PC soma and dendrites, immunostained for GLUT2, LC3 and calbindin. Right: 3D reconstructions. Pink arrows indicate GLUT2^+^ autophagosomes. Scale bars, 20 µm; magnified regions, 5 µm; 3D reconstructions, 1 µm; 15 sections from *N* = 3 mice. **l**, Left: confocal images (left) and 3D reconstructions (right) of colocalization of GLUT2 with cathepsin D in WT PCs. Right: confocal images (left) and 3D reconstructions (right) of colocalization of GLUT2 with cathepsin D in ATG5 cKO PCs. Scale bars, 5 µm; 3D reconstruction scale bars, 1 µm. **m**, Colocalization analysis of GLUT2 with cathepsin D in WT and ATG5 cKO PCs. *N* = 3 mice per genotype. WT versus cKO: *P* = 0.01. **n**, Schematic of tandem–tagged mCherry-EGFP-GLUT2. **o**, mCherry-EGFP-GLUT2 ratios in WT and ATG5 cKO PCs. Scale bars, 50 µm; magnified image scale bars, 10 µm. **p**, Quantitative analysis of data in **o**. *N* = 4 mice per genotype. WT versus cKO *P* = 0.004. **q**, GLUT2 levels in PCs in 3 M WT and ATG5 cKO mice injected with either AAV-EGFP-ATG5 or AAV-EGFP. Scale bars, 20 µm. Insets: transduced PCs outlined by dashed circles. **r**, Quantitative analysis of data in **q**. cKO^EGFP^: 17 PCs *N* = 3; cKO^ATG5-EGFP^: 27 PCs *N* = 3; WT^ATG5-EGFP^: 47 PCs *N* = 3, WT^EGFP^: 36 PCs *N* = 5. WT^EGFP^ versus KO^EGFP^: *P* = 0.001, WT^EGFP^ versus WT^ATG5-EGFP^: *P* < 0.0001. In **j** and **l**, the colour-coded bar represents colocalization ratio, with the warm colours indicating strong colocalization. Squares in **d**, **j** and **o** indicate regions magnified. All graphs show the mean ± s.e.m. Statistical significance in **c**, **e**, **m** and **p** was determined by two-tailed unpaired *t*-test. Statistical significance in **g** and **r** was determined by two-way ANOVA followed by Holm–Sidak multiple-comparisons test. *P ≤ 0.05; **P ≤ 0.01; ***P ≤ 0.001; ****P ≤ 0.0001. Source data

**Extended Data Fig. 7:**
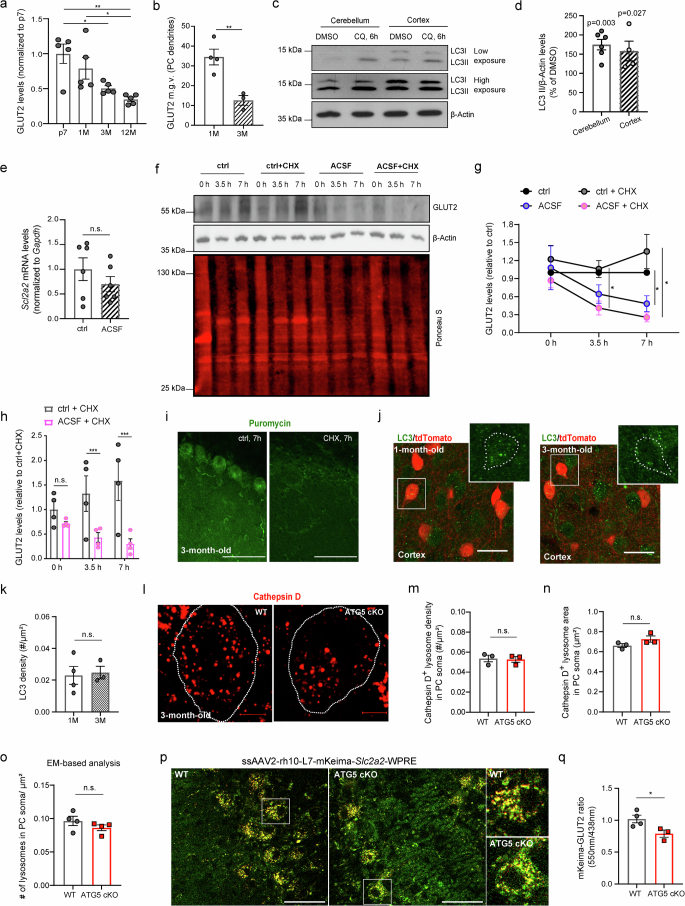
Age-related alterations in GLUT2 expression and degradation dynamics in the cerebellum. **a**, GLUT2 protein levels in WT cerebellar lysates at different postnatal stages. N = 5 mice per condition. One-way ANOVA followed by Holm-Šidák multiple comparisons (p7 vs 12 M: p = 0.034, p7 vs 3 M: p = 0.024, p7 vs 12 M: p = 0.003). **b**, GLUT2 protein levels in PC dendrites. N = 4 mice for 1 M and N = 3 mice for 3 M. Two-tailed unpaired t-Test: p = 0.007. **c,d**, LC3 protein levels in DMSO- and CQ- treated cerebellar and cortical acute slices of WT animals at 3 M. LC3 II levels in (d) were normalized to DMSO- treated acute slices (set to 100%). N = 6 mice for cerebellum and N = 4 for cortex. Two-way ANOVA followed by Holm-Šidák multiple comparisons (cerebellum^vehicle^ vs cerebellum^CQ^: p = 0.003, cortex^vehicle^ vs cortex^CQ^: p = 0.027). **e**, *Slc2a2* mRNA levels in 3 M WT cerebellar acute slices after 6 h in ACSF. mRNA levels were normalized to *Gapdh*. N = 6 mice per condition. One-tailed unpaired t-Test. **f-h**, GLUT2 levels in 3 M WT cerebellar slices after 0 h, 3.5 h and 7 h of incubation in control medium, CHX (60 µM), ACSF or ACSF + CHX. GLUT2 levels were normalized to 0 h. N = 4 for each condition and timepoint. For (g) two-way ANOVA followed by Two-stage linear step-up procedure of Benjamini, Krieger and Yekutieli (control ^CHX 3.5h^ vs ACSF^CHX 3.5h^: p = 0.032; control^CHX 7h^ vs ACSF^CHX 7h^: p = 0.011, ACSF^7h^ vs control^7h^: p = 0.015). For (h) GLUT2 levels were normalized to control^CHX 0h^ followed by two-way ANOVA followed by Holm-Šidák multiple comparisons (ACSF^CHX 3,5h^ vs control^CHX 3.5h^: p = 0.0008, ACSF^CHX 7h^ vs control ^CHX 7h^: p = 0.0002). **i**, Example confocal images of cerebellar slices from WT animals at 3 M, immunostained for Puromycin from N = 1 independent experiment. Scale bar, 50 µm. **j,k**, LC3 puncta density in cortical WT interneurons. Scale bar, 50 µm. N = 4 mice for 1 M and N = 3 for 3 M. Two-tailed unpaired t-Test. **l-o**, Fluorescence-based (l-n) and EM-based (o) analyses of lysosomal density and area in WT and ATG5 cKO PCs at 3 M. Scale bar, 50 µm. Image in (l) represents Cathepsin channel shown in Fig. 5l. N = 4 mice per genotype. Two-tailed unpaired t-Test. **p,q**, Analysis of mKeima -GLUT2 in PCs from WT and ATG5 cKO OTCs. Scale bar, 50 µm. N = 4 WT and N = 3 ATG5 cKO mice. Two-tailed unpaired t-Test: p = 0.041. 1 M and 3 M indicate 1 and 3 months, respectively. Squares in Fig. S7j,p indicate regions magnified. All graphs show mean ± SEM. n.s.—non-significant; * indicates P ≤ 0.05; ** indicates P ≤ 0.01; *** indicates P ≤ 0.001; **** indicates P ≤ 0.0001. Source data

We next hypothesized that autophagy, mediated by ATG5, limits glycolysis by degrading cerebellar GLUT2, a function potentially critical during brain maturation when glycolytic demand decreases^54^. To test this, we analysed the levels of GLUT2 in the WT cerebellum across maturation stages. GLUT2 in cerebellum, in particular in PCs, declined from postnatal day 7 (P7) to 3 months and continued decreasing until 12 months (Fig. 5a–c and Extended Data Fig. 7a,b). This decrease was accompanied by a concomitant increase in autophagosome number in 3-month-old PCs (Fig. 5d,e) and likely resulted from autophagy-mediated degradation, as chloroquine treatment significantly increased GLUT2 levels (Fig. 5f,g). This effect was cerebellum specific, as chloroquine-treated cortical slices showed no change in GLUT2 levels despite autophagy inhibition (Fig. 5g and Extended Data Fig. 7c,d). Conversely, GLUT2 levels significantly decreased in cerebellar slices subjected to starvation with artificial cerebrospinal fluid (ACSF; Fig. 5h,i). The reduction in GLUT2 levels after starvation was not due to its decreased mRNA levels (Extended Data Fig. 7e). Furthermore, in a cycloheximide (CHX) chase assay, we observed that blocking protein translation significantly accelerated GLUT2 degradation during starvation (Extended Data Fig. 7f–i), supporting that starvation-induced reduction in GLUT2 levels is independent of protein transcription, and/or translation and is instead driven by its degradation. Notably, the ratio of LC3-II to LC3-I, used as a read-out of autophagy flux after chloroquine treatment, was slightly higher in the cerebellar lysates, suggesting a higher level of constitutive autophagy in the cerebellum compared to the cortex (Extended Data Fig. 7d). In line with this, the number of LC3 puncta in the cortex was low and remained unaltered between the ages of 1 month and 3 months (Extended Data Fig. 7j,k).

To investigate whether GLUT2 is trafficked within autophagosomes in PCs, we analysed its colocalization with LC3 in 3-month-old PCs using three-dimensional (3D) reconstructions (Fig. 5j). Approximately 20% of LC3-positive autophagosomes contained GLUT2 (Fig. 5k). In line with prior findings in non-neuronal cells^51^, GLUT2 was abundant in WT lysosomes, whereas its lysosomal density was markedly reduced in ATG5 cKO PCs (Fig. 5l,m). The overall lysosome number and area remained unaltered in PCs lacking ATG5 (Extended Data Fig. 7l–o). To determine whether the accumulation of GLUT2 in retromer-positive compartments in ATG5 cKO PCs (Extended Data Fig. 6i,j) impacts its lysosomal degradation, we utilized a tandem mCherry-EGFP-tagged GLUT2 construct (Fig. 5n). Loss of ATG5 reduced lysosomal GLUT2 levels in PCs overexpressing mCherry-EGFP-GLUT2 (Fig. 5o,p). Further, using an adeno-associated virus (AAV) construct expressing mKeima-tagged GLUT2 under the PC-specific L7 promoter, we confirmed a marked reduction in lysosomal GLUT2 in ATG5 cKO PCs compared to WT (Extended Data Fig. 7p,q).

Overexpression of ATG5 was previously reported to activate autophagy in vivo^55^. Thus, we hypothesized that if GLUT2 is degraded by autophagy, overexpression of ATG5 should restore its levels in autophagy-deficient PCs. Supporting this, WT PCs overexpressing EGFP-ATG5 displayed significantly downregulated GLUT2, while re-expressing ATG5 in cKO PCs restored GLUT2 to near-normal levels (Fig. 5q,r). Taken together, our data demonstrate that GLUT2 undergoes degradation by the autophagy–endolysosomal system in PCs in vivo.

### Glycolytic by-products LPA and serine increase PC vulnerability

**Fig. 6:**
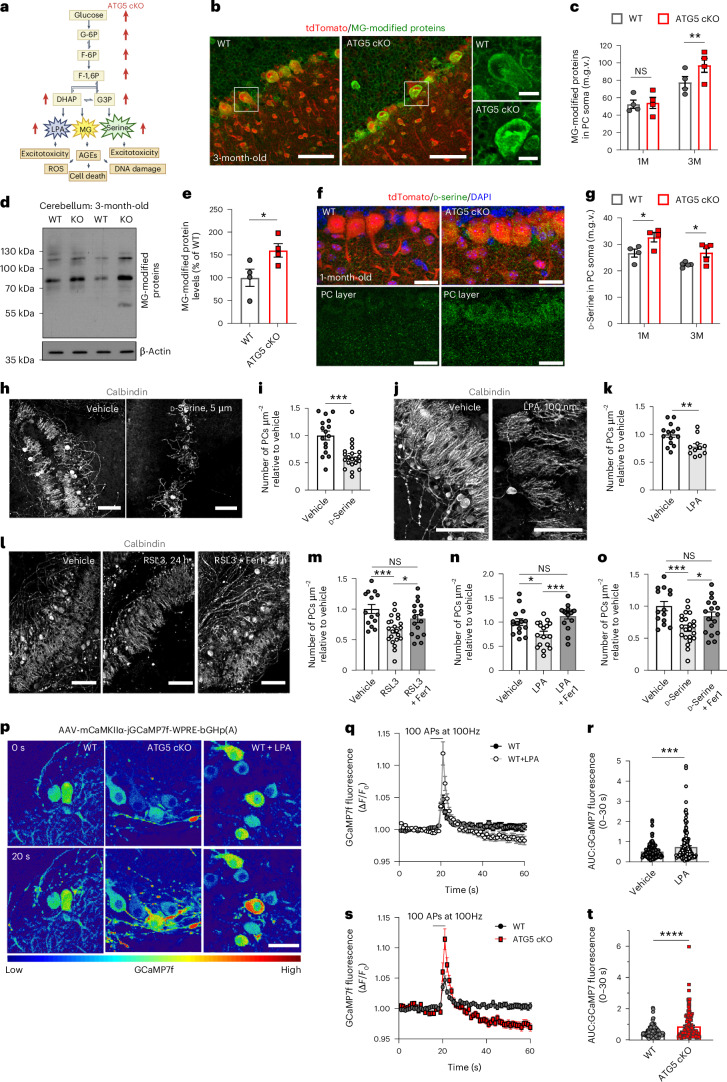
By-products of aerobic glycolysis cause neurodegeneration of PCs. **a**, Schematic illustrating upregulated glycolytic by-products in ATG5 cKO cerebellum. AGEs, advanced glycation end products; ROS, reactive oxygen species. **b**, Fluorescence-based analysis of MG-modified proteins in WT and ATG5 cKO PCs. Scale bars, 50 µm; magnified image scale bars, 10 µm. **c**, Quantitative analysis of data in **b**. *N* = 4 mice per genotype. Statistical significance calculated by two-way ANOVA followed by Holm–Sidak multiple-comparisons test (WT^3M^ versus cKO^3M^, *P* = 0.008). **d**,**e**, Immunoblot (**d**) and analysis (**e**) of MG-modified proteins in WT and ATG5 cerebellum at 3 M. *N* = 4 mice per genotype. *P* = 0.046. **f**, Fluorescence-based analysis of levels of d-serine in WT and ATG5 cKO PCs. Scale bars, 20 µm. **g**, Quantitative analysis of data in **f**. *N* = 4 for 1 M and *N* = 5 for 3 M mice. Statistical significance calculated by two-way ANOVA followed by Holm–Sidak multiple-comparisons test (WT^1M^ versus cKO^1M^: *P* = 0.025, WT^3M^ versus cKO^3M^: *P* = 0.031). **h**, Fluorescence-based analysis of PC density in OTCs cultured in control media (vehicle) or 5 µM d-serine. Scale bars, 100 µm. **i**, Quantitative analysis of data in **h**; 16 images for control, 21 images for d-serine, *N* = 3. *P* = 0.0003. **j**, Fluorescence-based analysis of PC density in OTCs treated with either vehicle or 100 nM LPA. Scale bars, 100 µm. **k**, Quantitative analysis of data in **j**; 14 images for control, 11 images for LPA, *N* = 3. *P* = 0.007. **l**, Fluorescence-based analysis of PC density in OTCs treated for 24 h with vehicle, RSL3 (1 µM) or RSL3 + Fer1 (10 µM). Scale bars, 100 µm. **m**, Quantitative analysis of data in **l**; 28 images for control, 30 images for RSL3, 25 images for RSL3 + Fer1, *N* = 4. Vehicle versus RSL3: *P* = 0.0007; RSL3 versus RSL3 + Fer1: *P* = 0.037. **n**, PC density in OTCs treated with vehicle, LPA (100 nM) or LPA + Fer1 (5 µM). 15 images for control, 17 images for LPA and 17 images for LPA + Fer1, *N* = 3. Vehicle versus LPA: *P* = 0.01, LPA versus LPA + Fer1: *P* = 0.0003. **o**, PC density in OTCs cultured in control media containing vehicle, d-Serine (10 µM) or d-serine + Fer1 (5 µM); 14 images for control, 22 images for d-serine, 17 images for d-serine + Fer1, *N* = 4. Vehicle versus d-serine: *P* = 0.0006, d-serine versus d-serine + Fer1: *P* = 0.045. **p**, GCaMP7f-based time-lapse imaging of WT, WT^LPA^ (100 nM, 7 days; **q** and **r**), and ATG5 cKO (**s** and **t**) PCs at baseline (0 seconds) and after stimulation with 100 action potentials (APs) at 100 Hz (20 seconds). Scale bar, 50 µm. **q**–**t**, Quantitative analysis of data in **p**; 164 cells for WT, 166 cells for WT^LPA^, 142 cells for cKO, *N* = 3. WT^Untreated^ versus WT^LPA^: *P* = 0.0002; WT^Untreated^ versus cKO: *P* < 0.0001. Squares in Fig. **b** indicate regions magnified. All graphs show the mean ± s.e.m. Statistical significance in **e**, **i**, **k**, **r** and **t** was determined by two-tailed unpaired *t*-test. Statistical significance in **m**–**o** was determined by one-way ANOVA followed by Holm–Sidak multiple-comparisons test. *P ≤ 0.05; **P ≤ 0.01; ***P ≤ 0.001; ****P ≤ 0.0001. Source data

**Extended Data Fig. 8:**
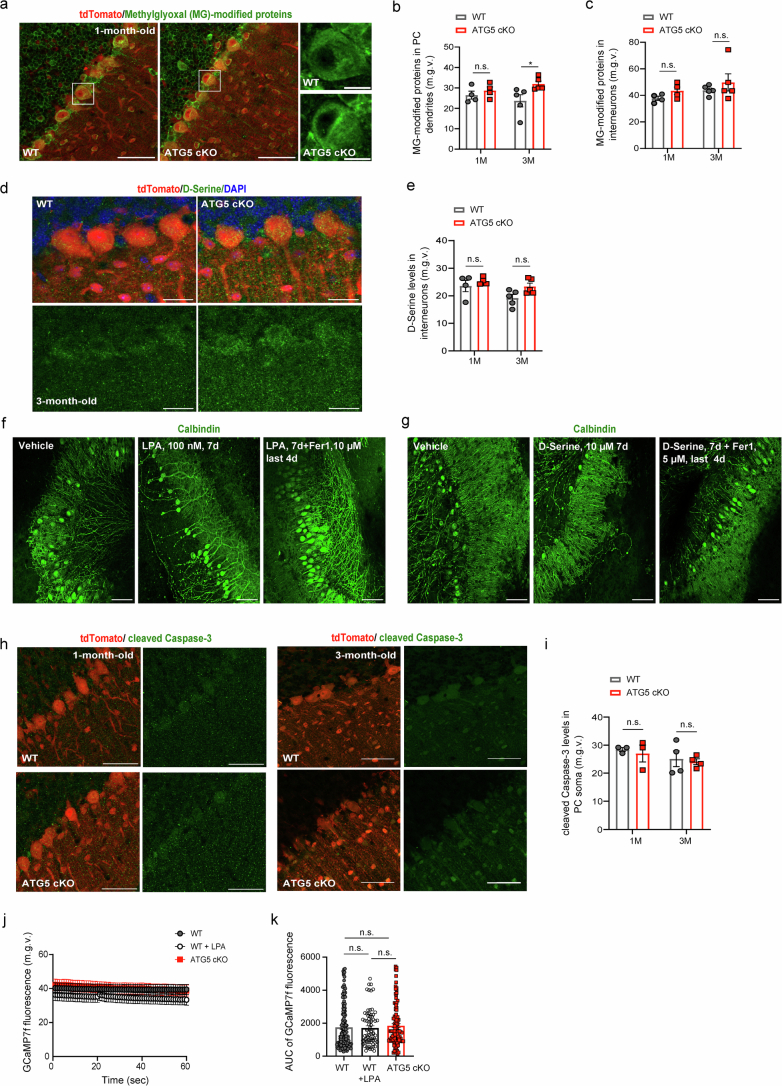
GABAergic ATG5 deletion does not induce apoptosis or alter MG-modified protein levels in cerebellar interneurons. **a-c**, Fluorescence-based analysis of MG-modified proteins in PC dendrites (b) and molecular cell layer interneurons (c) in WT and ATG5 cKO mice. Scale bar, 50 µm (Inserts, 10 µm). N = 4 for 1 M and N = 5 for 3 M animals. Two-way ANOVA followed by Holm-Šidák multiple comparisons. In b WT^3M^ vs cKO^3M^: p = 0.027. **d,e**, Fluorescence-based analysis of D-Serine in molecular cell layer interneurons from WT and ATG5 cKO mice. Scale bar, 25 µm. N = 4 mice for 1 M and N = 5 for 3 M. Two-way ANOVA followed by Holm-Šidák multiple comparisons. **f,g**, Representative confocal images of WT OTCs cultured in media containing either vehicle, LPA or LPA+Fer1. Scale bar, 100 µm. See quantification in Fig. 6n. 15 images for control, 17 images for LPA and 17 images for LPA + Fer1, from N = 3 independent experiments. **g**, Representative confocal images of WT OTCs cultured in media containing either vehicle, D-Serine or D-Serine+Fer1. Scale bar, 100 µm. See quantification in Fig. 6o. 14 images for control, 22 images for D-Serine, 17 images for D-Serine + Fer1, from N = 4 independent experiments. **h,i**, Fluorescence-based analysis of cleaved Caspase-3 levels in PCs from WT and ATG5 cKO mice. Scale bar, 200 µm. N = 3 per genotype for 1 M and N = 4 per genotype for 3 M. Two-way ANOVA followed by Holm-Šidák multiple comparisons. **j,k**, GCAMP7f-based analysis of untreated WT PCs, LPA-treated WT PCs and ATG5 cKO PCs. (159 PCs for WT, 79 PCs for WT^LPA^ and 117 PCs for ATG5 cKO, from N = 3 per genotype). One-way ANOVA followed by Holm-Šidák multiple comparisons. 1 M and 3 M indicate 1 and 3 months, respectively. m.g.v.-mean gray value. Squares in Fig. S8a indicate regions magnified. All graphs show mean ± SEM. n.s.—non-significant; * indicates P ≤ 0.05; ** indicates P ≤ 0.01; *** indicates P ≤ 0.001; **** indicates P ≤ 0.0001. Source data

Our findings on aberrant glycolytic activity in ATG5 cKO PCs and their selective vulnerability under this condition prompted us to investigate whether alterations in glycolysis are causally implicated in the PC degeneration. Uncontrolled glycolysis can result in MG production via dihydroxyacetone phosphate (DHAP; Fig. 6a, see also Fig. 2g for DHAP upregulation). MG can react with proteins, lipids and nucleic acids, forming advanced glycation end products associated with various pathophysiological mechanisms, including neurodegeneration^56^. To test whether cKO of ATG5 leads to upregulation of MG-modified proteins in PCs, we analysed their levels using IHC. We found that the levels of MG-modified proteins were significantly increased in PCs, but not in molecular layer interneurons of 1-month-old and 3-month-old mice (Fig. 6b,c and Extended Data Fig. 8a–c). The levels of MG-modified proteins were also significantly upregulated in the whole ATG5 cKO cerebellar lysates compared to controls (Fig. 6d,e).

Beyond MG, several other biosynthesis pathways can branch off from glycolysis, including DHAP-mediated synthesis of LPA^57^ and the production of l-serine by 3-phosphoglycerate^58^. While l-serine can be racemized into d-serine primarily in astrocytes^59,60^, we observed that d-serine levels were also slightly, yet significantly, increased in ATG5 cKO PCs (Fig. 6f,g) but not in molecular layer interneurons (Extended Data Fig. 8d,e). Because both LPA and d-serine (being a precursor of glycine) can exert neurotoxic effects via augmenting neurotransmission^60,61^, and both metabolites were significantly upregulated in ATG5 cKO cerebellum (Fig. 6g; see also Fig. 2g), we investigated their role in PC survival. Treatment of WT OTCs with 5 µM d-serine and/or 100 nM LPA resulted in a substantial decrease in PC density (Fig. 6h–k). This cell loss was likely due to activation of ferroptosis since the degeneration of PCs could be triggered by treatment with the ferroptosis inducer RSL3 and prevented by their additional treatment with ferrostatin 1 (Fer1; Fig. 6l,m), which is in line with the proteome data indicating activation of the ferroptosis pathway in the ATG5 cKO cerebellum (Fig. 2e). Moreover, Fer1 application prevented PC loss induced by either LPA and/or d-serine application (Fig. 6n,o and Extended Data Fig. 8f,g). Of note, the cell death of PCs after autophagy deficiency was apoptosis independent (Extended Data Fig. 8h,i). The neurotoxic effect of LPA on PC death was accompanied by an increase in PC network excitation, assessed via calcium imaging in OTCs transduced with an AAV encoding a GCaMP7f^CamKIIα^ (CamKIIα is predominantly expressed in PCs but not granule cells or interneurons in the cerebellum; Fig. 6p). Stimulation with high-frequency bursts of action potentials (100 Hz) resulted in a significant increase in GCaMP7f signals in LPA-treated WT PCs compared to controls (Fig. 6p–r). This phenotype was also observed in ATG5 cKO PCs (Fig. 6s,t). While Ca²⁺ signals are typically used as a proxy for neuronal activity, we acknowledge the possibility that ATG5-dependent autophagy may influence Ca²⁺ channel and ion pump expression on PC membranes, potentially affecting Ca²⁺ signals beyond neuronal firing. Although we cannot entirely exclude the impact of autophagy on the distribution of Ca²⁺ channels at this point, the unchanged baseline of GCaMP7f fluorescence in both LPA-treated WT and ATG5 cKO PCs without electrical stimulation (Extended Data Fig. 8j,k) suggests that the elevated Ca²⁺ signals in response to LPA, and/or in ATG5 cKO, can at least partially be attributed to enhanced neuronal activity. In summary, our findings suggest that glycolytic by-products LPA and serine contribute to PC degeneration, and that alterations in glycolysis may be a key factor in the vulnerability of PCs observed in the context of autophagy deficiency.

### ATG5:GLUT2 cKO mice have improved PC survival and ataxic gait

**Fig. 7:**
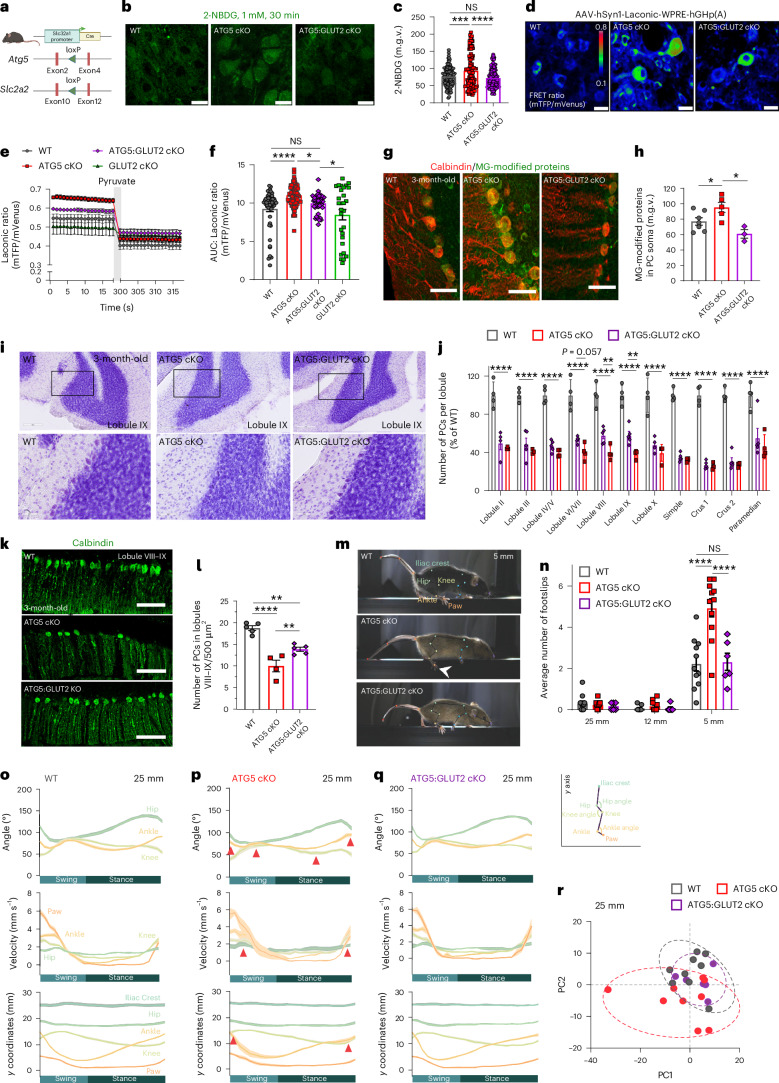
GLUT2 deletion in ATG5 cKO mice mitigates PC neurodegeneration and improves their ataxic gait. **a**, Generation of ATG5:GLUT2 cKO mice. Created with BioRender.com. **b**, 2-NBDG uptake in WT (125 cells, *N* = 5), ATG5 cKO (129 cells, *N* = 4) and ATG5:GLUT2 cKO (75 cells, *N* = 5) PCs. Scale bar, 50 µm. **c**, Quantitative analysis of data in **b**. WT versus ATG5 cKO, *P* = 0.0007; ATG5 cKO versus ATG5:GLUT2 cKO: *P* < 0.0001. **d–f**, Laconic FRET imaging (**d**) and its radiometric (**e**) and AUC analysis (**f**) in WT, ATG5 cKO and ATG5:GLUT2 cKO PCs, after 5-min perfusion with 5 mM pyruvate. WT (baseline 51 PCs, pyruvate 59 PCs from *N* = 3); ATG5 cKO (baseline 84 PCs, pyruvate 70 PCs from *N* = 4); ATG5:GLUT2 cKO (baseline 49 PCs, pyruvate 49 PCs from *N* = 3); GLUT2 cKO (baseline 29 PCs, pyruvate 29 PCs from *N* = 4). Scale bars, 20 µm. A coloured bar indicates the relative FRET signal. WT versus ATG5 cKO, *P* < 0.0001, ATG5 cKO versus ATG5:GLUT2 cKO: *P* = 0.032, ATG5:GLUT2 cKO versus GLUT2 cKO: *P* = 0.017. **g**, Levels of MG-modified proteins in PCs from WT (*N* = 7), ATG5 cKO (*N* = 5) and ATG5:GLUT2 cKO (*N* = 3) mice. Scale bars, 50 µm. **h**, Quantitative analysis of data in **g**. Statistical significance calculated by mixed-effects ANOVA followed by Holm–Sidak multiple-comparisons test. WT versus ATG5 cKO: *P* = 0.023, ATG5 cKO versus ATG5:GLUT2 cKO: *P* = 0.023. **i**, Top: Nissl-stained WT, ATG5 cKO and ATG5:GLUT2 cKO cerebellum. Scale bars, 200 µm. Bottom: magnified Nissl-stained cerebellum; scale bars, 50 µm. **j**, PC density in WT (*N* = 4), ATG5 cKO (*N* = 4) and ATG5:GLUT2 cKO (*N* = 5) cerebellum at 3 M. Statistical significance calculated by two-way ANOVA followed by Holm–Sidak multiple-comparisons test. ATG5 cKO versus ATG5:GLUT2 cKO: lobule VIII *P* = 0.006; lobule IX *P* = 0.004. WT versus ATG5 cKO, *P* < 0.0001. **k**, PC density in lobules VIII–IX in WT (*N* = 5), ATG5 cKO (*N* = 4) and ATG5:GLUT2 cKO (*N* = 5) mice. Scale bars, 200 µm. **l**, Quantitative analysis of data in **k**. WT versus ATG5 cKO: *P* < 0.0001, ATG5 cKO versus ATG5:GLUT2: *P* = 0.006, WT versus ATG5:GLUT2 cKO: *P* = 0.002. **m**, Kinematic analysis of WT, ATG5 cKO and ATG5:GLUT2 cKO mice walking on the 5-mm-wide beam. White arrowhead points to the slip in ATG5 cKO. **n**, Average number of slips in WT (*N* = 11), ATG5 cKO (*N* = 11) and ATG5:GLUT2 cKO (*N* = 6) mice at 3 M. Statistical significance calculated by two-way ANOVA followed by Holm–Sidak multiple-comparison (WT versus ATG5 cKO: *P* < 0.0001, ATG5 cKO versus ATG5:GLUT2 cKO: *P* < 0.0001). **o**–**r**, Angle, velocities and coordination of WT (*N* = 9; **o**), ATG5 cKO (*N* = 9; **p**) and ATG5:GLU T2 cKO (*N* = 6; **q**) mice walking on a 25-mm-wide beam and PCA analysis of these parameters (**r**). Red arrowheads in **p** indicate the points at which the relative positions of joints differ in ATG5 cKOs compared to WT and ATG5:GLUT2 cKOs. In **r**, individual mice are represented as coloured circles. Squares in **i** indicate regions magnified. All graphs show the mean ± s.e.m. Statistical significance in **c**, **f** and **l** was determined by one-way ANOVA followed by Holm–Sidak multiple-comparisons test. *P ≤ 0.05; **P ≤ 0.01; ***P ≤ 0.001; ****P ≤ 0.0001. Source data

**Extended Data Fig. 9:**
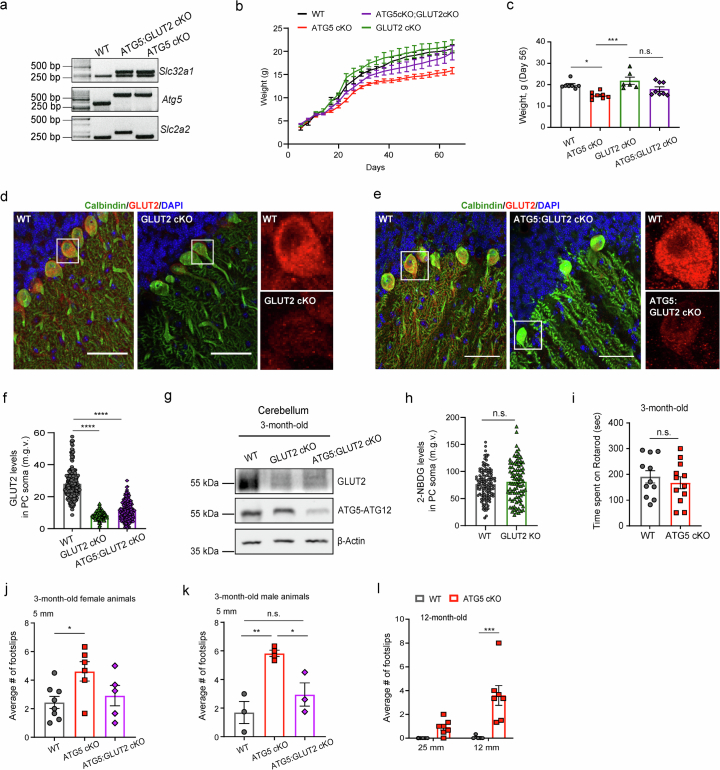
Impact of conditional ATG5:GLUT2 deletion in GABAergic neurons on bodyweight and motor coordination in mice. **a**, PCR for floxed (700 bp) and WT *Atg5* (350 bp), floxed (326 bp) and WT *Slc2a2* (235 bp). Double-floxed mice for *Atg5* and *Slc2a2* with additional hemizygosity for the Cre gene (235 bp + 326 bp) were defined as ATG5:GLUT2 cKO mice. **b,c**, Bodyweight over time (b) and at 8 weeks of age (c) of WT, ATG5 cKO, GLUT2 cKO and ATG5:GLUT2 cKO mice (N = 7 for WT, N = 8 for ATG5 cKO, N = 6 for GLUT2 cKO and N = 8 for ATG5:GLUT2 cKO mice). One-way ANOVA followed by Holm-Šidák multiple comparisons (WT vs ATG5 cKO: p = 0.011, WT vs GLUT2 cKO: p = 0.0005). **d-f**, Fluorescent analysis of GLUT2 levels in PCs of WT, GLUT2 cKO (d) and ATG5:GLUT2 cKO (e) at 3 M. Scale bar, 100 µm. N = 3 mice for WT, N = 1 for GLUT2 cKO and N = 3 for ATG5:GLUT2 cKO. One-way ANOVA followed by Holm-Šidák multiple comparisons (WT vs GLUT2 cKO: p < 0.0001, WT vs GLUT2 cKO: p < 0.0001). **g**, Immunoblot analysis of GLUT2 and ATG5 protein levels in WT, ATG5 cKO and ATG5:GLUT2 cKO cerebellar lysates from 1 independent experiment. **h**, 2-NBDG levels in WT (125 PCs from N = 5 mice) and GLUT2 cKO (85 PCs from N = 4) PCs. Two-tailed unpaired t-Test. **i**, Time spent on rotarod by WT and ATG5 cKO mice at 3 M. N = 11 for WT and N = 12 for ATG5 cKO mice. Two-tailed unpaired t-Test. **j**, Average number of slips of female WT and ATG5 cKO mice at 3 M when crossing 5 mm beam. N = 8 mice for WT, N = 6 for ATG5 cKO and N = 5 for ATG5:GLUT2 cKO. One-way ANOVA followed by Holm-Šidák multiple comparisons (WT vs ATG5 cKO: p = 0.041). **k**, Average number of slips of male WT and ATG5 cKO mice at 3 M when crossing 5 mm beam. N = 3 mice for WT, N = 4 for ATG5 cKO and N = 3 for ATG5:GLUT2 cKO. One-way ANOVA followed by Holm-Šidák multiple comparisons (WT vs ATG5 cKO: p = 0.004, ATG5 cKO vs ATG5:GLUT2 cKO: p = 0.019). **l**, Average number of slips of WT and ATG5 cKO mice at 12 M when crossing 25 mm and 12 mm beams. N = 5 mice for WT and N = 7 for ATG5 cKO. Two-way ANOVA followed by Holm-Šidák multiple comparisons (WT^12 mm^ vs ATG5 cKO^12 mm^: p = 0.0002). 1 M, 3 M and 12 M indicate 1, 3 and 12 months, respectively m.g.v.-mean gray value. Squares in Fig. S9d,e indicate regions magnified. All graphs show mean ± SEM. n.s.—non-significant; * indicates P ≤ 0.05; ** indicates P ≤ 0.01; *** indicates P ≤ 0.001; **** indicates P ≤ 0.0001. Source data

Our data described thus far suggest that the loss of autophagy stabilizes GLUT2 levels in PCs, a phenotype leading to altered glycolysis and subsequent PC death. Therefore, we asked whether reducing GLUT2 (encoded by the *Slc2a2* gene) levels could prevent PC loss in ATG5 cKO mice. Hence, we generated double KO mice with *Slc2a2* inactivation by crossing previously described *Slc2a2* floxed mice^62,63^ with the ATG5 cKO mice (Fig. 7a and Extended Data Fig. 9a). The resulting ATG5:GLUT2 cKO mice were born without abnormalities and exhibited weight gain similar to their WT littermates (Extended Data Fig. 9b). We have previously reported that ATG5 cKO mice were leaner compared to WT controls^29^. Intriguingly, the deletion of GLUT2 resulted in a modest improvement in weight gain in ATG5 cKO mice, but these changes did not reach significance (Extended Data Fig. 9c). Conversely, the sole deletion of GLUT2 in GABAergic neurons had no impact on weight regulation, consistent with prior findings^62^. We found that reducing GLUT2 protein levels (Extended Data Fig. 9d–g) prevented excessive glucose uptake in ATG5 cKO PCs (Fig. 7b,c) and markedly downregulated the production of lactate (Fig. 7d–f). Glucose uptake in PCs lacking solely GLUT2 was not impaired, suggesting potential compensation by other glucose transporters, as documented in pancreatic beta cells lacking GLUT2 (refs. ^64,65^). Although we did not directly assess the upregulation of these transporters in our study, their activity would likely prevent glucose overload in the GLUT2 cKO scenario due to their low glucose transport capacity compared to GLUT2. This contrasts with the GLUT2 overexpression observed in ATG5 cKO PCs, which drives increased glucose uptake and PC degeneration. Additionally, levels of MG-modified proteins were also significantly reduced in ATG5:GLUT2 cKO mice (Fig. 7g,h), consistent with the hypothesis that stabilized GLUT2 in autophagy-deficient PCs contributes to increased formation of glycolytic by-products such as MG.

To determine whether the rescue of glucose uptake, lactate production and MG levels in ATG5:GLUT2 cKO mice has a protective impact on PC survival, we analysed the number of PCs across various cerebellar regions in WT, ATG5 cKO and ATG5:GLUT2 cKO mice at 3 months of age. We found that the number of PCs was significantly reduced in all cerebellar lobules (in line with the analysis of calbindin-positive PCs in Fig. 1g; Fig. 7i,j). Remarkably, GLUT2 reduction improved the survival of PCs in lobules VI–IX, corresponding to the vermis region, by approximately 20% (Fig. 7j). This rescue phenotype was evident in both Nissl-stained cerebellum and cerebellar sections analysed by IHC using the calbindin antibody as a selective PC marker (Fig. 7k,l).

**Extended Data Fig. 10:**
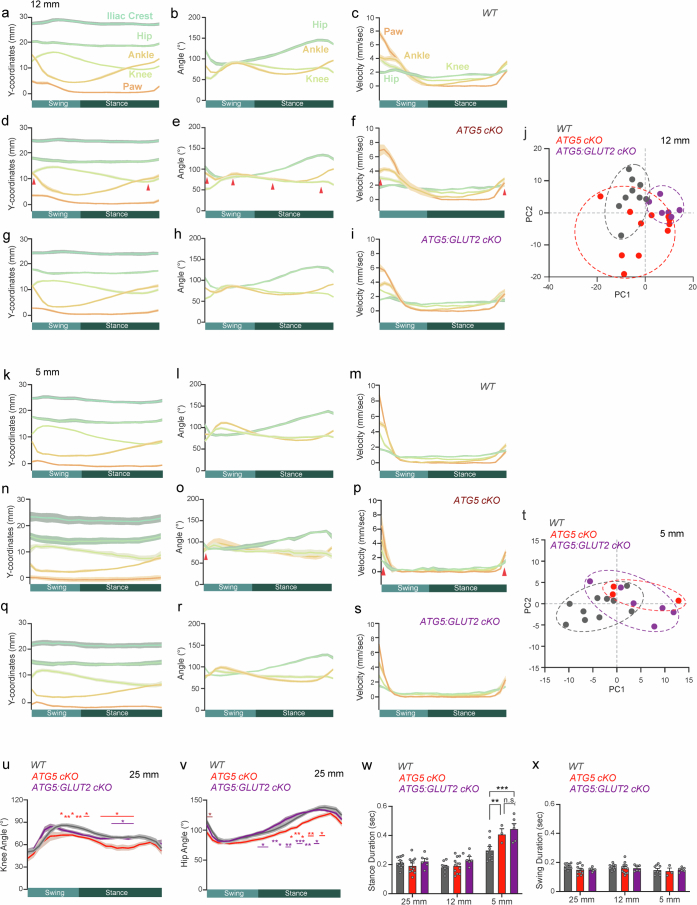
Kinematic analysis of motor coordination in 3-month-old WT, ATG5 cKO and ATG5:GLUT2 cKO mice. **a,d,g**, Y-coordinates of WT (N = 8) (a) ATG5 cKO (N = 11) (d) and ATG5:GLUT2 cKO (N = 6)(g) animals walking on a 12mm-wide beam. **b,e,h**, Hip, knee and ankle angles of WT (N = 8) (b), ATG5 cKO (N = 11) (e) and ATG5:GLUT2 cKO (N = 6) (h) animals walking on a 12mm-wide beam. **c,f,i**, Hip, knee, ankle and hindpaw velocities of WT (N = 8) (c), ATG5 cKO (N = 11) (f) and ATG5:GLUT2 cKO (N = 6) (i) animals walking on a 12mm-wide beam. **j**, PCA analysis based on the y-coordinates, angles, and velocities for 12mm-wide beam. Individual mice are represented as colored circles and dashed lines group mice of similar genotype. **k,n,q**, Y-coordinates of WT (N = 10) (k), ATG5 cKO (N = 3) (n) and ATG5:GLUT2 cKO (N = 6) (q) animals walking on a 5mm-wide beam. **l,o,r**, Hip, knee and ankle angles of WT (N = 10) (l), ATG5 cKO (N = 3) (o) and ATG5:GLUT2 cKO (N = 6) (r) animals walking on a 5mm-wide beam. **m,p,s**, Hip, knee, ankle and hindpaw velocities of WT (N = 10) (m), ATG5 cKO (N = 3) (p) and ATG5:GLUT2 cKO (N = 6) (s) animals walking on a 5mm-wide beam. **t**, PCA analysis based on the y-coordinates, angles, and velocities for 5mm-wide beam. Individual mice are represented as colored circles and dashed lines group mice of similar genotype. **u,v**, Variation in knee (u) and hip (v) angles of WT (N = 9), ATG5 cKO (N = 9) and ATG5:GLUT2 cKO (N = 6) animals walking on a 25mm-wide beam. Statistical significance was calculated with two-way ANOVA followed by Bonferroni’s post-hoc test. **w,x**, Bar graphs showing the duration of stance (w) and swing (x) of WT (N = 9), ATG5 cKO (N = 9) and ATG5:GLUT2 cKO (N = 6) crossing beams of different widths. Statistical significance was calculated with one-way ANOVA followed by Tukey’s post-hoc test. Red arrowheads in (d,e,f,o,p) indicate the points in the step cycle at which the relative positions of the join differ in ATG5 cKOs compared to controls and ATG5:GLUT2 cKOs. In j,k Individual mice are represented as colored circles and dashed lines group mice of similar genotype. All graphs show mean ± SEM. Data presented in Fig. S10b-x show mean as a filled dark line, and SEM as the shaded area around it. n.s.—non-significant; * indicates P ≤ 0.05; ** indicates P ≤ 0.01; *** indicates P ≤ 0.001; **** indicates P ≤ 0.0001.

Lesions in the vermis cause truncal and gait ataxia in humans^66,67^, while PC dysfunction is a common feature in animal models with ataxic symptoms^68^. To investigate whether the conditional deletion of ATG5 in mice leads to gait ataxia through PC loss, we evaluated their motor performance by examining the ability to traverse beams of varying widths: wide (25 mm), regular (12 mm) and narrow (5 mm) beams. As the beam width decreases, mice adjust their intra-limb and inter-limb kinematics to maintain body posture. Dysfunctions in the cerebellar circuitry can manifest as an inability to adapt limb kinematics, resulting in an increased number of slips. At 3 months of age, ATG5 cKO mice, although not impaired in their ability to stay on the rotarod (Extended Data Fig. 9i), revealed a significant increase in footslips when crossing the narrow beam compared to littermate controls (Fig. 7m,n and Supplementary Video 1). This phenotype was consistent both for female and male mice (Extended Data Fig. 9j,k) and was progressive, as 12-month-old ATG5 cKO mice were no longer able to cross the 5-mm beam and showed a significant number of slips even on the wider 12-mm beam (Extended Data Fig. 9l). Strikingly, ATG5:GLUT2 cKO mice resembled the WT controls, displaying fewer footslips on the narrow beam (Fig. 7n and Supplementary Video 1). We further analysed hindlimb movements by tracking kinematics using DeepLabCut and AutoGaitA^69^ (Fig. 7m). Kinematic assessments revealed intra-limb coordination defects in 3-month-old ATG5 cKO mice, even on the wide beam (Fig. 7o–q, Extended Data Fig. 10a–t and Supplementary Videos 2 and 3). The relative positions of the knee and ankle, as well as the coordination of angle aperture and velocity differed in ATG5 cKOs (Fig. 7o,p and Extended Data Fig. 10a–f,k–p) compared to controls and were rescued by GLUT2 deletion (Fig.7q and Extended Data Fig. 10g–i,q–s). Comparison of knee and hip angles confirmed the rescue of hindlimb kinematics, with significant differences observed between ATG5 cKO and ATG5:GLUT2 cKO mice (Extended Data Fig. 10u,v). Principal component analysis (PCA) further supported these findings, clustering controls and ATG5:GLUT2 cKO mice together, while ATG5 cKO mice occupied a different position in the PCA space (Fig. 7r and Extended Data Fig. 10j). Despite the footslip rescue (Fig. 7n and Supplementary Video 1), ATG5:GLUT2 cKO mice, similarly to ATG5 cKO animals, walked with a longer stance phase compared to controls, while swing duration remained unaltered (Extended Data Fig. 10w,x). These kinematic alterations persisted when the mice traversed narrower beams, albeit with smaller differences, likely due to the control and ATG5:GLUT2 cKO mice beginning to adapt their gait to balance perturbations (Extended Data Fig. 10k–t).

Taken together, our data show that conditional loss of ATG5 in mice induces a progressive gait ataxia, manifested by an increase in footslips and disturbed gait kinematics. This phenotype is effectively rescued by simultaneous deletion of GLUT2, with the double mutants showing (1) fewer footslips on the narrow beam compared to ATG5 cKOs (Fig. 7n,b) and (2) improved intra-limb coordination when walking on a wide beam compared to ATG5 cKOs (Fig. 7o–q and Extended Data Fig. 10u,v).

## Discussion

Loss of ATG5 leads to ataxia in humans and flies^70,71^, yet the underlying cellular pathway involved remains elusive. Our study establishes a link between gait ataxia and glycolytic metabolism under autophagy-deficient conditions in mice. We identify persistent dysregulation of the glycolysis pathway in the cerebellum of ATG5 cKO mice, driven by upregulation of GLUT2 in autophagy-deficient PCs. This upregulation results in heightened glucose uptake and increased production of glycolytic by-products, including MG, which has been associated with ataxia in mice^72^, resembling the phenotype observed in the current study. We find that autophagy-deficient PCs undergo neurodegeneration, but their survival in the cerebellar vermis can be prolonged by GLUT2 deletion. These results are consistent with clinical observations, linking lesions or atrophy of the cerebellar vermis to truncal ataxia in humans^73^, as well as with a recent study highlighting the vulnerability of PCs in the posterior vermis in a spinocerebellar ataxia type 1 model^74^. Our findings on gait dysfunction in ATG5 cKO mice align not only with the ataxia phenotype in patients carrying a homozygous missense variant in *ATG5* (ref. ^71^), but also with reports of mutations in autophagy-related genes linked to ataxia in humans. For instance, a mutation in *SNX14* (ref. ^75^) has been associated with SCA20, while ataxia-telangiectasia results from a mutation in *ATM* kinase^76^. While our study points to possible metabolic alterations in patients with an *ATG5* mutation^71^, we acknowledge that current data do not fully elucidate the pathophysiological mechanisms underlying cerebellar degeneration. Further experiments are needed to explore this clinical aspect. Additionally, although our study demonstrates gait rescue in ATG5:GLUT2 cKO mice, we recognize the contribution of other cell types beyond PCs. Gait abnormalities can arise from disruptions in spinal cord circuits^77,78^, although phenotypes in those studies are more severe than in our mutants, restricting locomotion even on broader surfaces. Combined with our findings indicating no alteration in the number of glycinergic interneurons in the spinal cord, and a recent single-cell RNA sequencing study showing a lack of GLUT2 expression in the spinal cord^79^, this suggests that the ataxic gait in ATG5 cKO mice is likely caused by deregulation of a glycolytic pathway in the cerebellum.

Autophagy regulates cellular metabolism through three primary pathways: recycling AAs and/or lipids^13,80^, maintaining energy balance by overseeing mitochondrial quality^81^, and/or modulating the levels of key proteins in metabolic pathways^19–21,50^. Our findings reveal that while cerebellar autophagy is not strictly required for mitochondria quality control and/or AA recycling under basal conditions, it plays a crucial role by regulating GLUT2 degradation. Future work is needed to clarify how exactly GLUT2 is targeted to autophagosome membranes. We hypothesize that GLUT2 degradation may occur through bulk targeting to autophagosomes during starvation, supported by previous findings that nutrient withdrawal stimulates autophagosome formation in PCs^82^. Our findings indicate that disruption of the autophagy pathway in PCs leads to GLUT2 accumulation in VPS35-positive compartments, highlighting the retromer complex’s role in GLUT2 trafficking, similarly to other GLUT transporters^21,53^. In ATG5-deficient cells, GLUT2 may be recycled to the plasma membrane in a retromer-dependent manner rather than degraded, underscoring the interplay between autophagy and the endosomal system in regulating glucose transporter dynamics.

Although the requirement of aerobic glycolysis in neurons has been debatable for decades^83,84^, recent studies indicate that mature neurons require glycolysis in vivo^54,85–87^. Interestingly, the human cerebellum reveals significantly lower aerobic glycolysis rates compared to other brain regions^85,88^. We propose that this attenuation in cerebellar glycolytic activity is influenced by autophagy, which acts as a negative regulator. Several lines of evidence indicate that autophagy is a key negative regulator of cerebellar glycolysis. First, our metabolomics analysis reveals high upregulation of several glycolytic metabolites in the ATG5 cKO cerebellum (Figs. 2 and 3). Second, using [^13^C]glucose as a tracer, we reveal that the loss of ATG5 significantly facilitates the glucose flux into glycolysis, but not the TCA pathway (Fig. 3g and Extended Data Fig. 4). Third, ATG5-deficient PCs, but not cerebellar interneurons, produce significantly more lactate and non-mitochondria-derived ATP (Fig. 4). Finally, ATG5-deficient PCs accumulate MG, a reactive carbonyl species generated endogenously during glycolysis (Fig. 6). While increased glycolytic activity may compensate for the lack of autophagy-mediated quality control^89^, we suggest that autophagy reduces aerobic glycolysis by degrading GLUT2 and mitigating the effects of glycolytic by-products on PC physiology. The metabolic transformation indicates disturbed glucose metabolism as a vulnerability factor in cerebellar neurodegeneration, aligning with cerebellar grey matter reduction in individuals with type 2 diabetes^90^ and gait alterations in older adults with diabetes^91^. This metabolic rewiring also mirrors adaptations seen in other neurodegenerative disorders^25,92,93^ and challenges the notion that mitochondrial dysfunction is the primary cause of movement disorders like Parkinson’s disease^94^, supporting the idea that excessive glycolysis negatively impacts neuronal survival, aligning with recent research^95^.

The heightened glycolytic activity concomitant with increased GLUT2 levels in PCs lacking ATG5 is the central revelation of our study. GLUT2, a class 1 facilitative glucose transporter with a uniquely low glucose affinity^96^, is typically associated with neuronal populations in hypothalamus and brainstem^97–99^. Our findings reveal GLUT2 expression in cerebellar PCs and demonstrate an active mechanism for regulating GLUT2 levels through autophagic degradation. Coupled with a recent study showing modulation of GLUT2 expression by environmental factors like glucose and oxygen availability^100^, our findings suggest that autophagy may adapt GLUT2 expression to changes in glucose availability in the cerebellum.

While we initially used FDG-PET as a marker of neurodegeneration, we acknowledge that GLUT2 upregulation may affect glucose transport and interfere with FDG signal interpretation. Therefore, FDG measurements should be interpreted alongside SV2A-PET signals, which reflect synapse density and can more reliably indicate neurodegenerative changes. Our findings show a decrease in the FDG-PET signal in the ATG5 cKO cerebellum, seemingly inconsistent with increased glucose metabolism in individual ATG5 cKO PCs. While PET provides valuable in vivo resolution, it is constrained spatially. Alterations in glycolysis become apparent as early as 1 month of age, while the FDG-PET study was conducted in 3-month-old mice, a period marked by significant PC loss due to autophagy deficiency. This supports the SV2A-PET study results and suggests that the combined effects of PC loss and/or reduced synapse density—compartments consuming a substantial amount of glucose^101^—together with the limited spatial resolution of FDG-PET, explain the absence of a hypermetabolism phenotype in the ATG5 cKO cerebellum in PET studies.

Our study utilizes the *Atg5*^fl/fl^:*Slc32a1*-Cre mouse line^29^ that allows direct comparison of autophagy-deficient GABAergic neurons across the different brain regions in the same animal. This approach unveiled a distinct region-specific vulnerability of the same neuronal subclass. Our data on the resilience of cerebellar interneurons to ATG5 deletion are consistent with a recent study^102^ and our previous work^28,29^ showing that forebrain interneurons exhibit a robust tolerance to autophagy dysfunction. These results prompt a re-evaluation of existing data linking mutations in ATG proteins to neurodegenerative diseases. For instance, mutations in *WDR45* cause beta-propeller protein-associated neurodegeneration^103^, while mutations in *SPG11*, *ZFYVE26* and *TECPR2* are associated with hereditary spastic paraplegia^104,105^. Although the selective vulnerability of neuronal subclasses to autophagy dysfunctions in these patients warrants further investigation, a recent study reported cerebellar atrophy in a patient with a recurrent mutation in *WDR45* (ref. ^106^), consistent with our findings.

In conclusion, our findings highlight the complex interplay between autophagy, glycolysis and PC susceptibility to neurodegeneration. This research not only sheds light on cerebellar pathophysiology but also provides a framework for understanding broader neurodegenerative processes. Targeting metabolic vulnerabilities, such as glycolytic dysregulation and GLUT2 perturbations, may pave the way for therapeutic interventions aimed at modifying cellular metabolism to mitigate neurodegenerative impacts.

## Methods

### Mouse models

All animal experiments were approved and performed according to the regulations of the LANUV, NRW, Germany (AZ: 81-02.04.2020.A418, 81-02.05.40.20.075, 81-02.04.2021.A132, 81-02.04.2021.A067, 81-02.04.2022.A116) guidelines. Mice were maintained in a pathogen-free environment in ventilated polycarbonate cages and housed in groups of five animals per cage with constant temperature and humidity at 12-h/12-h light–dark cycles. Food and water were provided ad libitum. All mice originate from the mixed C57BL/6JRj (≈40–48%); C57BL/6J (≈25–45%); C57BL/NRj (≈5–20%); FVB/N (≈1–6%) genetic background. *Atg5*flox:*Slc32a1*-Cre:tdTomato (ATG5 cKO) mice were described previously^29^. To create ATG5:GLUT2 cKO mice, mice with floxed exon 3 of the *Atg5* gene were crossed with mice, where the exon 11 of the *Slc2a2* gene was flanked by loxP (kindly provided by B.T., University of Lausanne)^62,63^. Supplementary Table 1 indicates genotyping primers used to genotype the animals. In accordance with the SAGER guidelines, we have ensured that both male and female mice were included in all experimental groups.

### PET imaging

Animals were anaesthetized with isoflurane in O_2_/air at a 3:7 ratio (induction 5%, maintenance 1.5–2.0%), and a catheter for tracer injection ([^18^F]UCB-H in 3-month-old mice, [^18^F]MNI1126 in 12-month-old mice) was inserted into the lateral tail vein. A PET scan in list mode was conducted using a Focus 220 micro-PET scanner (CTI-Siemens) with a resolution at the centre of field of view of 1.4 mm. Data acquisition started with intravenous tracer injection (activity: 6–12 MBq in 125 µl) and lasted for 40 min. This was followed by a 10-min transmission scan using a ^57^Co point source for attenuation correction. For [^18^F]FDG-PET, 9–12 MBq [^18^F]FDG in 125 µl was injected intraperitoneally. The mice were then placed in a solitary cage where they spent the following 35 min awake. Subsequently, they were anaesthetized again and scanned for 30 min under anaesthesia. After full 3D rebinning, summed images were reconstructed using an iterative OSEM3D/MAP procedure^107^, resulting in voxel sizes of 0.47 × 0.47 × 0.80 mm. For all further processing of the images including statistics, the software VINCI 5.21 for MacOS X (MPI for Metabolism Research, Cologne, Germany) was used. Images were co-registered and intensity-normalized to the thalamus. To this end, an elliptical volume of interest of 7.2 mm^3^ (40 voxels) was placed inside the thalamus. Each image was divided by the mean value of the thalamus volume of interest, resulting in the ‘standardized uptake value ratio’ (SUVR_Thal_). No further postprocessing was done. For comparison of ATG5 cKO versus WT, a voxel-wise two-tailed unpaired *t*-test was performed for each tracer using VINCI 5.21 for MacOS X. The resulting t-maps were corrected for multiple comparisons using a threshold-free cluster enhancement (TFCE) procedure described in detail in ref. ^108^. The TFCE procedure was implemented as a Python script in VINCI. For final thresholding at *P* < 0.05, a permutation test with 10,000 permutations was performed in RStudio 1.0.153 for MaxOS X using the SUVR_Thal_ values of the voxel with the highest TFCE value. The 95% quantile was calculated, and the corresponding TFCE level was used as the lower threshold of the t_TFCE_ map. The resulting t_TFCE_ maps were displayed in voxel view in shades of red (ATG5 cKO > WT) or blue (ATG5 cKO < WT) and projected onto a C57BL/6 T2-weighted magnetic resonance imaging template.

### Primary cerebellar culture

Mice were euthanized at P7–9. Cerebellum was chopped into 600-µm-thick pieces and incubated in solution A (PBS containing 13 mM glucose, 300 mg BSA (fatty acid-free), 1.5 mM MgSO_4_) containing 0.25 mg trypsin for 15 min at 37 °C. To stop trypsinization, solution A, containing 0.5 mg Soybean Trypsin Inhibitor and 600 U DNase twice the original volume was added and samples were centrifuged at 1,000*g* for 1 min at 4 °C. Cell suspension was dissolved in DMEM-based (Gibco) growth medium containing 0.4% B-27 (50× Gibco), 19 mM KCl (Roth), 14 mM HEPES (Roth), 10 mM glucose (Sigma), 1 mM sodium pyruvate (100x Gibco), 0.25% GlutaMax (100× Gibco), 1% penicillin–streptomycin (10,000 µg ml^−1^ streptomycin, Thermo Fisher Scientific), 10% FBS (Sigma) and mechanistically dissociated. Cells were layered on top of EBSS (Gibco), containing 4% (wt/vol) BSA, 3 mM MgSO_4_ and centrifuged at 1,500*g* for 5 min at 4 °C. Cells were dissolved in growth medium and plated at a density of 750,000. After 24 h and after 7 days, half of the medium was replaced by fresh growth medium containing 4 µM AraC (Merck).

### Seahorse assay

Metabolic measurements were carried out in a Seahorse XF96 Analyzer. Cerebellar cells were plated in 96-well Seahorse XF Cell Culture Microplates and analysed at days in vitro (DIV) 17. For the Mito Stress Test (Agilent), growth medium was replaced by Seahorse XF Base Medium supplemented with 1 mM pyruvate, 2 mM glutamine and 10 mM glucose and equilibrated in a 37 °C non-CO_2_ incubator 1 h before the experiment. Following the baseline measurements for oxygen consumption rate (OCR), 1.5 µM oligomycin A, 2 µM FCCP and 0.5 µM rotenone–antimycin A combined with Hoechst (Thermo Fisher, 62249) were sequentially added. For the glycolysis stress test (Agilent), growth medium was replaced by Seahorse XF Base Medium supplemented with 2 mM glutamine and equilibrated in a 37 °C non-CO_2_ incubator 1 h before the experiment. Baseline ECAR was measured followed by sequential adding of 10 mM glucose, 1.5 µM oligomycin A and 50 mM 2-DG combined with Hoechst injection. OCR and ECAR values were normalized to cell density based on the Hoechst signal via a Cytation microplate reader (Agilent).

### Preparation of OTCs

Mice were euthanized at P6–9, cerebellum was collected in ice-cold HBSS (Thermo Fisher Scientific) and cut into 300-µm-thick sagittal sections. Sections were washed three times in pre-warmed (37 °C) HBSS. Slices were then transferred onto membrane inserts (Merck) with pre-warmed OTC MEM-based (Sigma) medium containing 0.00125% ascorbic acid (Roth), 10 mM d-glucose (Sigma), 1 mM GlutaMAX (100× Gibco), 20% (vol/vol) horse serum (Gibco), 0.01 mg ml^−1^ insulin (Thermo Fisher Scientific), 14.4 mM NaCl (Roth), 1% penicillin–streptomycin (Thermo Fisher Scientific). Medium was replaced every second day. OTCs were used at DIV21 either for live imaging or for IHC. For IHC, slices were fixed in 4% paraformaldehyde (PFA) for 30 min at room temperature (RT) washed three times with PBS and then processed as fixed brain sections (see below).

### Live imaging in cerebellar OTCs

Viral transduction was done on DIV1 by adding 1 µl of AAV (Supplementary Table 2) on top of each slice. Live imaging was performed at DIV21 in carbogenated OTC imaging medium (distilled water, 2 mM CaCl_2_, 10 mM d-glucose, 3 mM KCl, 1 mM MgCl_2_, 136 mM NaCl, 24 mM NaHCO_3_, 1.25 mM NaH_2_HPO_4_) after constant perfusion. For all live imaging, the TCS SP8 MP-OPO (Leica) multiphoton confocal microscope was used equipped with a ×25/0.95 multi-immersion objective. For ATeam and Laconic, single-plane scans were acquired at a 512 × 512 scanning format of bidirectional recording of one frame per second, yielding a pixel size of 0.86 µm. For ratiometric FRET measurements of ATeam and Laconic, the donor was excited at 448 nm, and emission of both eCFP/mTFP and mVenus were captured by individual detectors between 450 nm and 500 nm for eCFP/mTFP and between 520 nm and 570 nm for acceptor mVenus. Recordings were analysed using ImageJ (Fiji) by measuring fluorescence intensity of donor and acceptor individually in PC or molecular cell layer interneuron soma after background subtraction. The ratio of mVenus:eCFP or mTFP:mVenus was used as a read-out of ATP or lactate levels, respectively. Slices were recorded at baseline, after 30 min of incubation after constant perfusion with 1.5 µM oligomycin A (Sigma-Aldrich, 495455) or 1.5 µM oligomycin A and 10 mM 2-DG (Roth, CN96.3) or after 5 min of constant perfusion with 5 mM pyruvate. AUC was determined by integrating the fluorescence signal ratio for the time of recording individually for baseline (30 s), 1.5 µM oligomycin A (30 s), 1.5 µM oligomycin A and 10 mM 2-DG (30 s) for ATeam and baseline (20 s) and 5 mM pyruvate (20 s) for Laconic using GraphPad Prism. For GCaMP7f imaging, single-plane scans were acquired at a 512 × 512 scanning format of bidirectional recording of one frame per second, yielding a pixel size of 0.86 µm. Videos of 60 s in length were recorded. After 20 s of baseline recordings, slices were stimulated once with 100 APs at 100 Hz using an RC-47FSLP stimulation chamber (Warner Instruments). Recordings were analysed using ImageJ (Fiji). Baseline fluorescence, after background subtraction, of PCs was measured by averaging fluorescence of the first 19 frames. Fluorescence values of each frame were then normalized to baseline. AUC was determined by integrating the normalized Δ*F/F* 20 s before and 10 s after the stimulation using GraphPad Prism. For mt-mKeima, mKeima-GLUT2 and mCherry-EGFP-GLUT2 live imaging, single-plane images were acquired at a 1,024 × 1,024 scanning format of bidirectional recording of one frame per second, yielding a pixel size of 0.43 µm. Images were analysed using ImageJ (Fiji) by measuring fluorescence intensity individually in PC soma after background subtraction. For induced mitophagy, slices were pretreated with 10 µM CCCP (Thermo Scientific, L06932.ME) for 6 h.

### NBDG uptake

For 2-NBDG uptake, OTCs were incubated in OTC medium without glucose supplemented with 1 mM 2-NBDG (Biomol, ABD-36702) for 30 min at 37 °C in the dark and then washed twice with OTC imaging medium. For imaging, the TCS SP8 MP-OPO confocal microscope was used, equipped with a ×25/0.95 multi-immersion objective. Single-plane images were acquired at a 1,024 × 1,024 scanning format with bidirectional recording of one frame per second, yielding a pixel size of 0.43 µm. Recordings were analysed using ImageJ (Fiji). Mean grey values, after background subtraction, were used as fluorescence read-out of protein levels in PC or molecular layer interneuron soma.

### PC density analysis in OTCs

OTCs were obtained as described above. Slices were cultured either in OTC medium with 5 µM d-serine (Merck, S4250) for 3 weeks, with 100 nM LPA (Merck, 857130P) for the last 7 days, or with 1 µM RAS-selective lethal 3 (RSL3) (Selleckchem, S8155) and 10 µM Fer1 (Cayman, 17729) for 24 h and with vehicle, respectively, at 37 °C and 5% CO_2_. For combined LPA and Fer1 treatments (and d-serine and Fer1 treatments), slices were cultured in OTC medium with vehicle or 100 nM LPA (or 10 µM d-serine) for the last 7 days, where 5 µM Fer1 was added for the last 4 days. At DIV21, slices were fixed in 4% PFA for 30 min at RT, washed three times with PBS and then processed for IHC as fixed brain sections (described below). The sections were imaged using a Leica Stellaris 5 equipped with a HC PL APO ×20/0.75 CS2 objective and a continuous excitation white-light laser. Samples were scanned at a resolution of 1,024 × 1,024 with bidirectional recording yielding a pixel size of 0.57 µm. Stacks of 8–10 optical sections with a fixed *z* plane of 680 nm were taken. Images were visualized using Fiji (ImageJ).

### Preparation of acute slices, starvation and chloroquine treatment

Mice were euthanized at the age of 3 months via cervical dislocation, and brains were isolated. Cortex and cerebellum were separated and cut with a vibratome (Leica) into 150-μm horizontal sections in ice-cold, carbogen-saturated (95% O_2_ and 5% CO_2_), low-Ca^2+^, osmolarity-adjusted ACSF (125 mM NaCl, 2.5 mM KCl, 1.25 mM NaH_2_PO_4_, 25 mM NaHCO_3_, 10 mM glucose, 0.5 mM CaCl_2_, 3.5 mM MgCl_2_, pH 7.4). Cerebellar and cortical acute slices were incubated in control OTC medium, control medium with 400 µM chloroquine (Sigma, C6628) or ACSF for 6 h at 37 °C and 5% CO_2_. Afterwards, samples were shock frozen in liquid nitrogen and stored at −80 °C until further processing.

### Preparation of acute slices and CHX treatment

Cerebellar acute slices (100 µm) were obtained as described above and incubated in OTC medium, control medium with 60 µM CHX (Sigma-Aldrich, 239763), ACSF or ACSF with 60 µM CHX for 3.5 h and 7 h at 37 °C and 5% CO_2_. Additionally, acute slices were incubated in control OTC medium or control medium with 60 µM CHX for 7 h at 37 °C and 5% CO_2_. Next, 10 µM puromycin (Roth, 0240.1) was added for the last 1.5 h of incubation. Afterwards, samples were fixed with 4% PFA for 1 h at RT and further processed for IHC (see below).

### Preparation of acute slices and antibody uptake assay

Cerebellar acute slices were prepared as described above. Slices were incubated in ACSF for 1 h at 37 °C and 5% CO_2_ followed by incubation in ACSF with vehicle, ACSF with 50 µM Pitstop2 (Abcam, ab120687) or ACSF with 500 nM ULK1-inhibitor (SBI-0206965; Merck, SML1540) for 1 h at 37 °C and 5% CO_2_. Afterwards, 16 µg ml^−1^ extracellular GLUT2 antibody (Merck) was added to each well and slices were incubated for another 1.5 h at 37 °C and 5% CO_2_. Slices were then washed once with ice-cold 0.2 M acetic acid, fixed in 4% PFA for 1 h at RT and washed three times with 1× PBS and further processed for IHC (as described below). Transferrin uptake assay was performed by incubating the slices with 25 µg ml^−1^ human transferrin conjugated to Alexa Fluor 488 (Invitrogen, T13342) instead of the primary antibody.

### Untargeted metabolomics

Untargeted metabolomics was performed on fresh-frozen 1-month-old and 3-month-old cerebellum. Metabolites were extracted in extraction solution (LC–MS-grade methanol 50%, LC–MS-grade acetonitrile 30%, ultrapure water 20%, valine-d8 final concentration 5 µM), and tissue was lysed in a homogenizer (2 × 30-s cycles at 3,500*g* at 4 °C). Tissue extracts were shaken in a Thermomixer (Eppendorf) at full speed for 15 min at 4 °C and centrifuged at full speed for 20 min at 4 °C. Equal amounts of each sample were used for further analysis. Metabolites were separated on a Millipore SeQuant ZIC-pHILIC column (5 µm, 2.1 × 150 mm) with a 2.1 × 20-mm guard column, using a binary solvent system. Solvent A was 20 mM ammonium carbonate with 0.05% ammonium hydroxide, and solvent B was acetonitrile. The column oven was set to 40 °C, and the autosampler to 4 °C. The gradient (0.200 ml min^−1^) started at 80% B (0–2 min), decreased linearly to 20% B (2–17 min), returned to 80% B (17–17.1 min), and held until 23 min. Samples (5 µl) were injected in random order, with quality-control samples analysed regularly. Data were acquired on Vanquish Horizon UHPLC connected to the Thermo Fisher Orbitrap Exploris 240 mass spectrometer with HESI source. Spray voltages were configured at +3.5 kV/−2.8 kV, the RF lens value at 70, the heated capillary temperature at 320 °C and the auxiliary gas heater at 280 °C. Sheath, aux and sweep gas flow rates were set at 40, 15 and 0 arbitrary units, respectively. For MS1 scans, data were acquired from *m/z* 70–900 with standard automatic gain control and auto-set injection time in full scan mode with polarity switching at a resolution of 120,000. The AcquireX Deep Scan workflow used iterative data-dependent acquisition, with pooled sample injections, 60,000 full scan resolution, 30,000 fragmentation resolution and a 5.0e3 intensity threshold. Dynamic exclusion was set to 10 s with a 5-ppm mass tolerance and 1.2 *m/z* isolation window. Stepped HCD collision energies were 30, 50 and 150. Metabolite identification used Compound Discoverer v3.2 with criteria of precursor ion *m/z* within 5 ppm, fragment ion matching within 5 ppm to a spectral library, a minimum match score of 70 and retention time within 5% of standards. Peak area integration and chromatogram review were performed in TraceFinder v5.0. Metabolite intensities were preprocessed using the 80% filtering rule per condition. Missing values were imputed using the half minimum value method152, followed by probabilistic quotient normalization 152.e^109^. Outliers were assessed using Hotteling’s T2 method and PCA plots. Differential metabolite abundance was determined using *t*-tests (R ‘stats’ package), and pathway analysis was performed using MetaboAnalyst 5.0.

### ^13^C_6-_glucose tracing using liquid chromatography–high-resolution MS

Cerebellar acute slices prepared from 3-month-old mice were incubated in ACSF containing 10 mM [^12^C_6_]glucose (unlabelled) or 1 mM ^13^C_6_-labelled glucose for 1 h at 37 °C and 5% CO_2_. The slices were transferred to tubes, washed once in 75 mM ammonium carbonate and the fresh weight of samples was determined. To each of the samples, a 5-mm stainless-steel metal ball was added before snap-freezing them in liquid nitrogen. The frozen samples were pulverized for 1 min using a TissueLyser (Qiagen) set to 25 Hz for 1 min. Immediately after homogenization, 1 ml of −20 °C mixture of UPLC-grade acetonitrile:methanol:water (2:2:1 (vol:vol:vol)), containing 375 nmol U-^13^C^15^N AA mix (Cambridge Isotopes, MSK_A2-1.2), each 150 ng ml^−1^ of ^13^C_10_ ATP, ^15^N_5_ ADP and ^13^C_10_^15^N_5_ AMP (Sigma) and 200 ng ml^−1^ of citric acid ^2^H_4_ (Sigma) was added. After addition of the extraction buffer, the samples were immediately vortexed for 10 s before incubating them for additional 30 min at 4 °C on an orbital shaker. Insoluble material was removed from each sample and the tubes were centrifuged for 10 min at 21,000*g* at 4 °C. The cleared supernatant was transferred to a fresh 1.5 ml microcentrifuge tube and immediately concentrated to complete dryness in a speed vacuum concentrator set to 20 °C. For the LC–MS analysis, the dried samples were resuspended in 150 µl of ice-cold UPLC-grade water, vortexed thoroughly and centrifuged for 5 min at 21,000*g* at 4 °C. The cleared supernatant was analysed as described in ref. ^110^. The LC–MS data analysis of the amine, glycolysis and TCA compounds was performed using the TraceFinder software (version 5.1, Thermo Fisher Scientific). The identity of each compound was validated by authentic reference compounds, which were measured at the beginning and the end of the sequence. For data analysis, the area of all detectable isotopologues mass peaks of every required compound were extracted and integrated using a mass accuracy of <3 ppm and a retention time tolerance of <0.05 min as compared to the independently measured reference compounds. If no independent ^12^C experiments were carried out, where the pool size was determined from the obtained peak area of the ^12^C monoisotopologue, the pool size determination was carried out by summing the peak areas of all detectable isotopologues per compound. These areas were then normalized, as performed for un-traced ^12^C experiments, to the internal standards, which were added to the extraction buffer, followed by a normalization to the fresh weight of the analysed samples. The relative isotope distribution of each isotopologue was calculated after natural abundance correction from the proportion of the peak area of each isotopologue towards the sum of all detectable isotopologues.

### Targeted metabolomics

Targeted metabolomics analyses were performed in fresh-frozen cerebellum from 1-month-old and 3-month-old mice. Intermediates of the glycolysis and the TCA cycle were determined by anion-exchange chromatography coupled to electrospray ionization high-resolution MS using a procedure previously described^111^. Approximately 50 mg of tissue were homogenized in ice-cold acetonitrile/methanol/water at a 2:2:1 ratio (vol/vol/vol; (1 mg/10 µl) using the Precellys 24 Homogenizer (Peqlab). Then, 100 µl of homogenate was mixed with a further 225 µl of acetonitrile/methanol/water at a 2:2:1 ratio (vol/vol/vol) and 25 µl of a mixture of isotope-labelled internal standards in Milli-Q water (5 µM ^13^C_6_-d-glucose-6-phosphate and 5 µM D4-succinic acid, both Eurisotop). After thorough mixing and centrifugation (16,100*g*, 5 min, 4 °C), 300 µl of supernatant was dried under reduced pressure. The residue was resolved in 100 µl of Milli-Q water, transferred to autoinjector vials and immediately measured. Ion chromatography high resolution mass spectrometry analysis was performed using a Dionex Integrion RFIC system (Thermo Scientific) coupled to a Q Exactive HF mass spectrometer (Thermo Scientific) as previously described^111,112^. The exact *m/z* traces of the internal standards and endogenous metabolites were extracted and integrated using Skyline 21.2.0.369 (open source). Endogenous metabolites were quantified by normalizing their peak areas to those of the internal standards. AAs and GABA were derivatized with benzoyl chloride and quantified by liquid chromatography coupled to electrospray ionization tandem mass spectrometry using a procedure previously described^112^. Homogenates were centrifuged (16,100*g*, 5 min, 4 °C). Next, 20 µl of the supernatant was mixed with 10 μl of the MassChrom internal standard mixture AAs and acylcarnitines from dried blood (Chromsystems), reconstituted in 5 ml water/methanol at a 2:1 ratio (vol/vol), and 10 μl of a 10 µM solution of d6-GABA (Sigma-Aldrich) in Milli-Q water. Endogenous metabolites and internal standards were derivatized by adding 10 µl of freshly prepared 2% benzoyl chloride in acetonitrile and 10 µl of 100 mM sodium carbonate in water and thorough mixing. After addition of 40 µl of Milli-Q water and centrifugation (16,100*g*, 5 min, 4 °C), 80 µl of the supernatant was transferred to autoinjector vials and immediately measured. LC–MS/MS analysis was performed by using a Nexera X2 UHPLC System (Shimadzu) coupled to a QTRAP 6500 mass spectrometer (SCIEX) as previously described^111–113^. The LC chromatogram peaks of benzoylated metabolites were integrated using the MultiQuant 3.0.2 software (SCIEX). The peak areas of the endogenous metabolites were normalized to those of the internal standards.

### Proteomics

Total proteome analysis was performed on fresh-frozen cerebellum from 1-month-old and 3-month-old animals. Samples were homogenized and lysed in 8 M urea followed by reduction (5 mM dithiothreitol (DTT)) and alkylation of cysteines (40 mM chloroacetamide). Samples were digested using LysC for 4 h followed by dilution of urea and overnight digestion using trypsin (both with a 1:75 ratio). Afterwards, samples were acidified and clean-up was performed using mixed-mode StageTips^114^. Next, 50 µg of each sample was analysed by the CECAD Proteomics Facility on an Orbitrap Exploris 480 (granted by the German Research Foundation under INST 1856/71-1 FUGG) mass spectrometer equipped with a FAIMSpro differential ion mobility device that was coupled to an UltiMate 3000 (Thermo Scientific). Samples were loaded onto a precolumn (Acclaim 5 µm PepMap 300 µ Cartridge) and then reverse-flushed onto an in-house packed analytical column (30 cm length, 75 µm inner diameter, filled with 2.7 µm Poroshell EC120 C18, Agilent). Peptides were chromatographically separated running eluent A (0.01% formic acid) against eluent B (80% acetonitrile, 0.1% formic acid) using a linear gradient between 6% and 55% B followed by washing and reequilibration. The mass spectrometer was operated in data-independent acquisition (DIA) mode using 60 × 10 *m/z* windows with 1 *m/z* overlap between 400 *m/z* and 1,000 *m/z* at a resolution of 15,000. A gas-phase fractionated spectral library was built by injecting a pooled sample six times covering the analytical range in 100 *m/z* steps using staggered 4 *m/z* windows at a resolution of 30,000, resulting in nominal 2 *m/z* windows after deconvolution using ProteoWizard. Library building and subsequent data analysis were performed in DIA-NN 1.8.1 using a Swiss-Prot mouse canonical database (UP589, downloaded 04 January 2022) and the additional command line prompts ‘--report-lib-info’ and ‘--relaxed-prot-inf’ with otherwise standard parameters. DIA-NN output was further filtered on library *q* value and global *q* value ≤ 0.01 and at least two unique peptides per protein using R (4.1.3). Finally, LFQ values were calculated using the DIA-NN R package. Afterwards, analysis of results was performed in Perseus 1.6.15. Pathway analysis was performed using ShinyGO 0.81 (South Dakota State University).

### Immunohistochemical analysis of brain sections

Mice were euthanized at 1, 3 and 12 months of age by an overdose of 1.2% ketamine, and 0.16% xylazine in PBS, transcardially perfused, dissected and postfixed in 4% PFA (pH 7.4) overnight. Horizontal free-floating 40-µm sections were blocked with 10% normal goat serum (NGS) or 10% normal donkey serum (NDS) in 0.5 % Triton-X in PBS for 1 h at RT. Primary antibodies (Supplementary Table 3) were incubated in 3% NGS and 0.3% PBS-T for 48 h at 4 °C. Sections were washed in 0.3% PBS-T before incubation with secondary antibodies in 3% NGS or NDS and 0.3% PBS-T for 2 h at RT (Supplementary Table 3). The sections were imaged using either a Leica TCS SP8 equipped with an Apo ×40/0.85 CORR CS and PL Apo ×63/1.40 oil CS2 or a Leica Stellaris 5 equipped with HC PL Apo ×20/0.75 CS2 and an Apo ×63/1.32 FLYC CORR CS2 objective and a continuous excitation white-light laser. Samples were scanned at a resolution of 1,024 × 1,024 pixels with bidirectional recording, and stacks of 20–30 optical sections with a fixed *z* plane of 533 nm (for ×63 and ×40) and 685 nm (for ×20) were taken with pixel sizes of 0.284 µm (for ×40), 0.180 µm (for ×63) and 0.568 µm (for ×20). Images were visualized using Fiji (ImageJ). Mean grey values, after background subtraction, were used as fluorescence read-out of protein levels in the region of interest, containing either PC soma, PC dendrite or molecular layer interneurons. For colocalization and 3D analysis, samples were scanned at a resolution of 1,024 × 1,024 pixels with bidirectional recording using a Leica Stellaris 5 equipped with a Plan-Apochromat ×63/1.30 GLYC objective with a pixel size of 0.180 µm. Stacks of 20–30 optical sections were taken with a fixed z plane of 250 nm. Reconstructions and colocalization analysis were performed with Amira 2020.2 (Thermo Fisher Scientific). For cresyl violet staining, 40-μm horizontal sections were mounted, hydrophylized in water for 1 min before incubation in cresyl violet solution for 7 min. Sections were then washed three times in water for 2 min before being dehydrated in ascending ethanol solutions (70%, 80%, 90%, 96%, 100%) for 2 min each. Afterwards, sections were incubated in xylene for 2 min and then covered with mounting solution Entellan (Merck). Images were acquired on a S360 Hamamatsu slide scanner using a ×40 objective and a pixel size of 0.230 µm. Aperio ImageScope viewing software (Leica, version 12.4.3.5008) was used for analysis. For illustrative purposes, images for both conditions (WT and ATG5 cKO) were multiplied by the same factor using the ImageJ Math option (changes were applied to the entire images in both conditions).

### EM

Animals at the age of 3 months were perfused using 2% formaldehyde (Science Services) and 2.5% glutaraldehyde (Merck) in 0.1 M cacodylate buffer. Brains were postfixed and 40-µm horizontal sections were prepared. Regions of interest were extracted using a biopsy punch and postfixation was applied using 1% osmium tetroxid (Science Services) and 1% potassium hexacyanoferrat (Merck). Samples were dehydrated using ascending ethanol series (50%, 70%, 90%, 100%). Infiltration was carried out with a mixture of 50% Epon–ethanol for 1 h, 70% Epon–ethanol for 2 h and overnight with pure Epon (Merck). After fresh Epon for 4 h, sections were mounted onto empty Epon blocks and covered with Aclar foil. After 48 h hardening at 60 °C, Aclar foil was removed and samples were trimmed to the region of interest. Ultrathin sections (70 nm) were cut using a diamond knife (Science Services) on an UC6 ultramicrotome (Leica) and collected onto Pioloform-coated slot grids. Poststaining was performed with 1.5% uranyl acetate (Agar Scientific) for 15 min and Reynolds lead citrate (Roth) solution for 3 min. Images were acquired using a JEM-2100 Plus transmission electron microscope (JEOL) operating at 80 kV equipped with a OneView 4 K camera (Gatan). EM quantifications were carried out manually on acquired images (10,000) of PC somata. Number of mitochondria were counted and normalized to the total area of the cell. The total area of single mitochondria and the total length (perimeter) of the corresponding cristae was analysed. Cristae length was then normalized to the total area of mitochondria.

### RNAscope of spinal cord sections

Mice were euthanized and transcardially perfused as described above. Spinal cords were dissected and postfixed for 1 h at RT, then rinsed three times in PBS and cryoprotected in 30% sucrose in PBS overnight at 4 °C. Tissue was cryosectioned at 14 μm using a Leica CM3050 cryostat. Multiple-labelling fluorescence in situ hybridization was performed using the RNAscope Multiplex Fluorescent Reagent Kit v2 Assay (Advanced Cell Diagnostics, 323100), following the manufacturer’s recommended protocol. A mix of three probes was used: *Mm-Slc32a1* revealed with Opal 520 (Akoya), *Mm-Slc17a6* revealed with Opal 570 (Akoya) and *Mm-Slc6a5* revealed with Opal 650 (Akoya). Sections were mounted using Aqua-Poly/Mount (Polysciences). The sections were imaged using a Leica TCS SP8 (Leica Microsystems) equipped with a PL Apo ×40/0.85 CORR CS objective and a continuous excitation white-light laser. Samples were imaged using a 2,048 × 2,048 scanning format with bidirectional recording, yielding a pixel size of 0.125 µm. Stacks of six optical sections with a fixed *z* plane of 4 µm were taken. The ImageJ Cell Count plugin was used to visualize and count neurons. Neurons were assigned to dorsal versus ventral laminar location based on their relative position with respect to the central canal. Nine hemisections per mouse were analysed, and they included an equal sampling of cervical and lumbar segments.

### Immunoblotting analysis

Mice were euthanized at the age of P7, 1 month, 3 months and/or 12 months via cervical dislocation. Samples were homogenized in RIPA buffer (dH_2_O; 1% Igepal; 150 nM NaCl; 0.1% SDS; 0.5% SOD; 50 mM Tris) containing protease inhibitor (Roche) and phosphatase inhibitor (Thermo Scientific) using a Wheaton otter-Elvehjem Tissue Grinder. Afterwards, samples were sonicated (ten pulses), incubated on ice for 45 min and centrifuged at 16,200*g* for 15 min at 4 °C. Protein concentrations were assessed using the Bradford assay (Sigma). Next, 10–20 μg protein per sample was loaded onto SDS–PAGE gels for protein separation and then transferred onto nitrocellulose or methanol-activated PVDF membranes. Membranes were blocked in 5% milk or BSA in TBS containing 1% Tween (TBS-T) for 1 h at RT followed by primary antibody (Supplementary Table 4) incubation in TBS overnight at 4 °C. Afterwards, membranes were washed three times with TBS-T and then incubated with horseradish peroxidase-tagged secondary antibodies for 1 h at RT followed by three washes in TBS-T at RT. Protein levels were visualized using ECL-based autoradiography film system (Super RX-N, Fujifilm) or ChemiDoc Imaging system (Bio-Rad) and analysed using the Gel Analyzer plugin from ImageJ (Fiji).

### Surface biotinylation assay on acute cerebellar slices

Cerebellar acute slices of 150 µm in thickness were obtained as described above. Slices were washed in 37 °C pre-warmed ACSF (see above) for 30 min before incubation in ACSF containing 1 mg ml^−1^ EZ-Link Sulfo-NHS-SS-Biotin (Thermo Fisher Scientific, 21331) for 1 h at 4 °C. Afterwards, sections were incubated in ice-cold ACSF containing 100 mM glycine for 30 min at 4 °C and washed three times with ice-cold ACSF. Proteins were extracted as described above. Supernatant was used as the input fraction. Around 500–800 µg of protein was incubated with an appropriate amount of prewashed Streptavidin beads (Thermo Fisher Scientific, 20357) overnight at 4 °C. The next day, samples were centrifuged at 16,200*g* for 30 s at 4 °C and the supernatant was collected as non-biotinylated (unbound) fraction. Beads were washed three times with ice-cold RIPA buffer and once with 50 mM HCl. Samples were centrifuged at 16,200*g* for 30 s at 4 °C. For protein elution, 40 µl of 2× SDS + 50 mM DTT was added to the beads and incubated for 5 min at 95 °C. Collected samples contained biotinylated proteins. The input and non-biotinylated fractions were equally mixed with 2× SDS + 50 mM DTT.

### AAV production

pAAV-mDlx-EGFP-ATG5 was generated from pEGFP-C1-hApg5 (Addgene, 22952) and pAAV-mDlx-GFP-Fishell-1 (Addgene, 83900) by restriction subcloning. EGFP-hATG5 was amplified (see primer sequence in Supplementary Table 1), PCR product and pAAV-mDlx-GFP-Fishell-1 were digested, using SgsI-FD and NcoI-FD (both ThermoFisher Scientific), and ligated with T4 DNA Ligase (NEB). A pAAV/L7-6-EGFP-WPRE construct was generated from pAAV/L7-6-GFP-WPRE (Addgene, 126462) and the pAAV backbone from pAAV-hSyn-mScarlet (Addgene, 131001) using NEBuilder HiFi DNA Assembly (NEB). Subsequently, pAAV/L7-6-mKeima-Red-WPRE was generated from pAAV/L7-6-EGFP-WPRE and mKeima-Red-N1 (Addgene, 54597) using NcoI and HindIII restriction sites. Additionally, for the generation of pAAV/L7-6-mKeima-Red-rGLUT2-WPRE, ratGLUT2 (Addgene plasmid, 14946NM_012879.2: c.1080 A > G, c.1417 A > G, c.1528 G > A, c.1554 T > A) was used and fragments assembled using NEBuilder HiFi DNA Assembly (NEB). The plasmids pAdDeltaF6 (Addgene plasmid, 112867) and pAAV2/rh10 (Addgene plasmid, 112866) were a gift from J. M. Wilson. All DNA constructs were confirmed by Sanger sequencing (Eurofins). Recombinant AAV2/rh10 particles were prepared in HEK 293T cells (DSMZ no. ACC 635) by transfecting AAV genome plasmids together with pAdDeltaF6 and pAAV2/rh10. Viral particles were precipitated with PEG/NaCl and cleared with chloroform extraction. For in vivo applications, AAVs were purified by adapting scalable anion-exchange chromatography strategies^115^. Cleared AAVs were concentrated roughly 20-fold with prewashed (PBS + 0.001% (vol/vol) Poloxamer 188, Sigma-Aldrich) 100-kDa Amicon filters (Merck Millipore) and diluted tenfold in buffer A (10 mM Bis–Tris-Propane pH 9.0, 1 mM MgCl_2_). AAVs were applied at a flow rate of 3 ml min^−1^ to a self-packed 1 ml column (POROSTM HQ 50 µm strong anion-exchange resin, Thermo Fisher Scientific), which was equilibrated in buffer A. After injection, the column was rinsed with 20 column volumes of buffer A, washed with 20 column volumes of 4% buffer B (10 mM Bis–Tris-Propane pH 9.0, 1 mM MgCl_2_, 1 M NaCl). AAVs were eluted with 35% buffer B. Eluted fractions were concentrated and buffer exchanged to PBS + 0.001% (vol/vol) Poloxamer 188 using 100-kDa Amicon filters. Purity of viral preparations were assessed with SDS–PAGE/colloidal Coomassie staining and AAV titre determined using Gel green (Biotium)^116^.

### RT–qPCR analysis

RNA isolation of cerebellar tissue was performed using TRIzol (Fisher Scientific). Around 2 µg of total RNA was used for reverse transcription using the High Capacity cDNA Reverse Transcription Kit (Applied Biosystems) following the manufacturer’s instructions. qPCR was performed with PowerUp SYBR Green Master Mix (Applied Biosystems) in a StepOnePlus Real-Time PCR System (Applied Biosystems). Relative expression of gene transcripts was assessed using the 2^−ΔΔCt^ method. Primer sequences for *Slc2a2* and *Gapdh* are provided in Supplementary Table 1.

### Preparation of acute slices, starvation and RT–qPCR analysis

Cerebellar acute slices were prepared as described above. 100-µm-thick horizontal sections were either incubated in OTC medium or ACSF for 6 h at 37 °C and 5% CO_2_. Afterwards, samples were processing via RNA isolation and RT–qPCR described above.

### Stereotactic injections

Mice were weighed and anaesthetized with a mixture of ketamine (100 mg per kg body weight), xylazine (20 mg per kg body weight) and acepromazine (3 mg per kg body weight) and placed in a stereotactic frame. An eye cream was used to prevent drying of the corneas. A local painkiller was injected subcutaneously. The animal was fixed in a stereotactic frame (Kopf Instruments). The skin was opened, and the skull was cleaned using NaCl. Point injection (AP from bregma: −5.61 mm; ML: 0.0 mm; DV: −1 mm) was identified using bregma and lambda for navigation. Subsequently, a circular craniotomy was performed with a micro drill. A microinjection syringe was filled with corresponding AAV (Supplementary Table 2), and 300 nl of AAV at a speed of 100 nl min^−1^ was injected. The needle was kept in place for 3 min and slowly retracted. The incision was closed with sutures (Ethicon). During and after the surgery, mouse body temperature was kept at 37 °C via a heating pad. Mice were injected with 5% glucose–saline solution (100 μl/10 g) after the surgery and with carprofen (5 mg per kg body weight) 24 h and 48 h after the surgery. Physical conditions of the animals were monitored daily to improve their welfare. Three weeks after injection, animals were used for IHC (as described above).

### Behavioural analysis

To assess motor gait, WT, ATG5 cKO and ATG5:GLUT2 cKO mice were tasked to cross beams of 1.3 m in length and different widths (5 mm, 12 mm and 25 mm). For each mouse, three to five trials per beam were collected. Mice were recorded using eight high-speed cameras (mV Blue Cougar XD; 200 frames per second) positioned around the beam (3D SIMI Motion). Multiple camera views were analysed to count the number of slips, which were then averaged across trials per individual mouse. For the two-dimensional kinematic reconstructions of the hindlimb, we used the camera perpendicular to the beam. The iliac crest, hip, knee, ankle and hindpaw coordinates were tracked using DeepLabCut (version 2.3.5) in markerless animals. We also used DeepLabCut to label the beam position, and establish the baseline for the vertical axis. The initiation of swing, the end of swing and the end of stance were manually annotated for each step. Steps resulting in footslips were excluded from the kinematic analysis. Notably, ATG5 cKOs displayed a higher number of slips on the narrowest beam (5 mm); therefore, only three mice could be analysed in this task. We used AutoGaitA^69^ to integrate the manual annotations of individual steps with the DeepLabCut tracked coordinates.

### Statistical analysis

Sample sizes were not chosen based on prespecified effect size. Instead, multiple independent experiments were carried out using several samples replicates, as detailed in the figure legends. For all experiments, statistical power was adequate to detect the corresponding effect size. Data collection and analysis were not performed blind due to the conditions of the experiments. Data were excluded only when sample quality was not optimal or due to re‐genotyping results. Predefined quality criteria were: in living samples—vacuolization or other signs of cellular degeneration, normal cell morphology and image streams that are in focus; proper sample preparation and mounting in fixed samples. The data were tested for normality and equal variances to meet the assumptions of the statistical test. Statistical analyses were done ex vivo on cell values (indicated by data points) and/or mice for in vivo from at least three independent experiments (indicated by ‘*N*’, biological replicates). Excel (Microsoft) and GraphPad Prism version 9 (GraphPad Software) were used for statistical analysis. Values were normalized to the average of the corresponding control values to account for distribution of single experiments. Statistical analysis of normalized data between the two groups was performed using a one-tailed unpaired Student’s *t*-test. Statistical significance between two groups for normally distributed non-normalized data was evaluated with a two-tailed unpaired Student’s *t*-test. For comparisons between two groups in a set of multiple data, multiple *t*-test with post hoc correction using a two-stage linear step-up procedure of Benjamini, Krieger and Yekutieli was performed. For comparison between more than two groups, one-way ANOVA followed by a Holm–Sidak post hoc test was applied. Two-way ANOVA (or mixed-model ANOVA, if the number of *N* was not equal between conditions) was used for the comparison of two groups and two independent variables, followed by Holm–Sidak post hoc test for multiple comparisons. *P* values of less than 0.05 were considered statistically significant. Data are reported as mean values ± s.e.m.

### Inclusion and ethics statement

All contributors to this study meet the authorship criteria mandated by Nature Portfolio journals and have been credited as authors, given their indispensable involvement in shaping and executing the study. The study encompasses findings tailored to local contexts, developed in conjunction with local partners. Importantly, the research was conducted without significant constraints or prohibitions in the researchers’ environment, and safeguards against stigmatization, incrimination, discrimination or personal risks to participants were ensured. Moreover, citations acknowledge pertinent local and regional research relevant to our study.

### Reporting summary

Further information on research design is available in the Nature Portfolio Reporting Summary linked to this article.

## Supplementary information

Supplementary Information Supplementary Tables 1–4 and uncropped western blots from Figs. 1–10.

Reporting Summary

Supplementary Video 1 Kinematic analysis of WT, ATG5 cKO and ATG5:GLUT2 cKO mice while crossing 5-mm beam.

Supplementary Video 2 Kinematic analysis of WT, ATG5 cKO and ATG5:GLUT2 cKO mice while crossing 12-mm beam.

Supplementary Video 3 Kinematic analysis of WT, ATG5 cKO and ATG5:GLUT2 cKO mice while crossing 25-mm beam.

Supplementary Data 1–4 Table 1: Raw data for proteomics in Fig. 2 and Extended Data Fig. 3. Table 2: Raw data for untargeted metabolomics in Fig. 2 and Extended Data Fig. 3. Table 3: Raw data for AA content in Fig. 2. Table 4: Raw data for targeted metabolomics in Fig. 3 and Extended Data Fig. 4.

## Source data

Source Data Fig. 1 Excel file with source data and statistics.

Source Data Fig. 2 Excel file with source data and statistics.

Source Data Fig. 3 Excel file with source data and statistics.

Source Data Fig. 4 Excel file with source data and statistics.

Source Data Fig. 5 Excel file with source data and statistics.

Source Data Fig. 6 Excel file with source data and statistics.

Source Data Fig. 7 Excel file with source data and statistics.

Source Data Extended Data Fig. 1 Excel file with source data and statistics.

Source Data Extended Data Fig. 2 Excel file with source data and statistics.

Source Data Extended Data Fig. 3 Excel file with source data and statistics.

Source Data Extended Data Fig. 4 Excel file with source data and statistics.

Source Data Extended Data Fig. 5 Excel file with source data and statistics.

Source Data Extended Data Fig. 6 Excel file with source data and statistics.

Source Data Extended Data Fig. 7 Excel file with source data and statistics.

Source Data Extended Data Figure 8 Excel file with source data and statistics.

Source Data Extended Data Figure 9 Excel file with source data and statistics.

Source Data Figs. 1–7 Uncropped immunoblots Figs. 1–7.

## Data Availability

All data needed to evaluate the conclusions in the paper are present in the paper and/or the Supplementary Information. Proteome data of all experiments are deposited in the PRIDE database (ID: 3283) and are publicly accessible after publishing. Metabolomics data have been deposited in the Zenodo data repository and can be downloaded via 10.5281/zenodo.10635080 (ref. ^117^). Source data are provided with this paper. Additional data related to this paper may be requested from N.L.K.
