# Supplementary material for: Autophagy regulator ATG5 preserves cerebellar function by safeguarding its glycolytic activity
Source: Nat Metab. 2025 Jan 15;7(2):297–320. doi: 10.1038/s42255-024-01196-4 (PMC11860254; doi:10.1038/s42255-024-01196-4)
Supplement: Supplementary file 1 — Supplementary Tables 1–4 and uncropped western blots from Figs. 1–10. [file 42255_2024_1196_MOESM1_ESM.pdf]

nature metabolism

Supplementary information https://doi.org/10.1038/s42255-024-01196-4

Autophagy regulator ATG5 preserves cerebellar function by safeguarding its glycolytic activity

In the format provided by the authors and unedited

Appendix Table S1: Primers used in the current study.

Gene	Sequence (5' - 3')
Glut2	
7S	CCA ATC CCT TGG TTC ATG GTT GC
7AS	CGT AAG GCC CAA GGA AGT CCT GC
7AS Δ	CTG CTA AAG CGC ATG CTC CAG AC
Atg5	
forward1	GAA TAT GAA GCC ACA CCC CTG AAA TG
forward2	ACA ACG TCG AGC ACG CTG GCG AAG G
reverse	GTA CTG CAT AAT GGT TTA ACT CTT GC
Cre	
Cre_1	GAA CCT GAT GGA CAT GTT CAG G
Cre_2	AGT GCG TTC GAA CGC TAG AGC CTG T
Cre_3	TTA CGT CCA TCG TGG ACA
Cre_4	TGG GCT GGG TGT TAG CC
tdTomato	
tdTomato_1	AAG GGA GCT GCA GTG GAG TA
tdTomato_2	CCG AAA ATC TGT GGG AAG TC
tdTomato_3	GGC ATT AAA GCA GCG TAT CC
tdTomato_4	CTG TTC CTG TAC GGC ATG G
Glut2 (qPCR)	
forward	CCAGTACATTGCGGACTTCCTT
reverse	CTTTCCTTTGGTTTCTGGAACTTT
Gapdh (qPCR)	
forward	CCTCCAAGGAGTAAGAAACCC
reverse	GGGTGCAGCGAACTTTATTG
EGFP-hAtg5	
forward	TCGCCACCATGGTGAG
reverse	GCAGGGGTGGCGCGCCTTCAATCTGTTGGCTGTGGG

Appendix Table S2: AAVs used for transduction of neurons ex-vivo and in-vivo in the current study.

Identifier	Name	Titer [vg/ml]	Origin
ATeam	ssAAV-9/2-hSyn1-Ateam1.03YEMK-WPRE-hGHp(A)	8.1x10E12	Viral Vector Facility (VVF)
Laconic	ssAAV-9/2-hSyn1-Laconic-WPRE-hGHp(A)	6.9x10E12	Viral Vector Facility (VVF)

GCamP7f	ssAAV-9/2-mCaMKIIα-jGCaMP7f-WPRE-bGHp(A)	1.1x10E13	Viral Vector Facility (VVF)
MitoTimer	ssAAV-5/2-mTH-dlox-MitoTimer(rev)-dlox-WPRE-bGHp(A)	6.2 x 10E12	Viral Vector Facility (VVF)
EGFP-ATG5	pAAV2/RH10-mDLX-EGFP-*Atg5*	4.78 x 10E12	Custom-made
EGFP	pAAV2/RH10-mDLX-EGFP	1.31 x 10E12	Custom-made
mt-mKeima	AAV9S-Syn1-mt-mKeima	>2x1011 GC/ml	Vector Builder
mKeima-GLUT2	ssAAV2-rh10-L7-6-mKeima- *Slc2a2*-WPRE		Custom-made
mCherry-EGFP-Slc2a2	AAV9S-Syn1-mCherry-EGFP-*Slc2a2*	> 2x10E11	Vector Builder

Appendix Table S3: List of primary and secondary antibodies used in immunohistochemical studies.

**Antibody target**	**Concentration**	**Manufacturer**	**Catalog number**
Rabbit anti-BNIP3	1:300	Thermo Fisher Sci	MA5-41227
Chicken anti-Calbindin	1.500	Novus Biologicals	NBP2-50028
Rabbit anti-Cleaved Caspase-3	1:5000	Cell Signalling	9661S
Rabbit anti-D-Serine	1:250	Origene	AP02025PU-S
Mouse anti-GFAP	1:500	Sigma	G3893
Chicken anti-GFP	1:1000	Abcam	ab13970
Rabbit anti-GLUT1	1:300	Novus Biologicals	NB110-39113SS
Rabbit anti-GLUT2	1:300	Novus Biologicals	NBP2-22218SS
Rabbit anti- GLUT2	1:300	Merck	07-1402-I
Rabbit anti-GLUT3	1:300	Invitrogen	OSG00012w
Rabbit anti-GLUT4	1:300	Novus Biologicals	NBP1-4953355
Rabbit anti-Hexokinase II	1:500	Abcam	ab227198
Mouse anti-LC3	1:500	Biozol	M152-3
Rabbit anti-LC3B	1:300	Novus Biologicals	NB600-1384
Goat anti-mCathepsin D	1:300	R&D systems	AF1029
Mouse anti-Methylglyoxal	1.300	Novus Biologicals	NBP2-59368
Mouse anti-NBR1	1:300	Santa Cruz Biotechnology	sc-130380
Rabbit anti-Parvalbumin	1:500	SySy	195002
Mouse anti-Puromycin	1:1000	Merck	MABE343
Guinea pig anti-p62	1:1000	Progen	GP62-C

Mouse anti-RAB11a	1:500	Proteintech	67902
Mouse anti-RAB5	1:300	SySy	108 011
Mouse anti-TIM23	1:1000	BioSciences	611223
Mouse anti-VPS35	1:300	Santa Cruz Biotechnology	sc-374372
Alexa Fluor 488 Goat Anti-Chicken IgG	1:500	Life Technologies GmbH	A11039
Alexa Fluor 488 Goat Anti-Rabbit IgG	1:500	Life Technologies GmbH	A11034
Alexa Fluor 488 Goat Anti-Guinea Pig IgG	1:500	Life Technologies GmbH	A11073
Alexa Fluor 488 Goat Anti-Mouse IgG	1:500	Life Technologies GmbH	A11029
Alexa Fluor™ 488 Donkey Anti-Mouse	1:500	Life Technologies GmbH	A32766
Alexa Fluor 568 donkey anti-goat IgG	1:500	Life Technologies GmbH	A10057
Alexa Fluor 568 goat anti-rabbit IgG	1:500	Life Technologies GmbH	A11011
Alexa Fluor 647 Donkey Anti-Rabbit IgG	1:500	Life Technologies GmbH	A31573
Alexa Fluor 647 Goat Anti-Guinea Pig IgG	1:500	Life Technologies GmbH	A21450
Alexa Fluor 647 Goat Anti-Rabbit IgG	1:500	Life Technologies GmbH	A21245
Alexa Fluor 647 Goat Anti-Mouse IgG	1:500	Life Technologies GmbH	A21236

Appendix Table S4: List of primary and secondary antibodies used in immunoblotting studies.

**Antibody target**	**Dilution**	**Manufacturer**	**Catalog number**
Mouse anti-AMPKα	1:1000	Abcam	Ab80039
Mouse anti-AMPKα (phospho-Thr142)	1:1000	Cell Signalling	2535S
Rabbit anti-ATG5	1:1000	Abcam	ab108327
Mouse anti-α-Tubulin	1:5000	Synaptic Systems	302 211
Mouse anti-β-Actin	1:3000	Sigma	A-5441
Rabbit anti-BNIP3	1:1000	Thermo Fisher Sci	MA5-41227
Chicken anti-Calbindin	1:1000	Novus Biologicals	NBP2-50028
Mouse anti-eCadherin	1:1000	BD Biosciences	610182
Mouse anti-GFAP	1:1000	Sigma	G3893
Rabbit anti-GLUT2	1:500	Novus Biologicals	NBP2-22218SS

Rabbit anti-GLUT2	1:300	AdipoGen	AG-25B-0042-C050
Rabbit anti-LC3B	1:1000	Novus Biologicals	NB600-1384
Mouse anti-Methylglyoxal	1:1000	Novus Biologicals	NBP2-59368
Mouse anti-NeuN	1:1000	Abcam	ab104224
Guinea pig anti-p62	1:1000	Progen	GP62-C
Rabbit anti-Vinculin	1:5000	Abcam	ab129002
Rabbit anti-Mouse IgG (H+L) peroxidase-conjugated	1:10 000	Sigma	A9044
Goat anti-Rabbit IgG (H+L) peroxidase-conjugated	1:10 000	Sigma	A0545
Goat anti-Guinea Pig IgG (H+L) peroxidase-conjugated	1:5 000	Jackson Immuno Research	106-035-003
Rabbit anti-Chicken IgG (H+L) peroxidase-conjugated	1:5 000	Millipore	AP162P

Figure S1

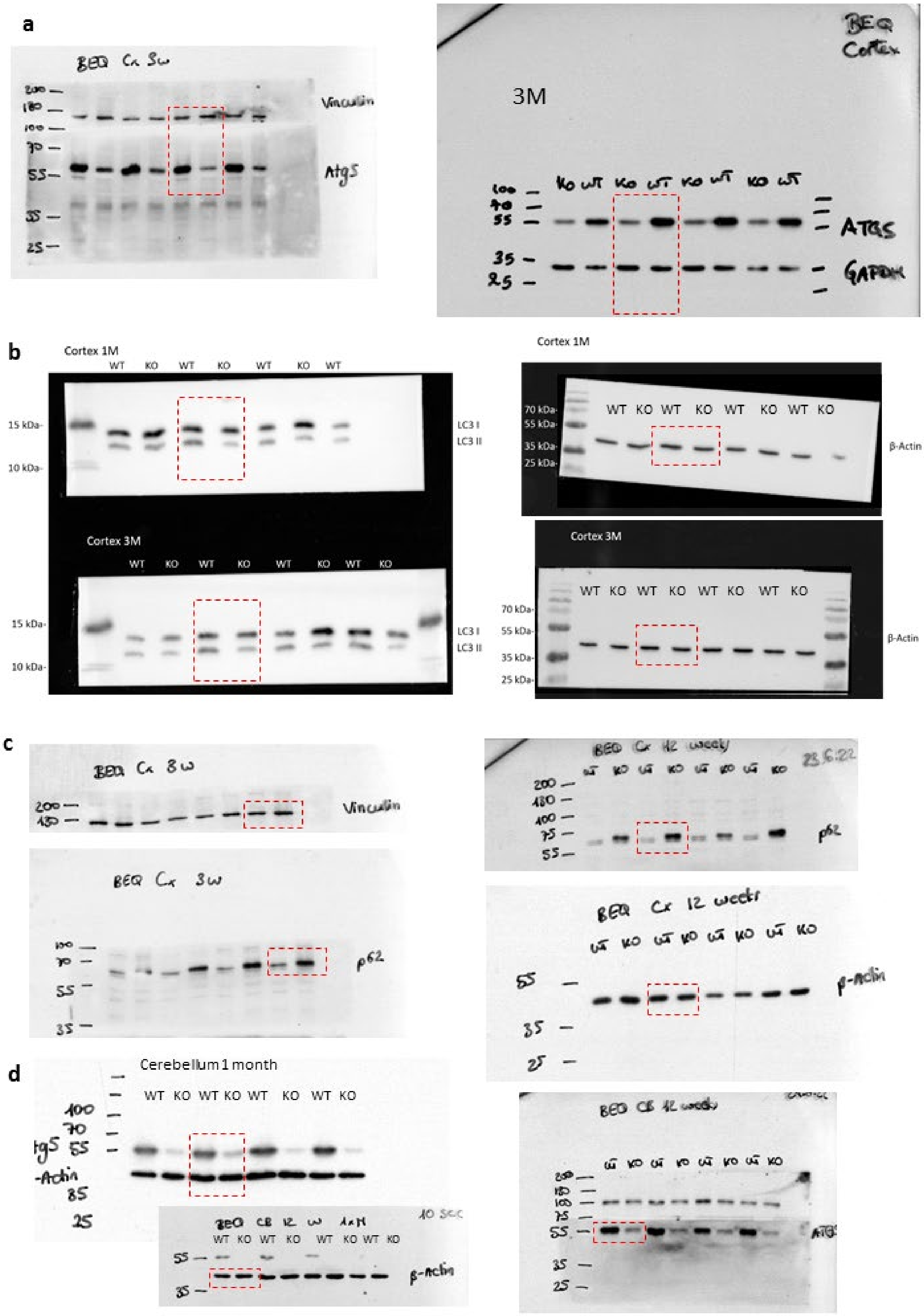
a
BEQ Cx 3w
Vinculin
Atg5
3M
BEQ Cortex
KO WT KO WT KO WT KO WT
ATG5
GAPDH
b
Cortex 1M
WT KO WT KO WT KO WT
15 kDa-
10 kDa-
LC3 I
LC3 II
Cortex 1M
WT KO WT KO WT KO WT KO
70 kDa-
55 kDa-
35 kDa-
25 kDa-
β-Actin
Cortex 3M
WT KO WT KO WT KO WT KO
15 kDa-
10 kDa-
LC3 I
LC3 II
Cortex 3M
WT KO WT KO WT KO WT KO
70 kDa-
55 kDa-
35 kDa-
25 kDa-
β-Actin
c
BEQ Cx 8w
Vinculin
BEQ Cx 3w
p62
BEQ Cx 12 weeks
p62
BEQ Cx 12 weeks
WT KO WT KO WT KO WT KO
β-actin
d
Cerebellum 1 month
WT KO WT KO WT KO WT KO
BEQ CB 12 w
WT KO WT KO WT KO WT KO
β-actin
BEQ CB 12 weeks
WT KO WT KO WT KO WT KO
Atg5

Figure S1

e

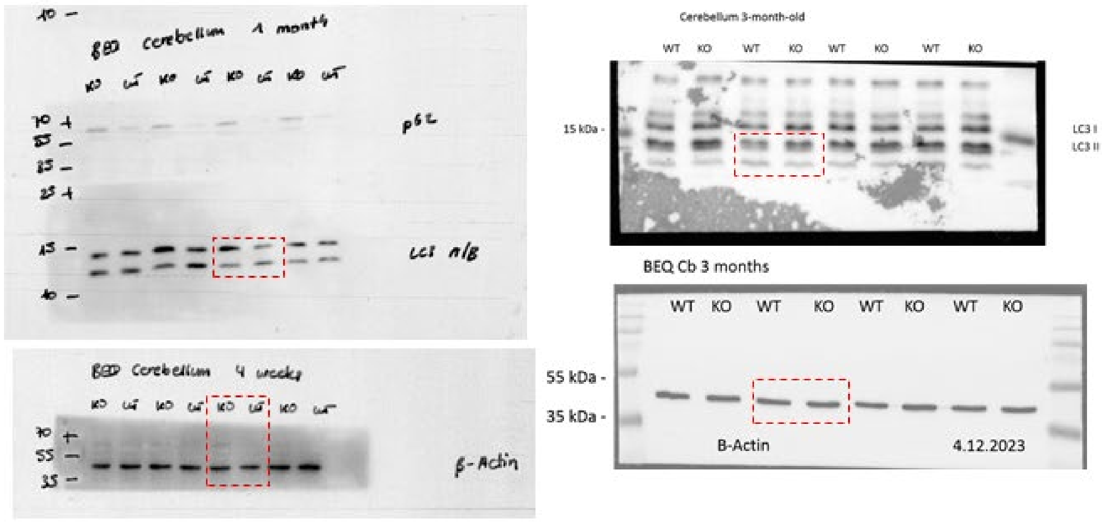

f

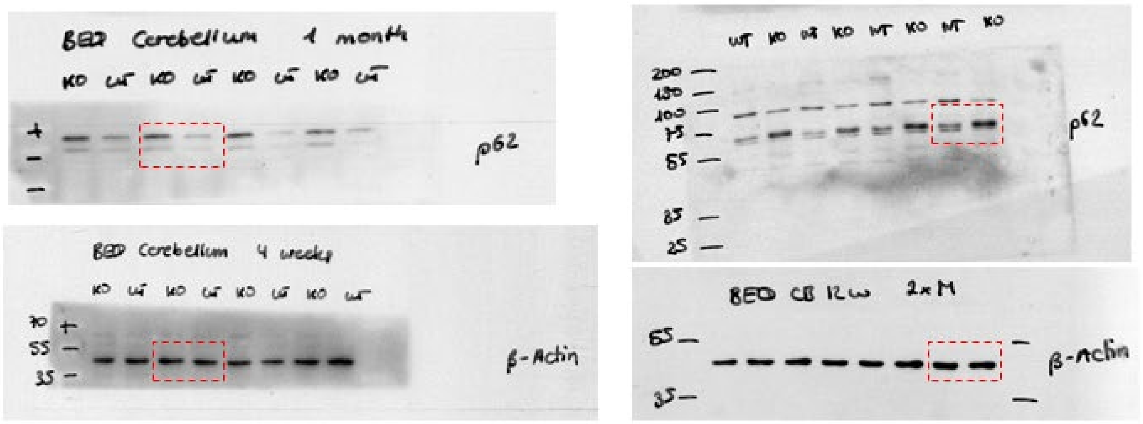

g

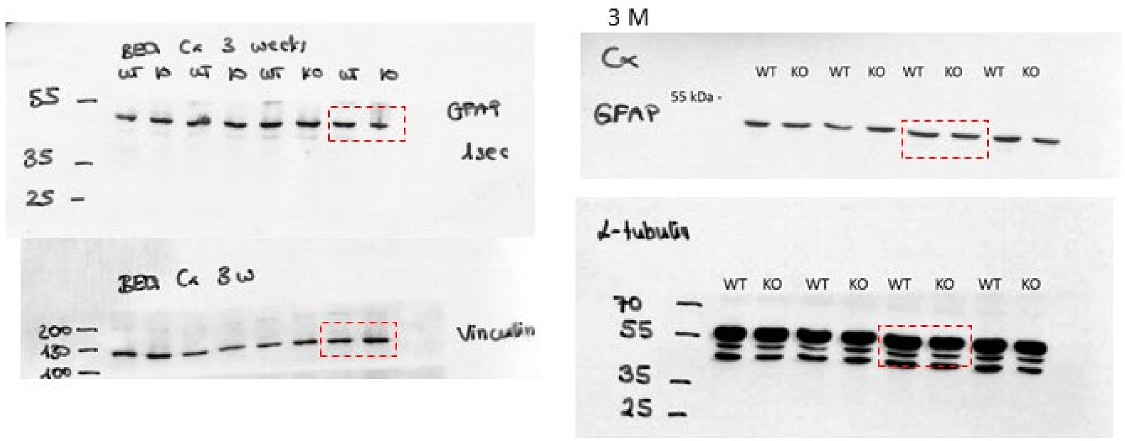

Figure S1

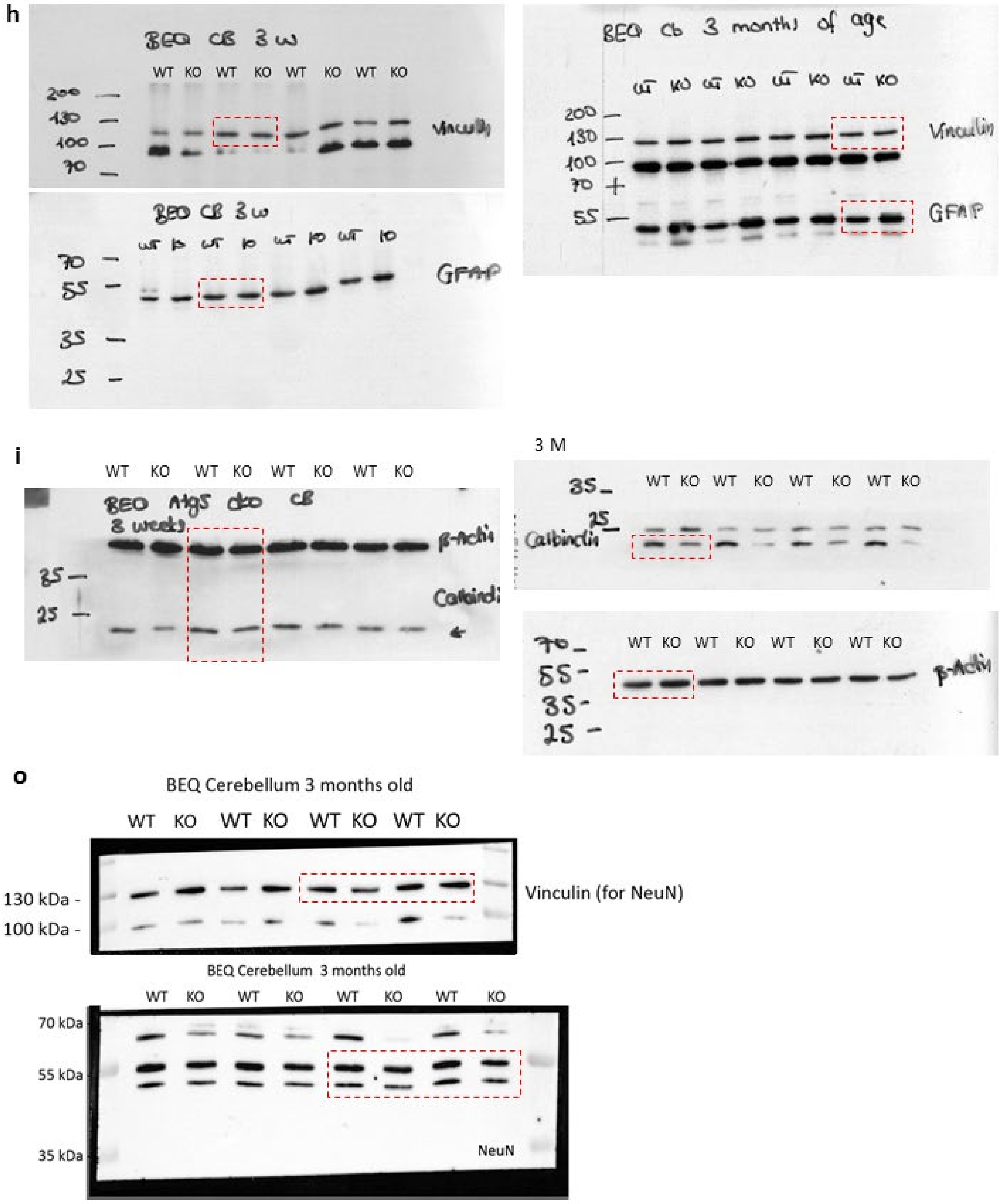
h
BEQ CB 3 w
WT KO WT KO WT KO WT KO
200
130
100
70
Vinculin
BEQ CB 3 w
70
55
35
25
GFAP
BEQ Cb 3 months of age
200
130
100
70
55
Vinculin
GFAP
i
WT KO WT KO WT KO WT KO
β-Actin
35
25
Calbindi
3 M
35
25
Calbindin
WT KO WT KO WT KO WT KO
70
55
35
25
β-Actin
o
BEQ Cerebellum 3 months old
WT KO WT KO WT KO WT KO
130 kDa -
100 kDa -
Vinculin (for NeuN)
BEQ Cerebellum 3 months old
WT KO WT KO WT KO WT KO
70 kDa
55 kDa
35 kDa
NeuN

Figure S2

s

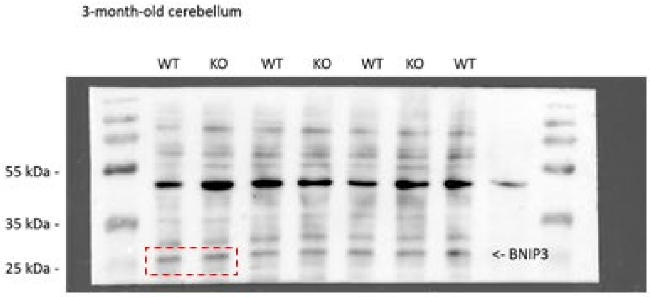
3-month-old cerebellum
WT KO WT KO WT KO WT
55 kDa -
35 kDa -
25 kDa -
<- BNIP3

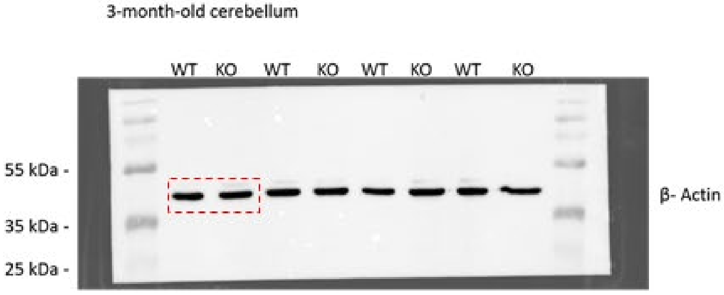
3-month-old cerebellum
WT KO WT KO WT KO WT KO
55 kDa -
35 kDa -
25 kDa -
β- Actin

Figure S5

d

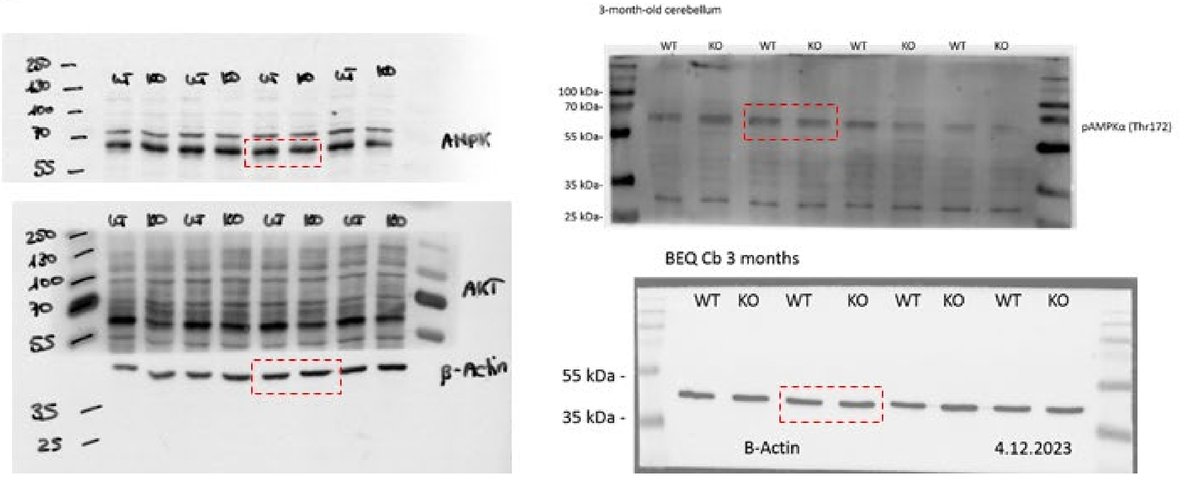
WT KO WT KO WT KO WT KO
250
130
100
70
55
AMPK
AKT
β-Actin
35
25
3-month-old cerebellum
100 kDa-
70 kDa-
55 kDa-
35 kDa-
25 kDa-
pAMPKα (Thr172)
BEQ Cb 3 months
55 kDa -
35 kDa -
B-Actin
4.12.2023

o

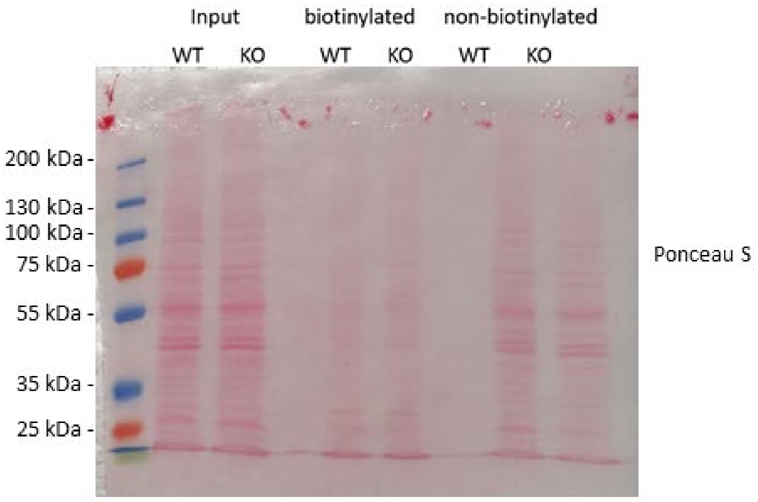
Input
biotinylated
non-biotinylated
WT KO WT KO WT KO
200 kDa -
130 kDa -
100 kDa -
75 kDa -
55 kDa -
35 kDa -
25 kDa -
Ponceau S

Figure S7

c

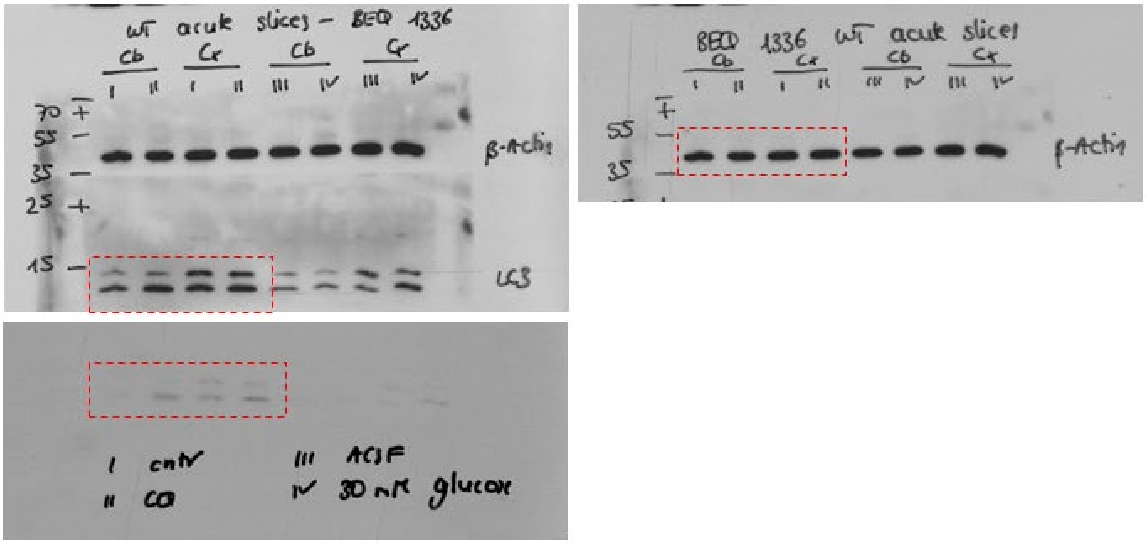
WT acute slices – BEQ 1336
Cb
Cr
Cb
Cr
I II I II III IV III IV
70
55
35
25
15
β-Actin
LC3
I cntr
II CQ
III ACSF
IV 30 mM glucose
BEQ 1336 WT acute slices
Cb
Cr
Cb
Cr
I II I II III IV III IV
55
35
β-Actin

f

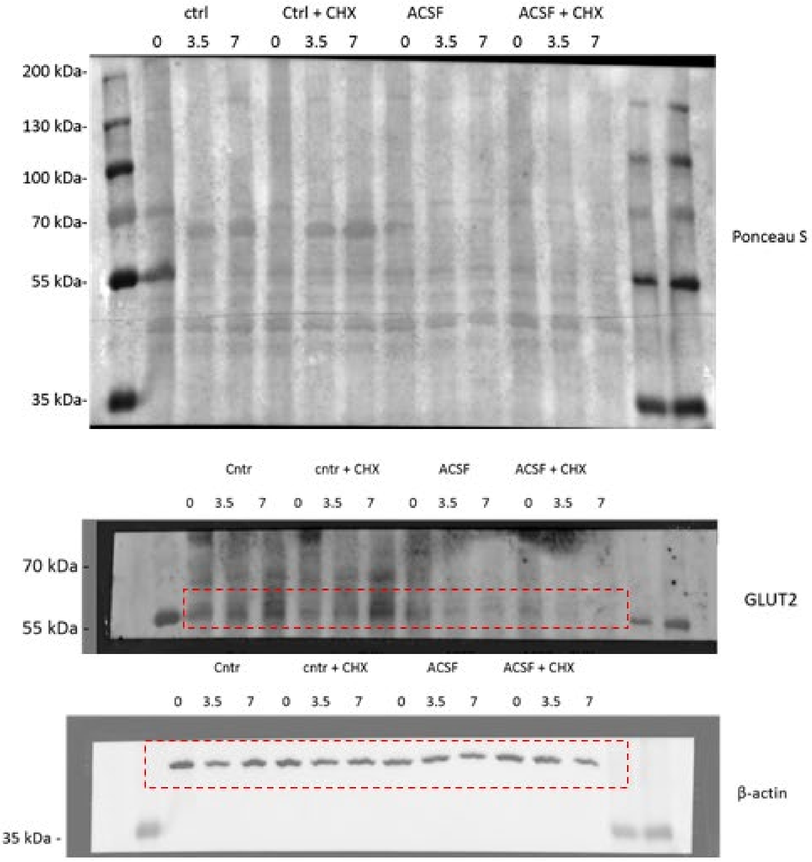
ctrl
Ctrl + CHX
ACSF
ACSF + CHX
0 3.5 7 0 3.5 7 0 3.5 7 0 3.5 7
200 kDa-
130 kDa-
100 kDa-
70 kDa-
55 kDa-
35 kDa-
Ponceau S
Cntr
cntr + CHX
ACSF
ACSF + CHX
0 3.5 7 0 3.5 7 0 3.5 7 0 3.5 7
70 kDa
55 kDa
GLUT2
Cntr
cntr + CHX
ACSF
ACSF + CHX
0 3.5 7 0 3.5 7 0 3.5 7 0 3.5 7
35 kDa -
β-actin

Figure S9

a

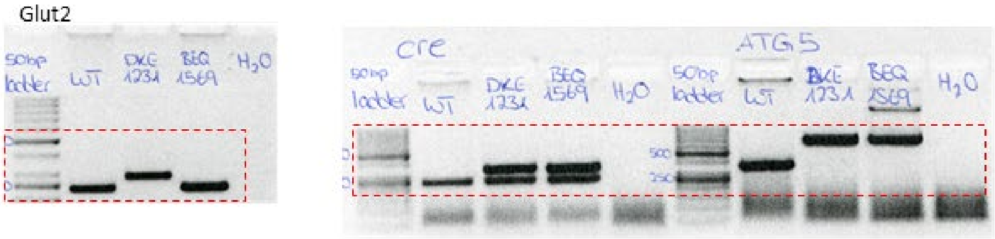
Glut2
50bp ladder
WT
DKE 1731
BEQ 1569
H2O
Cre
ATG5
50bp ladder
WT
DKE 1731
BEQ 1569
H2O
50bp ladder
WT
BKE 1731
BEQ 1569
H2O

g

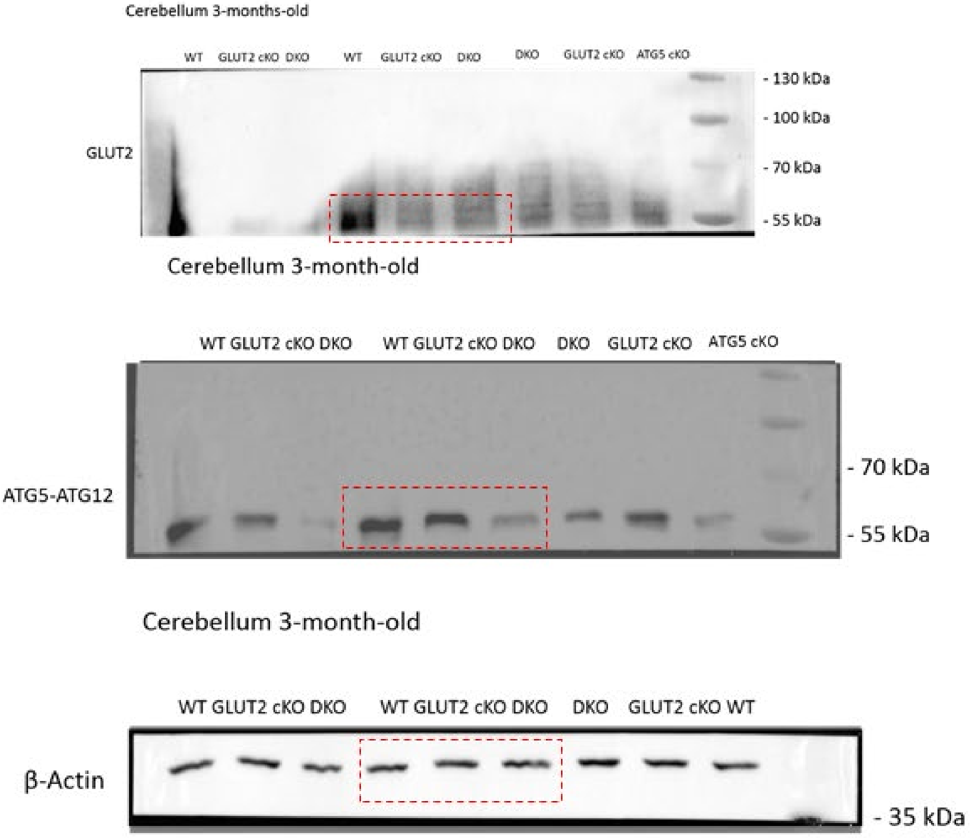
Cerebellum 3-months-old
WT GLUT2 cKO DKO WT GLUT2 cKO DKO DKO GLUT2 cKO ATG5 cKO
GLUT2
- 130 kDa
- 100 kDa
- 70 kDa
- 55 kDa
Cerebellum 3-month-old
WT GLUT2 cKO DKO WT GLUT2 cKO DKO DKO GLUT2 cKO ATG5 cKO
ATG5-ATG12
- 70 kDa
- 55 kDa
Cerebellum 3-month-old
WT GLUT2 cKO DKO WT GLUT2 cKO DKO DKO GLUT2 cKO WT
β-Actin
- 35 kDa
