# Supplementary material for: Autophagy regulator ATG5 preserves cerebellar function by safeguarding its glycolytic activity
Source: Nat Metab. 2025 Jan 15;7(2):297–320. doi: 10.1038/s42255-024-01196-4 (PMC11860254; doi:10.1038/s42255-024-01196-4)
Supplement: Supplementary file 2 — Reporting Summary [file 42255_2024_1196_MOESM2_ESM.pdf]

nature portfolio

Corresponding author(s): Natalia Kononenko

Last updated by author(s): Oct 28, 2024

Reporting Summary

Nature Portfolio wishes to improve the reproducibility of the work that we publish. This form provides structure for consistency and transparency in reporting. For further information on Nature Portfolio policies, see our Editorial Policies and the Editorial Policy Checklist.

Statistics

For all statistical analyses, confirm that the following items are present in the figure legend, table legend, main text, or Methods section.

n/a	Confirmed	
☐	☒	The exact sample size (*n*) for each experimental group/condition, given as a discrete number and unit of measurement
☐	☒	A statement on whether measurements were taken from distinct samples or whether the same sample was measured repeatedly
☐	☒	The statistical test(s) used AND whether they are one- or two-sided<br>*Only common tests should be described solely by name; describe more complex techniques in the Methods section.*
☒	☐	A description of all covariates tested
☐	☒	A description of any assumptions or corrections, such as tests of normality and adjustment for multiple comparisons
☐	☒	A full description of the statistical parameters including central tendency (e.g. means) or other basic estimates (e.g. regression coefficient) AND variation (e.g. standard deviation) or associated estimates of uncertainty (e.g. confidence intervals)
☐	☒	For null hypothesis testing, the test statistic (e.g. *F*, *t*, *r*) with confidence intervals, effect sizes, degrees of freedom and *P* value noted<br>*Give P values as exact values whenever suitable.*
☒	☐	For Bayesian analysis, information on the choice of priors and Markov chain Monte Carlo settings
☒	☐	For hierarchical and complex designs, identification of the appropriate level for tests and full reporting of outcomes
☐	☒	Estimates of effect sizes (e.g. Cohen's *d*, Pearson's *r*), indicating how they were calculated

Our web collection on statistics for biologists contains articles on many of the points above.

Software and code

Policy information about availability of computer code

Data collection	Seahorse XF96 analyzer; Focus 220 micro PET scanner 597 (CTI-Siemens, Erlangen, Germany); Vanquish Horizon UHPLC connected to an Orbitrap 746 Exploris 240 mass spectrometer (Thermo Fisher Scientific); Anion exchange chromatography (Integrion, Thermo Fisher Scientific), coupled to high resolution mass spectrometry (Q-Exactive HF,Thermo Fisher Scientific); Dionex Integrion RFIC system (Thermo Scientific) coupled to a Q Exactive HF quadrupole-orbitrap mass spectrometer (Thermo Scientific); Orbitrap Exploris 480 (Thermo Scientific); S360 Hamamatsu slide scanner; JEM-2100 Plus Transmission Electron Microscope (JEOL, Tokio, Japan); ECL-based autoradiography film system (Super RX-N, Fujifilm); ChemiDocTM Imaging system (BioRad); StepOnePlusTM Real-Time PCR System (Applied Biosystems); LAS X 3.5.7.23225; LAS X 4.5.0.25531. DeepLabCut 2.3.5, AutoGaitA (Hosseini et al., 2024), Seahorse WAVE Controller Software 2.6.1 (Agilent), TraceFinder software (Version 5.1 and Version 5.0, Thermo Fisher Scientific), Compound Discoverer software (v 3.2, Thermo Fisher Scientific), StepOnePlusTM Real-Time PCR System (Applied Biosystems), Compound Discoverer v3.2, Skyline 21.2.0.369
Data analysis	Seahorse WAVE Controller Software 2.6.1 (Agilent); VINCI 5.21 (PET) for MacOS X; FIJI ImageJ 1.53u; Compund Discoverer software (v 3.2, Thermo Fisher; Tracefinder software (v 5.0 and v5.1, Thermo Fisher Scientific). Scientific); MetaboAnalyst 5.0; GraphPad Prism 9.5.1; Nexera X2 UHPLC System (Shimadzu) coupled to a QTRAP 6500 triple quadrupole/linear ion trap mass spectrometer (SCIEX); MultiQuant 3.0.2 software (SCIEX); DIA-NN 1.8.1; Perseus 1.6.15; ShinyGO 0.81 (South 927 Dakota State University); Venny2.1; Amira Software 2020.2 (Thermo Fisher Scientific); Aperio ImageScope (Leica, version 12.4.3.5008); Microsoft Excel 2016; 3D SIMI Motion; AutoGaitA (Hosseini et al., 2024), RStudio 1.0.153 for MaxOS X, Instant Clue v0.11.3, Excel 2016, ImageJ 1.53u (Fiji), Aperio Image Scope 12.4.3.5008, Amira Software 2020.2 (Thermo Fisher Scientific), Perseus 1.6.15, R Version 4.1.3, DIA-NN 1.8.1 (Demichev 2020)

For manuscripts utilizing custom algorithms or software that are central to the research but not yet described in published literature, software must be made available to editors and reviewers. We strongly encourage code deposition in a community repository (e.g. GitHub). See the Nature Portfolio guidelines for submitting code & software for further information.

Data

Policy information about availability of data

All manuscripts must include a data availability statement. This statement should provide the following information, where applicable:

- Accession codes, unique identifiers, or web links for publicly available datasets
- A description of any restrictions on data availability
- For clinical datasets or third party data, please ensure that the statement adheres to our policy

All quantitative analyses performed in this study are provided with the manuscript and/or a Source Data file (excel files provided for each main and suppl. figure). Immunoblots will be provided as uncropped films. Proteome and metabolome data of all experiments are provided as Tables EV1-4. Metabolome data deposited in the database Zenodo (https://zenodo.org/uploads/10635080) and accessible for public after publishing. Proteomics data is deposided in PRIDE databsase (PRIDE (ID: 3283) and will be accesible after publishing. Additional data related to this paper may be requested from the corresponding author.

Research involving human participants, their data, or biological material

Policy information about studies with human participants or human data. See also policy information about sex, gender (identity/presentation), and sexual orientation and race, ethnicity and racism.

Reporting on sex and gender	n.a.
Reporting on race, ethnicity, or other socially relevant groupings	n.a.
Population characteristics	n.a.
Recruitment	n.a.
Ethics oversight	n.a.

Note that full information on the approval of the study protocol must also be provided in the manuscript.

Field-specific reporting

Please select the one below that is the best fit for your research. If you are not sure, read the appropriate sections before making your selection.

☒ Life sciences ☐ Behavioural & social sciences ☐ Ecological, evolutionary & environmental sciences

For a reference copy of the document with all sections, see nature.com/documents/nr-reporting-summary-flat.pdf

Life sciences study design

All studies must disclose on these points even when the disclosure is negative.

Sample size	Sample sizes were not chosen based on pre-specified effect size. Instead, multiple independent experiments were carried out using several samples replicates, as detailed in the figure legends. For all experiments, there was enough statistical power to detect the corresponding effect size. Sample sizes were chosen based on previos publications (Overhoff et al., 2022, Negrete-Hurtado et al., 2020).
Data exclusions	Data was only excluded when the quality of the sample was not optimal or due to re-genotyping results. Predefined quality criteria were: in living samples- vacuolization orother signs of cellular degeneration, normal cell morphology and protein transport (which is usually hampered inunhealthy cells) and image streams that are in focus; proper sample preparation and mounting in fixed samples.
Replication	Each experiment was replicated a minimum of three times and data was reliably reproduced with each replication attempt.
Randomization	Mice were genotyped for allocation into control or experimental groups. Within each group, all animals were generally used for experimental procedures, so no randomization was applied. Covariates were controlled by using litter mates.
Blinding	Animals were genotyped prior to experiments, i.e. no blinding was used to allocate experimental groups. Immunofluorescence image data collection and analysis was not blinded since KO phenotype differentiated from controls. Data analysis was performed blinded whenever possible. Western blot data were not blindly analysed since samples came from WT and KO animals. Blinding was not applicable since phenotype of the animals were clear to distinguish between WT and KO, even though genotype was not known.

Reporting for specific materials, systems and methods

We require information from authors about some types of materials, experimental systems and methods used in many studies. Here, indicate whether each material, system or method listed is relevant to your study. If you are not sure if a list item applies to your research, read the appropriate section before selecting a response.

Materials & experimental systems

n/a	Involved in the study
☐	☒ Antibodies
☐	☒ Eukaryotic cell lines
☒	☐ Palaeontology and archaeology
☐	☒ Animals and other organisms
☒	☐ Clinical data
☒	☐ Dual use research of concern
☒	☐ Plants

Methods

n/a	Involved in the study
☒	☐ ChIP-seq
☒	☐ Flow cytometry
☒	☐ MRI-based neuroimaging

Antibodies

Antibodies used

Rabbit polyclonal anti-CASPASE-3 cleaved (Asp175) Cell Signaling # 9661
Recombinant Rabbit Monoclonal BNIP3 Themo Fisher Sie #MA5-41227 clone JA71-10
Chicken polyclonal anti- Calbindin (D-28K) Novus Biological #NBP2-50028
Rabbit polyclonal anti-GLUT2 Merck #07-1402-I
Mouse monoclonal anti-GFAP Sigma #G3893 clone G-A-5
Chicken polyclonal anti-GFP Abcam #ab13970
Rabbit polyclonal anti-GLUT1 Novus Biological #NB110-39113
Rabbit polyclonal anti-GLUT2 Novus Biological #NB22218
Rabbit polyclonal anti-GLUT3 Invitrogen #OSG00012W
Rabbit polyclonal anti-GLUT4 Novus Biological #NBP1-49533
Rabbit polyclonal anti-Hexokinase II Abcam #ab227198
Mouse monoclonal anti-LC3 Biozol #M152-3 clone 4E12
Mouse polyclonal anti-Cathepsin D R&D Systems #AF1029
Mouse monoclonal anti-Methylgloxal Novus Biologicals #NBP2-59368 clone 9E7
Mouse monoclonal anti-NBR1 Santa Cruz Biotechnology #sc-130380 clone 4BR
Rabbit polyclonal anti-Parvalbumin SySy #195002
Guinea pig polyclonal anti-p62 (SQSTM1) Progen #GP62-C
Mouse monoclonal anti-Tim23 BD Biosciences #611223 clone 32/Tim23 (RUO)
Rabbit polyclonal anti-Ubiquitin Sigma #U5379
Alexa Fluor 488 Goat anti-Chicken IgG Thermo Fisher Sci Cat# A-11039
Alexa Fluor 488 Goat anti-Mouse IgG Thermo Fisher Sci Cat# A-11029
Alexa Fluor 488 Goat anti-Rabbit IgG Thermo Fisher Sci # A-11034
Alexa Fluor 488 Donkey anti-Mouse IgG Thermo Fisher Sci # A-32766
Alexa Fluor 488 Goat anti-Guinea Pig IgG Thermo Fisher Sci # A-11073
Alexa Fluor 568 Goat anti-Rabbit IgG Thermo Fisher Sci Cat# A-11011
Alexa Fluor 647 Donkey anti-Rabbit Thermo Fisher Sci #A-31573
Alexa Fluor 647 Goat anti-Guinea Pig IgG Thermo Fisher Sci # A-21450
Alexa Fluor 647 Goat anti-Rabbit IgG Thermo Fisher Sci # A-21245
Alexa Fluor 647 Goat anti-Mouse IgG Thermo Fisher Sci # A-21236
Mouse monoclonal anti-AMPK (alpha 1 & alpha 2) Abcam #ab80039 clone 34.2
Rabbit monoclonal anti-phospho-AMPK (Thr172). Cell Signalling #2535 clone 40H9
Rabbit monoclonal anti-ATG5 Abcam # ab108327 clone EPR1755(2)
Mouse monoclonal anti-α-Tubulin (clone 3A2) Synaptic Systems # 302 211 clone 3A2
Mouse monoclonal anti-β-Actin Sigma #A5441 clone AC-15
Rabbit polyclonal anti-GLUT2 ProteinTech #20436-1-AP
Rabbit polyclonal anti-LC3B Novus Biologicals # NB600-1384
Rabbit monoclonal anti-Vinculin Abcam #ab129002 clone EPR8185
Rabbit polyclonal anti-Mouse IgG (H+L) peroxidase-conjugated Sigma #A9044
Goat polyclonal anti-Rabbit IgG (H+L) peroxidase-conjugatedSigma #A0545
Goat polyclonal anti-Guinea Pig IgG (H+L) peroxidase-conjugated Jackson ImmunoResearch # 106-035-003
Rabbit polyclonal anti-Chicken IgG (H+L) peroxidase-conjugated Millipore #AP162P
Mouse monoclonal anti-Puromycin Merck #MABE343 clone 12D10
Mouse monoclonal anti-RAB11A Proteintech #67902-1-Ig clone 4A4C9
Mouse monoclonal anti-RAB5 SySy # 108 011 clone 621.3
Mouse monoclonal anti-VPS35 Santa cruz #sc-374372 clone B-5
Purified mouse anti- eCadherin BD Biosciences #610182 clone 36/E-Cadherin (RUO)
Mouse polyclonal anti-GLUT2 AdipoGen #AG-25B-0042-C050
Mouse monoclonal anti-NeuN Abcam #ab104224 clone 1B7
Rabbit anti-D-Serine Origene #AP02025PU-S

Validation

Rabbit polyclonal anti-CASPASE-3 cleaved (Asp175) Cell Signaling Cat# 9661: manufacturer validation (https://www.cellsignal.com/products/primary-antibodies/cleaved-caspase-3-asp175-antibody/9661). Liu, Z., Wang, X., et al. TNFα-induced Up-regulation of Ascl2 Affects the Differentiation and Proliferation of Neural Stem Cells. Aging ans Disease (2019). Tested in IHC in mouse tissue. Lachance, V., Wang, Q., et al. Autophagy protein NRBF2 has reduced expression in Alzheimer's brains and modulates memory and amyloid-beta

homeostasis in mice. Mol Neurodegeneration (2019). Tested by WB in brain mouse samples.

Recombinant Rabbit Monoclonal BNIP3 Themo Fisher Sie #MA5-41227: manufacturer validation (https://www.thermofisher.com/antibody/product/BNIP3-Antibody-clone-JA71-10-Recombinant-Monoclonal/MA5-41227).

Chicken polyclonal anti- Calbindin (D-28K) Novus Biological #NBP2-50028: manufacturer validation (https://www.novusbio.com/products/calbindin-d-28k-antibody_nbp2-50028) . Chen et al., Loss of Flot2 expression in deep cerebellar nuclei neurons of mice with Niemann-Pick disease type C Heliyon (2023). Tested in IHC in mouse brain tissue. Jiang et al. Neuronal signal-regulatory protein alpha drives microglial phagocytosis by limiting microglial interaction with CD47 in the retina (2022. Tested in IHC in mouse tissue.

Rabbit polyclonal anit-GLUT2 Merck #07-1402-I. manufacturer validation (https://www.merckmillipore.com/DE/de/product/Anti-GLUT-2,MM_NF-07-1402-I).

Mouse monoclonal anti-GFAP Sigma #G3893. manufacturer validation (https://www.sigmaaldrich.com/DE/en/product/sigma/g3893).

Chicken polyclonal anti-GFP Abcam #ab13970. manufacturer validation (https://www.abcam.com/en-de/products/primary-antibodies/gfp-antibody-ab13970). Blanco et al., Stem cell function and stress response are controlled by protein synthesis (2022). Tested in IHC. Armbruster et al., Glutamate Clearance Is Locally Modulated by Presynaptic Neuronal Activity in the Cerebral Cortex (2022). Tested in IHC in mouse tissue.

Rabbit polyclonal anti-GLUT1 Novus Biological #NB110-39113 . manufacturer validation (https://www.novusbio.com/products/glut1-antibody_nb110-39113). Jacobs et al., Cluster analysis of DCE-MRI data identifies regional tracer-kinetic changes after tumor treatment with high intensity focused ultrasound (2015). Tested in IHC in mouse tissue.

Rabbit polyclonal anti-GLUT2 Novus Biological #NB22218. manufacturer validation (https://www.novusbio.com/products/glut2-antibody_nbp2-22218). Yang et al., Reduction of mRNA m6A associates with glucose metabolism via YTHDC1 in human and mice (2023). Tested in WB in mouse tissue. Juras et al., In situ microwave fixation provides an instantaneous snapshot of the brain metabolome (2023). Tested in IHC in mouse tissue.

Rabbit polyclonal anti-GLUT3 Invitrogen #OSG00012W. manufacturer validation (https://www.thermofisher.com/antibody/product/SLC2A3-Antibody-Polyclonal/OSG00012W-100UL). Nagamatsu et al., Neuron-specific glucose transporter (NSGT): CNS distribution of GLUT3 rat glucose transporter (RGT3) in rat central neurons (1993).

Rabbit polyclonal anti-GLUT4 Novus Biological #NBP1-49533. manufacturer validation (https://www.novusbio.com/products/glut4-antibody_nbp1-49533). Gauger et al., Mice deficient in Sfrp1 exhibit increased adiposity, dysregulated glucose metabolism, and enhanced macrophage infiltration (2013). Tested in IHC in mouse tissue.

Rabbit polyclonal anti-Hexokinase II Abcam #ab227198. manufacturer validation (https://www.abcam.com/en-de/products/primary-antibodies/hexokinase-ii-antibody-ab227198). Xiaoyan et al., Circular RNA circSEC24A Promotes Cutaneous Squamous Cell Carcinoma Progression by Regulating miR-1193/MAP3K9 Axis (2021).

Mouse monoclonal anit-LC3 Biozol #M152-3. manufacturer validation (https://www.biozol.de/en/product/mbl-m152-3). Chalazonitis et al. Homeodomain interacting protein kinase 2 regulates postnatal development of enteric dopaminergic neurons and glia via BMP signaling (2011). Tested in IHC in mouse tissue. Nakano et al. The role of p62/SQSTM1 in sporadic inclusion body myositis (2017). Tested in IHC in mouse tissue.

Mouse polyclonal anti-Cathepsin D R&D Systems #AF1029. manufacturer validation (https://www.rndsystems.com/products/mouse-cathepsin-d-antibody_af1029). Jung et al., Anti-inflammatory clearance of amyloid-beta by a chimeric Gas6 fusion protein (2022). Tested in IHC in mouse tissue. Lee et al., Astrocytes phagocytose adult hippocampal synapses for circuit homeostasis (2020). Tested in IHC in mouse tissue.

Mouse monoclonal anti-Methylgloxal Novus Biologicals #NBP2-59368. manufacturer validation (https://www.novusbio.com/products/methylglyoxal-antibody-9e7_nbp2-59368).

Mouse monoclonal anti-NBR1 Santa Cruz Biotechnology #sc-130380. manufacturer validation (https://www.scbt.com/de/p/nbr1-antibody-4br). Whitehouse et al., Brca1 expression is regulated by a bidirectional promoter that is shared by the Nbr1 gene in mouse (2004). Chen et al. Sequential barriers and an obligatory metastable intermediate define the apparent two-state folding pathway of the ubiquitin-like PB1 domain of NBR1 (2008).

Rabbit polyclonal anti-Parvalbumin SySy #195002. manufacturer validation (https://sysy.com/product/195002). Wang et al., Loss of the parkinsonism-associated protein FBXO7 in glutamatergic forebrain neurons in mice leads to abnormal motor behavior and synaptic defect (2023). Tested in IHC in mouse tissue. Schulz and Richter, In vivo optogenetic inhibition of striatal parvalbumin-reactive interneurons induced genotype-specific changes in neuronal activity without dystonic signs in male DYT1 knock-in mice (2023). Tested in IHC in mouse tissue.

Guinea pig polyclonal anti-p62 (SQSTM1) Progen #GP62-C. manufacturer validation (https://www.progen.com/anti-p62-SQSTM1-C-terminus-guinea-pig-polyclonal-serum/GP62-C). Akwa et al., Stimulation of synaptic activity promotes TFEB-mediated clearance of pathological MAPT/Tau in cellular and mouse models of tauopathies (2023). Tested in IHC in mouse tissue. Suzuki et al. Lack of Cathepsin D in the central nervous system results in microglia and astrocyte activation and the accumulation of proteinopathy-related proteins (2022). Tested in IHC in mouse tissue.

Mouse monoclonal anti-Tim23 BD Biosciences #611223. manufacturer validation (https://www.bdbiosciences.com/en-eu/products/reagents/microscopy-imaging-reagents/immunofluorescence-reagents/purified-mouse-anti-tim23.611223/). Moro et al., The TIM17.23 preprotein translocase of mitochondria: composition and function in protein transport into the matrix (1999). Rassow et al., The preprotein translocase of the mitochondrial inner membrane: function and evolution (1999).

Rabbit polyclonal anti-Ubiquitin Sigma #U5379. manufacturer validation (https://www.sigmaaldrich.com/DE/en/product/sigma/u5379). Kertesz et al., The evolution and pathology of frontotemporal dementia (2005).

llexa Fluor 488 Goat anti-Chicken IgG Thermo Fisher Sci Cat# A-11039: manufacturer validation (https:/7www.thermofisher.com/antibody/product/Goat-anti-Chicken-IgY-H-L-Secondary-Antibody-Polyclonal/A-11039). Quadrato, G., Nguyen, T., Macosko, E. et al. Cell diversity and network dynamics in photosensitive human brain organoids. Nature 545, 48–53 (2017).

Alexa Fluor 488 Goat anti-Mouse IgG Thermo Fisher Sci Cat# A-11029: manufacturer validation (https://www.thermofisher.com/antibody/product/Goat-anti-Mouse-IgG-H-L-Highly-Cross-Adsorbed-Secondary-Antibody-Polyclonal/A-11029). Shumilov, A., Tsai, M., Schlosser, Y. et al. Epstein–Barr virus particles induce centrosome amplification and chromosomal instability. Nat Commun 8, 14257 (2017).

Alexa Fluor 488 Goat anti-Rabbit IgG Thermo Fisher Sci Cat# A-11034: manufacturer validation (https://www.thermofisher.com/antibody/product/Goat-anti-Rabbit-IgG-H-L-Highly-Cross-Adsorbed-Secondary-Antibody-Polyclonal/A-11034). Lopez, J., Lim, R., Cruceanu, C. et al. miR-1202 is a primate-specific and brain-enriched microRNA involved in major depression and antidepressant treatment. Nat Med 20, 764–768 (2014).

Alexa Fluor 488 Donkey anti-Mouse IgG Thermo Fisher Sci Cat# A-32766. manufacturer validation (https://www.thermofisher.com/antibody/product/Donkey-anti-Mouse-IgG-H-L-Highly-Cross-Adsorbed-Secondary-Antibody-Polyclonal/A32766). Yang et l., UBR5 promotes antiviral immunity by disengaging the transcriptional brake on RIG-I like receptors (2024). Bittel et al., Voluntary wheel running improves molecular and functional deficits in a murine model of facioscapulohumeral muscular dystrophy (2024).

Alexa Fluor 488 Goat anti-Guinea Pig IgG Thermo Fisher Sci Cat# A-11073. manufacturer validation (https://www.thermofisher.com/antibody/product/Goat-anti-Guinea-Pig-IgG-H-L-Highly-Cross-Adsorbed-Secondary-Antibody-Polyclonal/A-11073). Rylaarsdam et al., iPSC-derived models of PACS1 syndrome reveal transcriptional and functional deficits in neuron activity (2024).

Alexa Fluor 568 Goat anti-Rabbit IgG Thermo Fisher Sci Cat# A-11011. manufacturer validation (https://www.thermofisher.com/antibody/product/Goat-anti-Rabbit-IgG-H-L-Cross-Adsorbed-Secondary-Antibody-Polyclonal/A-11011). lopez et al., Early Post-Natal Immune Activation Leads to Object Memory Deficits in Female Tsc2+/- Mice: The Importance of Including Both Sexes in Neuroscience Research (2024). Ruturaj et al., Regulation of the apico-basolateral trafficking polarity of the homologous copper-ATPases ATP7A and ATP7B (2024).

Alexa Fluor 647 Donkey anti-Rabbit Thermo Fisher Sci #A-31573. manufacturer validation (https://www.thermofisher.com/antibody/product/Donkey-anti-Rabbit-IgG-H-L-Highly-Cross-Adsorbed-Secondary-Antibody-Polyclonal/A-31573). Rylaarsdam et al., iPSC-derived models of PACS1 syndrome reveal transcriptional and functional deficits in neuron activity (2024). Saffari et al., High-content screening identifies a small molecule that restores AP-4-dependent protein trafficking in neuronal models of AP-4-associated hereditary spastic paraplegia (2024).

Alexa Fluor 647 Goat anti-Mouse IgG Thermo Fisher Sci Cat# A-21236: manufacturer validation (https://www.thermofisher.com/antibody/product/Goat-anti-Mouse-IgG-H-L-Highly-Cross-Adsorbed-Secondary-Antibody-Polyclonal/A-21236). Kim, S., Im, S., Oh, S. et al. Anisotropically organized three-dimensional culture platform for reconstruction of a hippocampal neural network. Nat Commun 8, 14346 (2017).

Alexa Fluor 647 Goat anti-Rabbit IgG Thermo Fisher Sci Cat# A-21245: manufacturer validation (https://www.thermofisher.com/antibody/product/Goat-anti-Rabbit-IgG-H-L-Highly-Cross-Adsorbed-Secondary-Antibody-Polyclonal/A-21245). Loo, L., Bougen-Zhukov, N. & Tan, W. Early spatiotemporal-specific changes in intermediate signals are predictive of cytotoxic sensitivity to TNFα and co-treatments. Sci Rep 7, 43541 (2017).

Alexa Fluor 647 Goat anti-Guinea Pig IgG Thermo Fisher Sci Cat# A-21450: manufacturer validation (https://www.thermofisher.com/antibody/product/Goat-anti-Guinea-Pig-IgG-H-L-Highly-Cross-Adsorbed-Secondary-Antibody-Polyclonal/A-21450). Bramini, M., Sacchetti, S., Armirotti, A., et al. Graphene Oxide Nanosheets Disrupt Lipid Composition, Ca2+ Homeostasis, and Synaptic Transmission in Primary Cortical Neurons. ACS Nano 10 (7), 7154-7171 (2016).

Mouse monoclonal anti-AMPK (alpha 1 & alpha 2) Abcam #ab80039. manufacturer validation (https://www.abcam.com/en-de/products/primary-antibodies/ampk-alpha-1-ampk-alpha-2-antibody-342-ab80039#). Liang et al., Therapeutic efficacy of apelin on transplanted mesenchymal stem cells in hindlimb ischemic mice via regulation of autophagy (2016). Tested in WB in mouse tissue.

Rabbit monoclonal anti-phospho-AMPK (Thr172). Cell Signalling #2535. manufacturer validation (https://www.cellsignal.com/products/primary-antibodies/phospho-ampka-thr172-40h9-rabbit-mab/2535). Arumugam et al., Multiomics analyses reveal dynamic bioenergetic pathways and functional remodeling of the heart during intermittent fast (2023). Tested in WB in mouse tissue.

Rabbit monoclonal anti-ATG5 abcam Cat# ab108327: manufacturer validation (https://www.abcam.com/apg5latg5-antibody-epr17552-ab108327.html). Ma, Z., Li, F., Chen, L. et al. Autophagy promotes hepatic differentiation of hepatic progenitor cells by regulating the Wnt/β-catenin signaling pathway. J Mol Hist 50, 75–90 (2019). Tested by WB in mouse cells.

Mouse monoclonal anti-α-Tubulin Synaptic Systems Cat# 302 211: manufacturer validation (https://www.sysy.com/products/tubulin/facts-302211.php). Götzke H et al., The ALFA-tag is a highly versatile tool for nanobody-based bioscience applications. Nature communications (2019). Tested by WB in mouse.

MMouse monoclonal anti-β-Actin Sigma #A5441. manufacturer validation (https://www.sigmaaldrich.com/DE/en/product/sigma/a5441?utm_source=google&utm_medium=cpc&utm_campaign=12410876063&utm_content=120911499323&gclid=CjwKCAiA8YyuBhBSEiwA5R3-EwQrfZixgMCk2DgzzrOHepXVgWtkPERiHJlq1llLzZJZ5--gaEGmMRoCBx8QAvD_BwE).

Rabbit polyclonal anti-GLUT2 ProteinTech #20436-1-AP. manufacturer validation (https://www.ptglab.com/de/products/SLC2A2-Antibody-20436-1-AP.htm). Smith et al., T1R2 receptor-mediated glucose sensing in the upper intestine potentiates glucose

absorption through activation of local regulatory pathways (2018). Used for WB in mouse tissue.

Rabbit polyclonal anti-LC3B Novus Biologicals Cat# NB600-1384: manufacturer validation (https://www.novusbio.com/products/lc3b-antibody_nb600-1384). He X., et al. RNF34 functions in immunity and selective mitophagy by targeting MAVS for autophagic degradation. The EMBO Journal (2019). Tested by WB in mouse cells.

Rabbit monoclonal anti-Vinculin Abcam #ab129002. manufacturer validation (https://www.abcam.com/en-de/products/primary-antibodies/vinculin-antibody-epr8185-ab129002). Kälble et al., Selective Blocking of TNF Receptor 1 Attenuates Peritoneal Dialysis Fluid Induced Inflammation of the Peritoneum in Mice (2016). Heintze et al., Ribose 5-phosphate isomerase inhibits LC3 processing and basal autophagy (2016).

Rabbit anti-Mouse IgG Sigma #A9044. manufacturer validation (https://www.sigmaaldrich.com/DE/en/product/sigma/a9044).

Goat anti-Rabbit IgG Sigma #A0545. manufacturer validation (https://www.sigmaaldrich.com/DE/en/product/sigma/a0545).

Goat anti-Guinea Pig IgG (H+L) peroxidase-conjugated Jackson ImmunoResearch # 106-035-003: manufacturer validation (https://www.jacksonimmuno.com/catalog/products/106-035-003). Saito et al. Autophagy regulates lipid metabolism through selective turnover of NCoR1 (2019)

Rabbit anti-Chicken IgG (H+L) peroxidase-conjugated Millipore AP162P. Effect of divalent cations on the porcine kidney cortex membrane-bound form of dipeptidyl peptidase IV. Pascual et al., The international journal of biochemistry & cell biology 43 2010. Detention of specific yolk IgY in the human oral cavity. David Carlander, Hans Kollberg, Anders Larsson. BioDrugs : clinical immunotherapeutics, biopharmaceuticals and gene therapy 16 433-7 2002

Mouse monoclonal anti-Puromycin Merck MABE343 (https://www.merckmillipore.com/DE/de/product/Anti-Puromycin-Antibody-clone-12D10,MM_NF-MABE343). Immunofluorescence Analysis: A representative lot detected Puromycin-incorporated neosynthesized proteins in WB (Reineke, L. C., et al. (2012). Mol Biol Cell. 23(18):3499-3510.; Trinh, M. A. et al. (2012). Cell Rep. 1(6):678-688.; Fortin, D. A., et al. (2012). J Neurosci. 32(24):8127-8137.;David, A., et al. (2012). J Cell Biol. 197(1):45-57.; David, A., et al. (2011). J Biol Chem. 286(23):20688-20700.; White, L. K., et al. (2011). J Virol. 85(1):606-620.; Hoeffer, C. A., et al. (2011). Proc Natl Acad Sci USA. 108(8):3383-3388.; Schmidt, E., K., et al. (2009). Nat Methods. 6(4):275-277.; Goodman, C. A., et al. (2012). Proc Natl Acad Sci USA. 109(17):E989.; Santini, E., et al. (2013). Nature. 493(7432):411-415.; Quy. P. N., et al. (2013). J Biol Chem. 288(2):1125-1134.).Immunohistochemistry Analysis: A representative lot detecte Puromycin-incorporated neosynthesized protein in IHC (Goodman, C. A., et al. (2010). FASEB J. 25(3):1028-1039.).

Mouse monoclonal anti-RAB11A Proteintech #67902-1-Ig (https://www.ptglab.com/de/products/RAB11A-Antibody-67902-1-Ig.htm).

Mouse monoclonal anti-RAB5 SySy # 108 011 (https://sysy.com/product/108011). The amyloid precursor protein is a conserved Wnt receptor. Liu T, Zhang T, Nicolas M, Boussicault L, Rice H, Soldano A, Claeys A, Petrova I, Fradkin L, De Strooper B, Potier MC, et al. eLife (2021) 10: . 108 011 WB, ICC; tested species: mouse. CtBP1-Mediated Membrane Fission Contributes to Effective Recycling of Synaptic Vesicles. Ivanova D, Imig C, Camacho M, Reinhold A, Guhathakurta D, Montenegro-Venegas C, Cousin MA, Gundelfinger ED, Rosenmund C, Cooper B, Fejtova A, et al. Cell reports (2020) 307: 2444-2459.e7. 108 011 ICC; tested species: mouse. Neuronal lysosomal dysfunction releases exosomes harboring APP C-terminal fragments and unique lipid signatures.
Miranda AM, Lasiecka ZM, Xu Y, Neufeld J, Shahriar S, Simoes S, Chan RB, Oliveira TG, Small SA, Di Paolo G
Nature communications (2018) 91: 291. 108 011 WB, ICC; tested species: mouse.

Mouse monoclonal anti-VPS35 Santa cruz #sc-374372 (https://www.scbt.com/de/p/vps35-antibody-b-5?srsltid=AfmBOopAIA8SYdlxHHEF89TqrZrx-DOOzdzmO5GXUCFcnmVVBm1AnzEx). The mammalian retromer regulates transcytosis of the polymeric immunoglobulin receptor. | Vergɛs, M., et al. 2004. Nat Cell Biol. 6: 763-9. PMID: 15247922. PMID: 35993307 | RAB21 kontrolliert die Autophagie und die zelluläre Energiehomöostase, indem es das Retromer-vermittelte Recycling von SLC2A1/GLUT1 reguliert. | Pei, Y. et al. 2023. Autophagy. 19: 1070-1086. PMID: 36538041 | CLEC16A interagiert mit Retromer und TRIM27, und sein Verlust beeinträchtigt das endosomale Trafficking und die Neuroentwicklung. | Smits, DJ. et al. 2022. Hum Genet.

PPurified mouse anti- eCadherin BD Biosciences #610182 (https://www.bdbiosciences.com/en-de/products/reagents/microscopy-imaging-reagents/immunofluorescence-reagents/purified-mouse-anti-e-cadherin.610182). C3G down-regulation enhances pro-migratory and stemness properties of oval cells by promoting an epithelial-mesenchymal-like process. In International Journal of Biological Sciences on 21 October 2022 by Palao, N., Sequera, C., et al. SYNCRIP Modulates the Epithelial-Mesenchymal Transition in Hepatocytes and HCC Cells. In International Journal of Molecular Sciences on 14 January 2022 by Riccioni, V., Trionfetti, F., et al. Epigenetic targeting of neuropilin-1 prevents bypass signaling in drug-resistant breast cancer. In Oncogene on 1 January 2021 by Abdullah, A., Akhand, S. S., et al.

Mouse polyclonal anti-GLUT2 AdipoGen #AG-25B-0042-C050 (https://adipogen.com/ag-25b-0042-anti-glut2-mouse-pab-in118.html). The loss of GLUT2 expression by glucose-unresponsive beta cells of db/db mice is reversible and is induced by the diabetic environment: B. Thorens, et al.; J. Clin. Invest. 90, 77 (1992)

Mouse monoclonal anti-NeuN Abcam #ab104224 (https://www.abcam.com/en-us/products/primary-antibodies/neun-antibody-1b7-neuronal-marker-ab104224). Frontiers in aging neuroscience 10:20 2018. Atrial Natriuretic Peptide Acts as a Neuroprotective Agent in Models of Parkinson's Disease via Up-regulation of the Wnt/β-Catenin Pathway. Applications: WB, ICC/IF. Arianna Colini Baldeschi et. al. Scientific reports 7:16855 2017. 661W is a retinal ganglion precursor-like cell line in which glaucoma-associated optineurin mutants induce cell death selectively.
Applications: WB

Rabbit anti-D-Serine Origene #AP02025PU-S. Using conjugated D-Serine-KLH, antibody specificity was erformed with an ELISA test by competition experiments with the following compounds:
Compound : Cross-reactivity Ratio(a)
D-Serine-BSA: 1

L-Serine-BSA: 1 / > 50000
D-Cysteine-BSA: 1 / > 50000
D-Alanine-BSA: 1 / > 50000
(a) D-Serine-BSA concentration/unconjugated or conjugated amino acids concentration at half displacement

Eukaryotic cell lines

Policy information about cell lines and Sex and Gender in Research

Cell line source(s)	HEK293T cells
Authentication	DSMZ #ACC 635
Mycoplasma contamination	All cell lines were tested negative for mycoplasma contamination.
Commonly misidentified lines (See ICLAC register)	*Name any commonly misidentified cell lines used in the study and provide a rationale for their use.*

Animals and other research organisms

Policy information about studies involving animals; ARRIVE guidelines recommended for reporting animal research, and Sex and Gender in Research

Laboratory animals	Mus Musculus, C57Bl/6, both sexes, postnatal days P1-3, P7-9, 3-4 weeks ols, 12-13 weeks old, 40-50 weeks old. Mice were maintained in a pathogen-free environment in ventilated polycarbonate cages. Animals were housed in groups of five animals per cage with constant temperature and humidity at 12h/12h light/dark cycles. Food and water were provided ad libitum.
Wild animals	This study did not involve wild animals
Reporting on sex	Initial observations indicated lack of sex-specific effect. In accordance with the SAGER guidelines, we have ensured that both male and female mice were included in all experimental groups. Specifically, our assessment of locomotor performance revealed that both female and male ATG5 cKO mice exhibited a significant increase in foot slips when crossing a narrow 5 mm beam compared to their littermate controls (Fig. S9j,k). Therefore, our study is balanced and does not skew towards one sex.
Field-collected samples	This study did not involve samples collected from the field
Ethics oversight	All animal experiments were reviewed and approved by the ethics committee of the "Landesamtes für Natur, Umwelt- und Verbraucherschutz des Landes Nordrhein-Westfalen", Cologne (AZ 81-02.04.2020.A418, AZ 81-02-.04.2021.A067, AZ 81-02.04.2022.A116, AZ 81-02.04.2021.A067, AZ 81-02.04.2023.VG076, AZ 81-02.04.4.20.021).

Note that full information on the approval of the study protocol must also be provided in the manuscript.

Plants

Seed stocks	n.a.
Novel plant genotypes	n.a.
Authentication	n.a.
