# Supplementary figures and images for: Autophagy regulator ATG5 preserves cerebellar function by safeguarding its glycolytic activity
Source: Nat Metab. 2025 Jan 15;7(2):297–320. doi: 10.1038/s42255-024-01196-4 (PMC11860254; doi:10.1038/s42255-024-01196-4)

Figure 4

n surface biotinylation

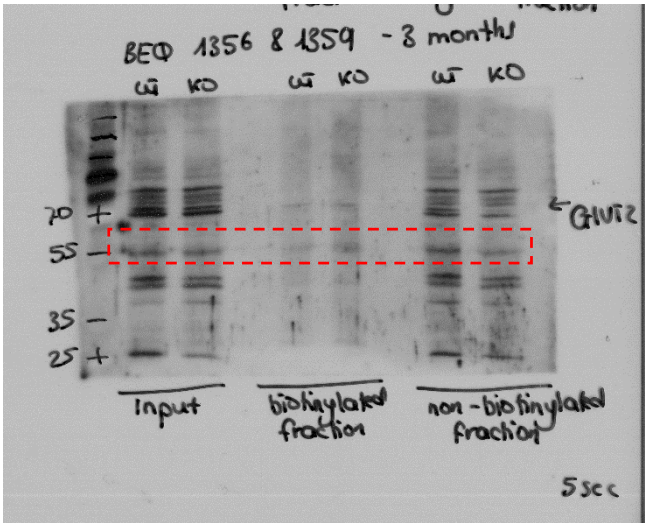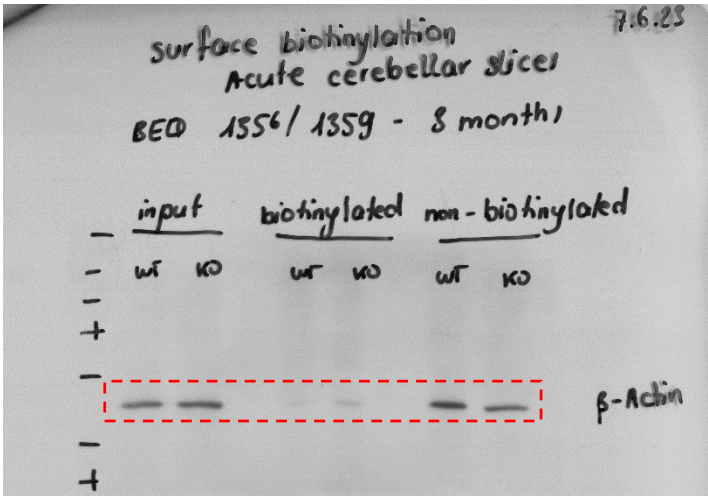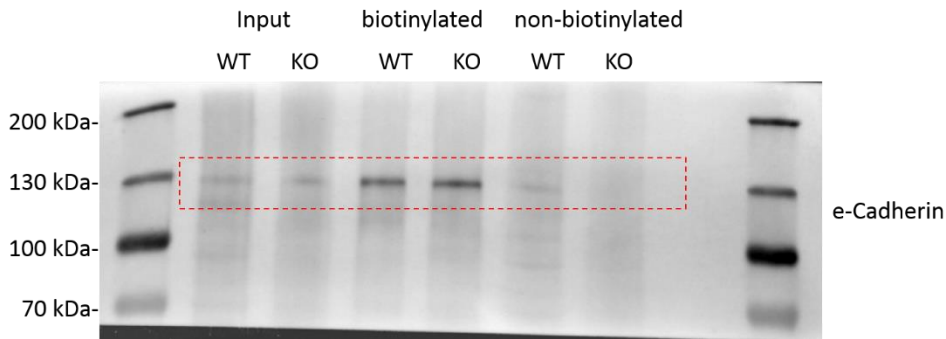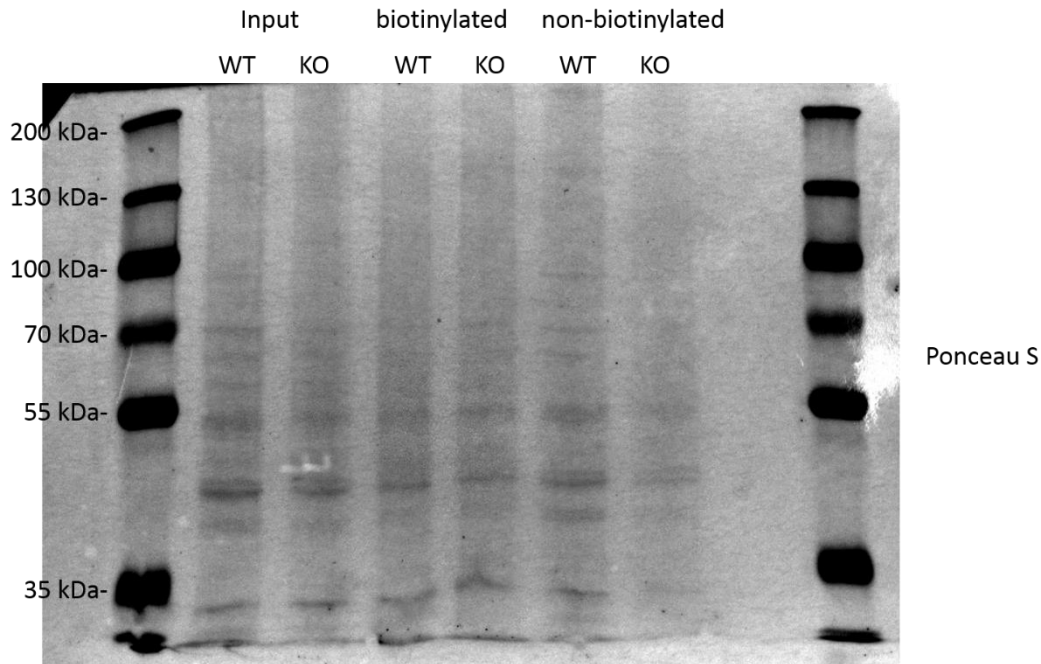

Figure 5

a

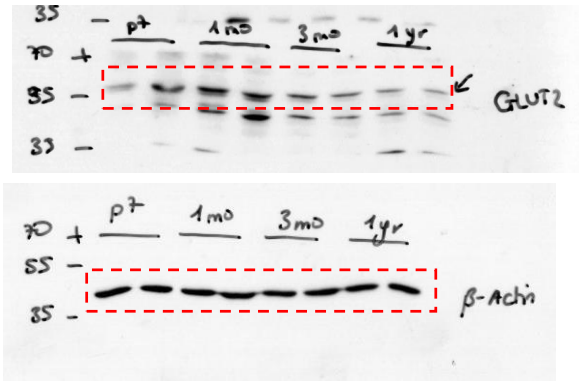

f

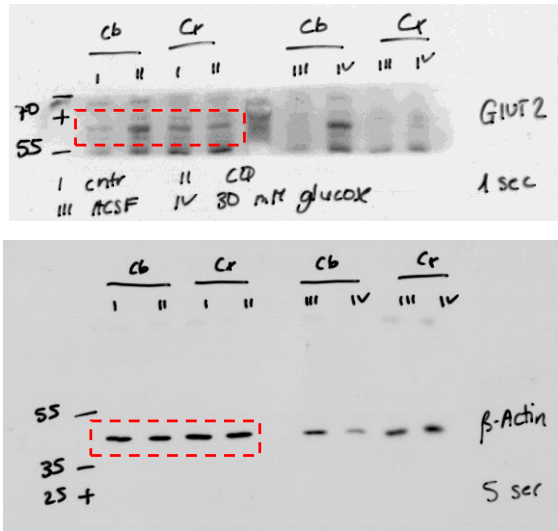

h

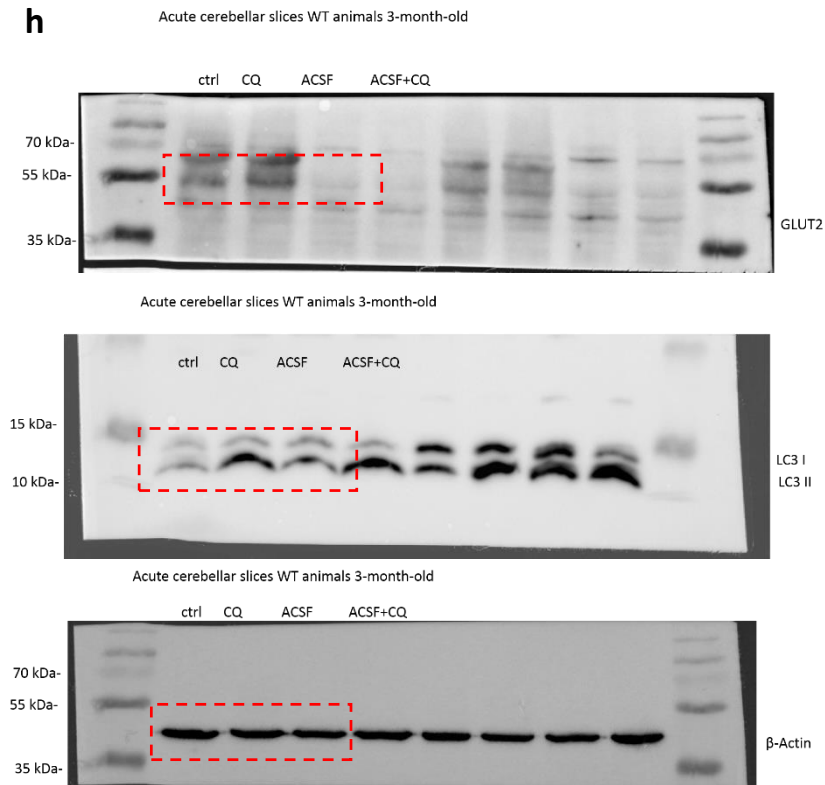

d

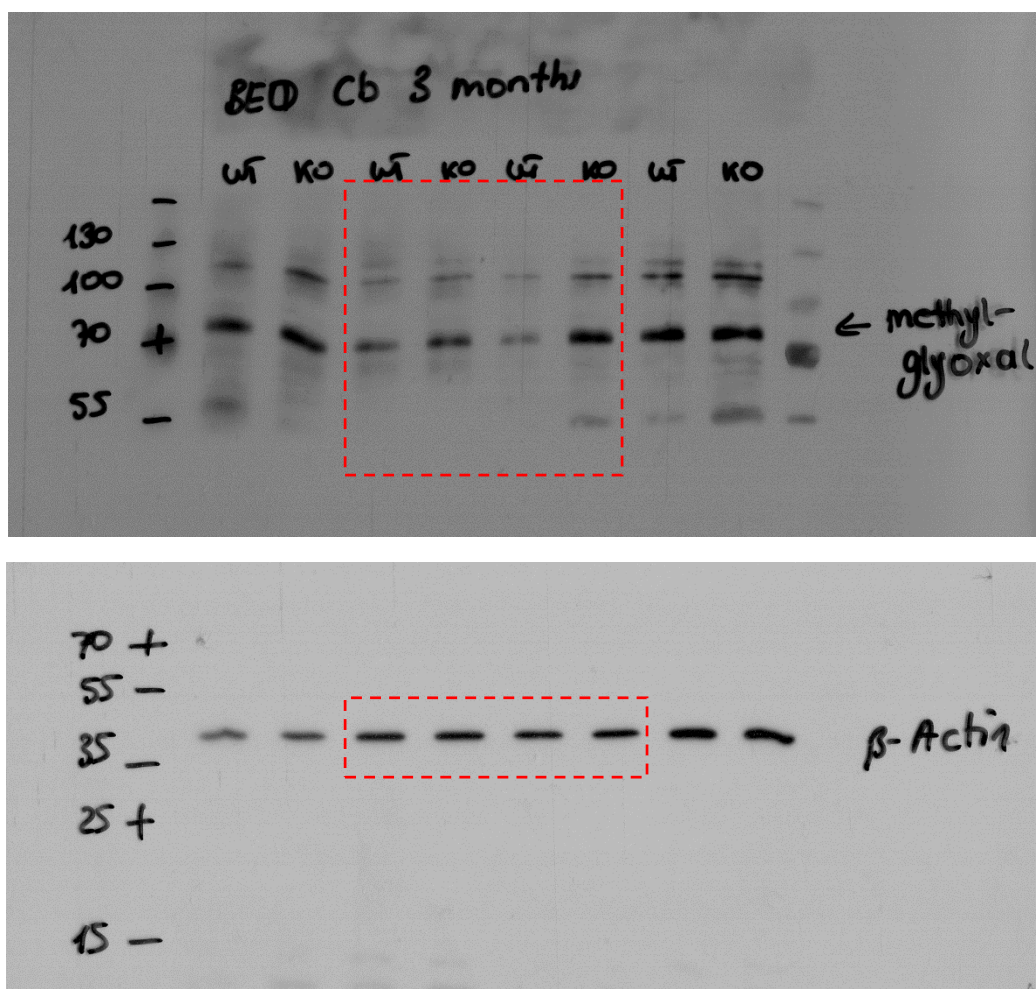

Supplement: Supplementary file 23 — Uncropped immunoblots Figs. 1–7. [file 42255_2024_1196_MOESM23_ESM.pdf]
